# Synthesis and Antiproliferative Activity of Novel Heterocyclic Indole-Trimethoxyphenyl Conjugates

**DOI:** 10.3390/ph10030062

**Published:** 2017-07-05

**Authors:** Michael M. Cahill, Kevin D. O’Shea, Larry T. Pierce, Hannah J. Winfield, Kevin S. Eccles, Simon E. Lawrence, Florence O. McCarthy

**Affiliations:** School of Chemistry, Analytical and Biological Chemistry Research Facility, University College Cork, Western Road, Cork T12 YN60, Ireland; mickcahill72@hotmail.com (M.M.C.); kevin_oshea@umail.ucc.ie (K.D.O.); lpierce9184@yahoo.co.uk (L.T.P.); 106432930@umail.ucc.ie (H.J.W.); kevin.s.eccles@gmail.com (K.S.E.); simon.lawrence@ucc.ie (S.E.L.)

**Keywords:** diarylmaleimide, diaryl-aminopyrazole, 5-aminopyrazole regioselective substitution, drug discovery, NCI anticancer screen

## Abstract

The synthesis and biological evaluation of a series of novel heterocyclic indole derivatives is described. The consolidation of the combretastatin and bisindolylmaleimide templates towards the inclusion of a novel heterocyclic ring proffered a versatile pharmacophore with which to pursue chemical diversification. Given literature precedent, maleimide was initially investigated in this role and the bioactivity assessed by measurement of NCI-60 cell panel growth. Subsequently, a range of 5-aminopyrazoles was designed and developed to explore the specific effect of heterocycle hydrogen bonding on cell growth. The unique electronic nature of the 5-aminopyrazole moiety allowed for regiospecific monosubstitution on different sites of the ring, such as thiourea substitution at the N(1) position for derivative **45** or trifluoroacetylation on the 5-amino position for **43**. Further derivatisation led to the ultimate development of bicyclic pyrazolotriazinedione **41** and pyrimidine **42** systems. The antiproliferative activities of these 3,4-diaryl-5-aminopyrazoles were assessed using the NCI-60 cell screen, disclosing the discovery of distinct selectivity profiles towards a number of cell lines, such as SNB-75 CNS cancer, UO-31 and CAKI-1 renal cancer cells. A series of DNA topological assays discounted the interaction with topoisomerase II as a putative mechanism of action.

## 1. Introduction

**Figure 1:**
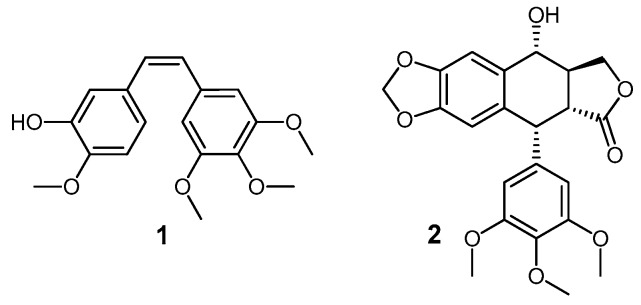
Structures of Combretastatin A4 (CA-4, **1**) and podophyllotoxin **2**.

The 3,4,5-trimethoxyphenyl moiety is a commonly-used fragment in anticancer drug discovery due to its provenance from natural products and their derivatives. One such example is Combretastatin A4 (CA-4, **1**) which was first isolated from the African willow tree, *Combretum caffrum*, by Pettit and co-workers (Figure 1) and is a highly potent tubulin and cell growth inhibitor. The water-soluble prodrug of CA-4, fosbretabulin is currently in clinical trials [1,2,3].

One of the primary mechanisms by which CA-4 **1** or other 3,4,5-trimethoxyphenyl derivatives such as podophyllotoxin (**2**) can mediate anticancer activity is by binding to tubulin, thus preventing microtubule formation and arresting the cell cycle [4]. An additional chemotherapeutic effect of CA-4 **1** is its ability to disrupt established vasculature within solid tumours, while simultaneously rendering normal vascular networks intact [5].

**Figure 2:**
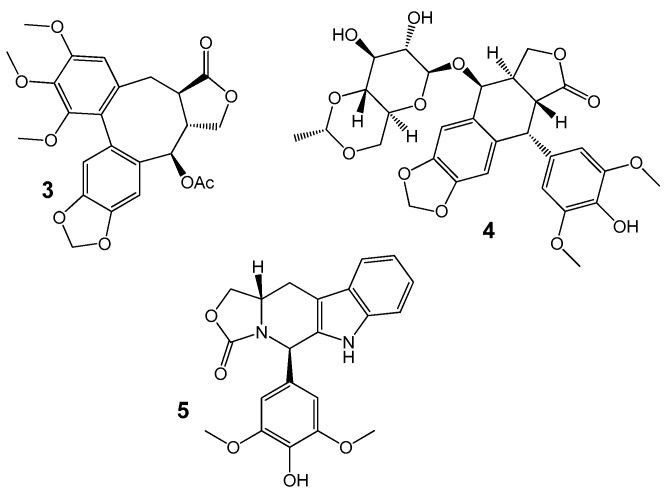
Structures of steganacin (**3**), etoposide (**4**) and azatoxin (**5**).

Examining the structural motif of CA-4, it is established that the 3,4,5-trimethoxyphenyl ring is an essential moiety for cytotoxicity [6]. For example, the 3,4,5-trimethoxyphenyl ring has been found in many other natural products with activities against tubulin, such as steganacin (**3**, Figure 2) [7]. In addition, variants of the moiety are also to be found in other anticancer agents, such as the topoisomerase II (topo II) inhibitors etoposide (**4**) and azatoxin (**5**), which incorporates an indole in design [8,9]. Significantly, at lower concentrations, azatoxin (**5**) is found to exhibit considerable tubulin inhibition, while at higher doses, topo II activity is primarily observed [9]. Alterations to the 3,4,5-trimethoxyphenyl functional group of CA-4 **1**, such as their complete removal or absence, result in loss of potency [10,11].

**Figure 3:**
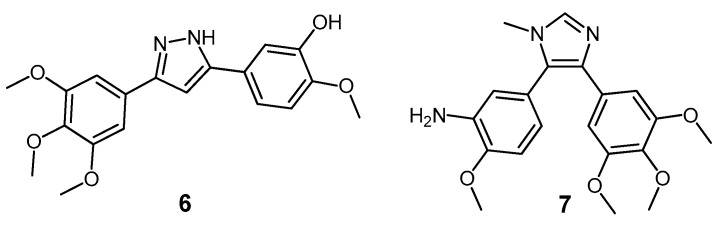
Structures combretastatin derivatives **6** and **7**.

A number of subsequent chemical elaborations have primarily been focused on modifications to the ethene double bond and the 2-methoxyphenol subunit. For example, in many instances, the ethene double bond of CA-4 **1** has been modified towards the inclusion of a heterocyclic functionality, such as a pyrazole in the case of **6** or an imidazole for **7** (Figure 3) and, more recently, tetrazoles, cyclopropylamides and bicyclic thiazolopyrimidinones [12,13,14,15,16]. In other combretastatin analogues, both the ethene double bond and 2-methoxyphenol ring have been replaced with heterocycles, often resulting in the mediation of highly promising chemotherapeutic activity [17].

**Figure 4:**
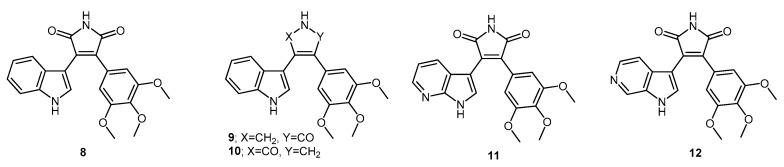
Maleimides **8**, **11** and **12** and pyrrolones **9** and **10**.

In terms of kinase activity, Peifer and co-workers described the synthesis of moguntinone **8** (Figure 4), containing maleimide and indole moieties, which was found to be an extraordinarily potent kinase inhibitor of VEGF-R2 (IC_50_ = 2.5 nM) and VEGF-R3 (IC_50_ = 5 nM) [18]. Modification of the maleimide moiety of **8** towards regioisomeric lactam derivatives produced strikingly different biological profiles. Though pyrrol-2-one **9** was found to potently inhibit VEGF-R2 kinase (IC_50_ = 31 nM), its regioisomeric analogue **10** displayed significantly weaker activity (IC_50_ = 11 µM), thus illustrating the importance of the orientation of the pyrrolone within the active site for the effective exploitation of H-bonding interactions [19].

Elevated levels of angiogenic factors, such as basic fibroblast growth factor, vascular endothelial growth factor (VEGF) and matrix metalloproteinases (MMPs), have been implicated in the promotion of angiogenesis [20,21]. A correlation has previously been demonstrated between high expression of VEGF, which promotes dense formation of tumour microvessels and a poor prognosis in different types of human tumour [22,23].

Recent work on analogous derivatives by Dannhardt et al., such as maleimides **11** and **12** containing 7-azaindolyl and 6-azaindolyl functionalities, respectively, has illustrated potent inhibition of angiogenesis and cell proliferation (Figure 4) [24,25]. In examining the molecular targets associated with the candidates **11** and **12**, significant activity was observed for both against VEGFR-2 and FLT-3 protein kinases. Furthermore, it was found that although 6-azaindolyl **12** was a potent inhibitor of GSK-3β, 7-azaindolyl **11** is completely inactive against the same enzyme. In addition, this group has described selective treatment of colorectal cancer using this template [24,25].

**Figure 5:**
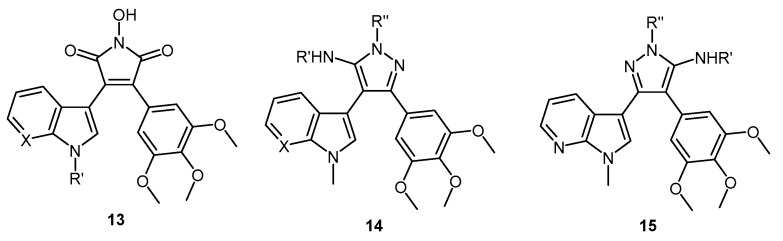
General structures of 3,4-diaryl maleimides **13** and 3,4-diaryl-5-aminopyrazoles **14** and **15**.

Therefore, it is evident that structural diversification of this template can lead to potent anticancer and kinase inhibitory activity. From Structures **2**–**12**, it is obvious that this pharmacophore incorporating an indole, trimethoxyphenyl and a linking headgroup has direct relevance to the disruption of cancer progression. Rationalising the enhancement of existing H-bonding interactions or potential exploitation of new contacts, we embarked on the synthesis of derivatives of the 3,4-diaryl-1-hydroxymaleimide scaffold **13**, in order to probe the effects of oxygen insertion and indole *N*-substitution. Subsequently, replacement of the maleimide/pyrrolone moieties with a closely analogous 5-aminopyrazole ring (**14** along with a regioisomeric series of general structure **15**; Figure 5) was undertaken to validate the approach and explore new chemical space [26,27].

In this work, we report the synthesis, full characterization and initial screening of effects on cancer cell growth to investigate the anticancer activity of this novel template. We also examine the interaction of these derivatives with topoisomerase II as a potential mechanism of action via a series of DNA topological assays, given the inherent activity of azatoxin and etoposide.

## 2. Results and Discussion

### 2.1. Synthesis of the 3,4-Diaryl-1-Hydroxymaleimide Scaffold ***13***

**Scheme 1:**
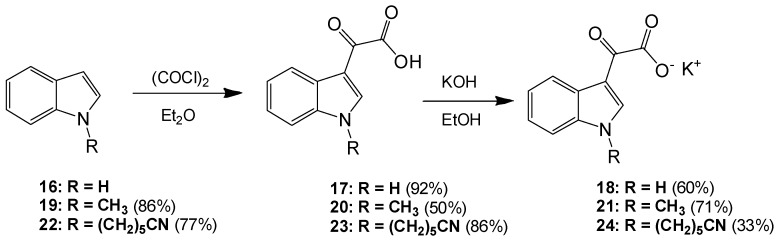
Synthesis of indole potassium glyoxalate salts **18**, **21** and **24**.

**Scheme 2:**
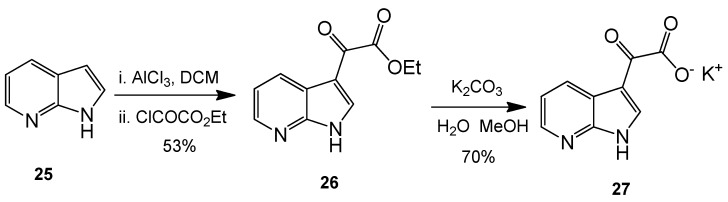
Synthesis of 7-azaindole potassium glyoxalate salt **27**.

The synthesis of a series of 3,4-diaryl-1-hydroxymaleimides was initially undertaken in order to investigate the H-bonding framework required to affect cell growth. Synthesis begins from indole (Scheme 1, **16**), which may be alkylated under standard conditions (**19** and **22**) to investigate the effect of N-H capping and incorporate an element of solubility. This substitution has the possibility to probe two effects: the influence of the indole N-H on binding and a malleable nitrile functional group to modify solubility. Indole potassium glyoxalate salt formation is undertaken in two steps with acylation using oxalyl chloride followed by treatment with base (**18**, **21** and **24**). Difficulties of translation to 7-azaindole necessitated an alternate method, with aluminium trichloride followed by ethyl chlorooxoacetate and then base hydrolysis yielding the glyoxalate (Scheme 2, **27**).

The synthesis of a series of 3,4-diaryl-1-hydroxymaleimides was initially undertaken in order to investigate the H-bonding framework required to affect cell growth. Synthesis begins from indole (Scheme 1, **16**), which may be alkylated under standard conditions (**19** and **22**) to investigate the effect of N-H capping and incorporate an element of solubility. This substitution has the possibility to probe two effects: the influence of the indole N-H on binding and a malleable nitrile functional group to modify solubility. Indole potassium glyoxalate salt formation is undertaken in two steps with acylation using oxalyl chloride followed by treatment with base (**18**, **21** and **24**). Difficulties of translation to 7-azaindole necessitated an alternate method, with aluminium trichloride followed by ethyl chlorooxoacetate and then base hydrolysis yielding the glyoxalate (Scheme 2, **27**).

**Scheme 3:**
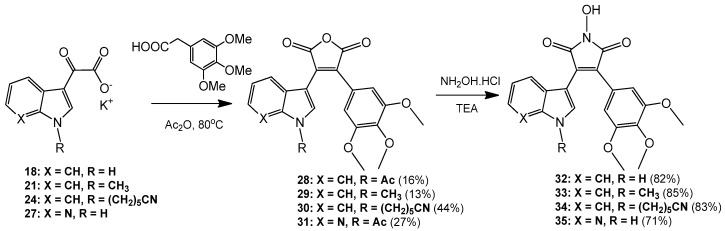
Route to 3,4-diaryl-1-hydroxymaleimides **32**–**35**.

The synthesis of the maleic anhydrides **28**–**31** was facilitated by a modified Perkin condensation with trimethoxyphenyl acetic acid and acetic anhydride in low to moderate yield. Conversion of this anhydride to the 1-hydroxymaleimides **32**–**35** proceeded smoothly and in excellent yield (Scheme 3).

### 2.2. Antiproliferative Activity of 3,4-Diaryl-1-Hydroxymaleimide Derivatives

Assessment of antiproliferative activity was undertaken using the NCI-60 cell screen, where compounds are tested at a single dose (10 µM) against the panel of 60 cancer cells (see Section 2.6 for details). It is evident from the mean growth percentages (Table 1) that there is little inhibition of growth, and on inspection of the individual cell lines, UO-31 appears to be the most susceptible with some limited effect on HOP-92. Substitution of the indole nitrogen or replacement of the indole with 7-azaindole disappointingly led to little difference in activity. Overall, there is minimal effect on cell growth, which led to a revision of our strategy to incorporate greater functionality in the headgroup as seen in Templates **14** and **15**.

### 2.3. Synthetic Routes towards 3,4-Diaryl-5-Aminopyrazoles of General Structure ***15***

**Scheme 4:**
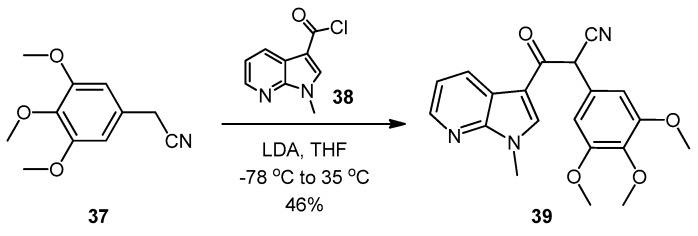
Synthesis of β-ketonitrile **39**.

Synthesis of novel 3,4-diaryl-5-aminopyrazoles **15** to probe the influence of headgroup on cytotoxicity proved relatively straightforward. Initially, β-ketonitrile **39** was fashioned via reaction of acetonitrile **37** with 7-azaindolyl-3-acyl chloride (**38**, Scheme 4).

**Scheme 5:**
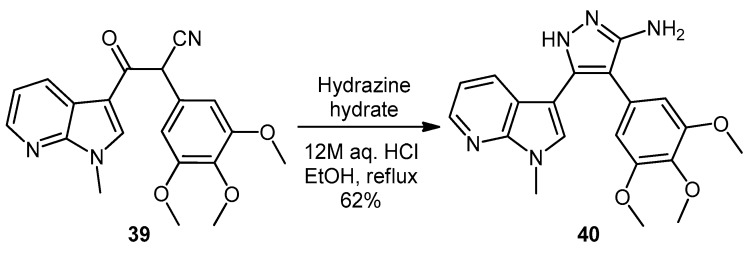
Formation of 5-aminopyrazole **40** from β-ketonitrile **39**.

Reaction of β-ketonitrile **39** with hydrazine hydrate, employing hydrochloric acid as catalyst, in absolute ethanol at reflux temperature led to aminopyrazole **40** (Scheme 5).

**Scheme 6:**
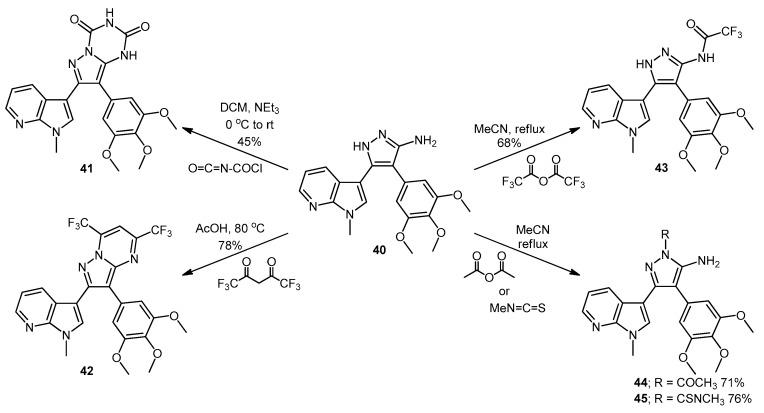
Reaction of aminopyrazole **40** with bidentate and monodentate electrophiles.

The versatile nature of 5-aminopyrazole **40** easily lends itself towards further chemical elaboration of the headgroup space. Our initial plan set out to probe the chemical space by use of hydrophilic and lipophilic components and monosubstitution in tandem with ring formation. Following treatment with bidentate electrophiles *N*-chlorocarbonyl isocyanate and hexafluoroacetylacetone, bicyclic systems **41** and **42** were formed, respectively (Scheme 6).

Structural confirmation of the barbiturate moiety of triazinedione **41** is afforded by the two broad singlets arising from the NH protons at 11.59 and 11.74 ppm in the ^1^H NMR spectrum. A second cyclic headgroup was furnished by condensation of aminopyrazole **40** with hexafluoroacetylacetone in an acidic medium gave highly conjugated pyrazolo[1,5-*a*]pyrimidine **42**.

An intriguing pattern of regioselectivity was observed upon reaction of 5-aminopyrazole **40** with a number of monodentate electrophiles. Reaction with trifluoroacetic anhydride afforded trifluoroacetamide **43** (Scheme 6). The presence of two broad singlets at 11.37 and 13.30 ppm in the ^1^H NMR spectrum confirm substitution on the exocyclic amino functionality rather than at the N(1) nitrogen.

Acetylated Derivative **44** was generated following treatment of aminopyrazole **40** with acetic anhydride in acetonitrile at reflux. In contrast to trifluoroacetamide **43**, the site of substitution for **44** was confirmed to be the N(1) position of the pyrazole, due to the presence of a 2H broad singlet at 5.56 ppm in the ^1^H NMR spectrum corresponding to the exocyclic amino functionality. Reaction of aminopyrazole **40** with methyl isothiocyanate led to the formation of thiourea **45** (Scheme 6). Spectroscopic analysis again provided for structural confirmation of substitution at the N(1) pyrazolic position.

### 2.4. Synthetic Routes to 3,4-Diaryl-5-Aminopyrazoles of General Structure ***14*** [22]

**Scheme 7:**
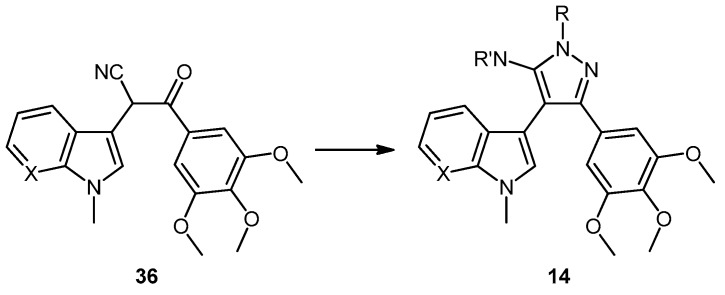
Route to aminopyrazoles **14** via β-ketonitrile **36**.

The synthesis of a series of 3,4-diaryl-5-aminopyrazoles **14** was initiated from β-ketonitriles **36**, which has previously been described (Scheme 7) [26]. Cyclocondensation of Intermediate **36** with hydrazine hydrate under reflux conditions allowed for the synthesis of a highly versatile 5-aminopyrazole core. Subsequent reaction with a range of mono- and bi-dentate electrophiles resulted in the formation of both monosubstituted and bicyclic systems of general Structure **14** (Derivatives **46**–**50**, X = N; **51**–**55**, X = CH; Table 1).

### 2.5. X-ray Crystal Structure Analysis of Substituted 3,4-Diaryl-5-Aminopyrazole Derivatives

Precedence for the difference in regioselectivity observed for the substitution of aminopyrazoles such as **40** exists in the literature [28]. For example, in the development of a series of novel protein kinase inhibitors, Nie et al. described the substitution of substituted 5-aminopyrazoles with ethoxycarbonyl isothiocyanate, with the regioselectivity of the reaction dependant on both the conditions employed and the nature of the ring substituent at the C(4) position [29].

**Figure 6:**
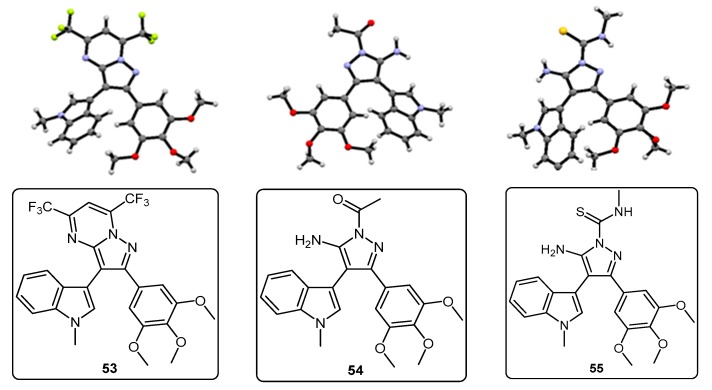
Crystal structures of the bicyclic pyrazolo[1,5-*a*]pyrimidine **53** and monosubstituted aminopyrazoles **54** and **55**.

Therefore, in order to confirm the existence of both monosubstitution and the bicyclic templates, X-ray crystallographic studies were undertaken on a select panel of aminopyrazoles. As can be seen in Figure 6, acetyl aminopyrazole **54** and thiourea **55** demonstrate selective monosubstitution at the N(1) position of the pyrazole ring.

In contrast, pyrazolo[1,5-*a*]pyrimidine **53** is consistent with the structure proposed, confirming the reactivity of both the exocyclic amino functionality and the nitrogen at the N(1) position of the parent aminopyrazole.

Comparing three analogous series of derivatized aminopyrazoles, it is evident that the pattern of regioselectivity with regard to reaction with monodentate electrophiles is not entirely dependent on the position or nature of the aryl substituents. In the case of the reaction with methyl isothiocyanate and acetic anhydride, substitution occurred at the N(1) position of the aminopyrazole ring in each case, i.e., the kinetic product of the reaction was more favourable than the thermodynamic product.

However, when treated under similar conditions with trifluoroacetic anhydride, the most reactive of the three monodentate electrophiles used, substitution occurred on the exocyclic amine of the aminopyrazole (such as with derivative **43**), i.e., the thermodynamic product was the more favourable of the two. In this scenario, it is possible that the kinetic product of the reaction was more unstable than the analogous thiourea and acetyl derivatives, due to the greater electron withdrawing ability of the trifluoroacetyl moiety. Hence, substitution on the amino group may result in the more stable and favoured product.

### 2.6. Antiproliferative Activity of 3,4-Diaryl-5-Aminopyrazole Derivatives

Assessment of antiproliferative activity elicited by the three 5-aminopyrazole series was undertaken using the NCI-60 cell screen, where compounds are tested at a single dose (10 µM) against the panel of 60 cells. Using this platform, distinctive patterns corresponding to differential sensitivities of cell lines to particular compounds can be correlated to the mechanism of cell growth inhibition, and so, it is an important probe in the investigation of mechanistic action in cancer chemotherapy [30].

Examining the first series of 5-aminopyrazoles (**40**–**45**), parent **40** displays appreciable selectivity for HOP-92 lung, SNB-75 CNS and UO-31 and CAKI-1 renal cancer cell lines, all of which display inhibition of growth greater than 50% after 48 h of incubation (Table 1). Extension of aminopyrazole **40** towards bicyclic systems **41** and **42** subsequently resulted in a decrease in overall growth inhibition. In contrast, however, it was found that monosubstitution in the case of acetyl Derivative **44** and thiourea **45** led to both an overall increase in potency against the NCI-60 panel and also an increase in selectivity for UO-31, CAKI-1 renal and SNB-75 CNS cancer cells. For example, an average growth of 32.2% was recorded for thiourea **45** across these three cell lines, compared with a mean growth of 81.1% against the remainder of the panel.

Aminopyrazole **46** and thiourea **50** from the second series (Derivatives **46**–**50**) also display considerable selectivity for the SNB-75, UO-31 and CAKI-1 cell lines, though acetylated derivative **49** is, somewhat surprisingly, relatively inactive by comparison (Table 1). Although conversion of aminopyrazole **46** towards bicyclic Analogues **47** and **48** resulted in an increase in mean growth in both cases, greater selectivity was achieved towards the HOP-92 cell line. This was particularly evident for pyrimidine **48**, which completely arrests growth in this tumour cell line and even exhibits a degree of cytotoxicity (−3.1% growth after 48 h). Utilising the COMPARE algorithm, a high correlation was obtained between the growth inhibition data for three different sets of regioisomers (where one is an identical result): aminopyrazoles **40** and **46** (0.791), pyrimidines **42** and **48** (0.632) and thioureas **45** and **50** (0.805). Given the close agreement found, there exists the likelihood of a common mechanism of action, which provides the scope for further SAR study.

The final series of 3,4-diaryl-5-aminopyrazoles (**51**–**55**), containing an indolyl functionality rather than a 7-azaindolyl subunit, displayed the most potent overall antiproliferative activity across the derivatives tested. In keeping with the trend displayed in the previous two series, parent aminopyrazole **51** and monosubstituted Derivatives **54** and **55** are more potent than their bicyclic Analogues **52** and **53**. In particular, thiourea **55** almost completely arrests growth in HOP-92 lung cancer cells (0.3% growth after 48 h), whilst also exhibiting excellent selectivity for SNB-75 CNS and UO-31 and CAKI-1 renal cancer cells. Once more, this pattern of selectivity compares favourably to that seen for analogous thioureas **45** and **50**. Of great interest also is the fact that aminopyrazole **51**, acetylated Derivative **54** and thiourea **55** exhibit a highly similar level of growth inhibition against the renal cancer cell lines UO-31 and CAKI-1 (Table 1).

### 2.7. Topoisomerase II Decatenation Assay

Consequent to screening the four series for antiproliferative activity, interaction with topoisomerase II of a subset was investigated as a potential mechanism of action. Topo II helps to modulate DNA processes via transient double-strand breaks in the DNA helix [31,32]. With higher expression of topo II in proliferating cells than inactive cells, it is a clinical target for the induction of cytotoxic effects in many cancer types, and inhibitors include the podophyllotoxin (**2**) derivative, etoposide **4**.

Candidates **45**–**50** were screened for their ability to inhibit topoisomerase II-mediated circular kinetoplast DNA (kDNA) decatenation. The standard topo II inhibitor, ellipticine, was used as a reference compound in this study. From the assay, it was apparent that none of the derivatives displayed any activity against topo II (see the Supporting Information). It is possible that the lack of a core planar region in these derivatives prevents intercalation with DNA from occurring, thus eliminating the possibility of topo II inhibition as a function of their antiproliferative activity.

## 3. Materials and Methods

### 3.1. Topoisomerase II Decatenation Assay

The decatenation assay kit was obtained from Inspiralis (Norwich, U.K.) and comprised of the following: (i) 10× assay buffer containing 50 mM Tris-HCl (pH 7.5), 125 mM NaCl, 10 mM MgCl_2_, 5 mM DTT and 100 µg/mL albumin; (ii) dilution buffer containing 50 mM Tris.HCl (pH 7.5), 100 mM NaCl, 1 mM DTT, 0.5 mM EDTA, 50% (*v*/*v*) glycerol, 50 µg/mL albumin; (iii) ATP 30 mM; (iv) kDNA (100 ng/µL); (v) 10 U/µL human topo II in dilution buffer; (vi) 5× stop buffer containing 2.5% SDS, 15% Ficoll-400, 0.05% bromophenol blue, 0.05% xylene cyanol and 25 mM EDTA. Tris-acetate-EDTA buffer (supplied as 10× buffer) and agarose were obtained from Sigma Life Sciences (Dublin, Ireland).

The topo II decatenation assay protocol involved initial incubation of each inhibitor candidate (100 µM) along with a stock solution containing water, ATP, assay buffer, kDNA obtained from the mitochondrial DNA of *Crithidia fasciculate*, and topo II, at 37 °C for 1 h. Following the addition of stop buffer, agarose DNA gel electrophoresis was run at 50 V for 2 h using a Consort EV243 power pack, to determine the relative amounts of decatenated DNA bands obtained in each compound lane. Positive (water), as well as negative controls (ellipticine) were incorporated in order to validate the results of each run [33]. The resulting gels were viewed under UV light using a DNR Bio-Imaging System and photographed using GelCapture software.

### 3.2. NCI-60 Anticancer Screening

The experimental methodology involves initial growth of the tumour cell lines in RPMI 1640 medium containing 5% foetal bovine serum and 2 mM L-glutamine. For a typical screening experiment, cells are inoculated into 96-well microtiter plates in 100 µL of medium at plating densities ranging from 5000–40,000 cells/well, depending on the doubling time of individual cell lines. After cell inoculation, the microtiter plates are incubated at 37 °C, 5% CO_2_, 95% air and 100% relative humidity for 24 h prior to the addition of candidate compounds.

After 24 h, two plates of each cell line are fixed in situ with trichloroacetic acid (TCA), to represent a measurement of the cell population for each cell line at the time of drug addition (time zero (Tz)). Candidate compounds are dissolved in DMSO at 400-fold the desired final maximum test concentration and stored frozen prior to use. The single dose screen is carried out at a concentration of 10 µM.

Following drug addition, the plates are incubated for an additional 48 h at 37 °C, 5% CO_2_, 95% air and 100% relative humidity. For adherent cells, the assay is terminated by the addition of cold TCA. Cells are fixed in situ by the gentle addition of 50 µL of cold 50% (*w*/*v*) TCA and incubated for 60 min at 4 °C. Sulforhodamine B (SRB) solution (100 µL) at 0.4% (*w*/*v*) in 1% acetic acid is added to each well, and plates are incubated for 10 min at room temperature. Absorbance is read on an automated plate reader at a wavelength of 515 nm, and using the absorbance measurements of time zero (Tz), control growth (C) and test growth in the presence of the drug at a concentration of 10 µM, the percentage of growth is calculated [34].

### 3.3. Chemical Data

The data for Compounds **17**–**21** are available in the Supplementary Information.

*6-(1H-Indol-1-yl)hexanenitrile* (**22**). To a solution of indole (2.51 g, 21.4 mmol) in dry DMF (60 mL) at 0 °C was added sodium hydride (1.31 g, 32.75 mmol) in a portion-wise manner. The resultant mixture was allowed to stir at room temperature for 30 min after which time 6-bromohexanitrile (4.25 mL, 1.328 g/mL, 32 mmol) was added with care. The reaction mixture was then allowed to warm to room temperature and stirred overnight. The reaction mixture was subsequently and carefully poured into ice-cold water, and this resulting mixture was extracted with ethyl acetate (6× 50 mL). Combined organic layers were then washed with water (5× 50 mL) and brine (3× 50 mL) before being dried over anhydrous magnesium sulphate and concentrated under reduced pressure to yield a brown oil, which was subject to flash column chromatography (65:35, hexane/ethyl acetate) to yield a viscous yellow oil, which was used without further purification (3.49 g, 16.4 mmol, 77%): ν_max_/cm^−1^ (NaCl) 3053, 2937, 2866, 2244, 1611; δ_H_ (300 MHz, CDCl_3_) 1.43 (m, 2H, CH_2_(CH_2_)_2_CN), 1.61 (m, 2H, CH_2_CH_2_CN), 1.84 (m, 2H, CH_2_(CH_2_)_3_CN), 2.25 (t, 2H, *J* = 7.1 Hz, CH_2_-CN), 4.11 (t, 2H, *J* = 6.9 Hz, N-CH_2_), 6.48 (dd, 1H, *J* = 3.2, 0.86 Hz, C-H_3_), 7.05 (d, 1H, *J* = 3.1 Hz, C-H_2_) 7.09 (overlapping ddd, 1H, *J* = 0.9, 7.1, 7.9 Hz, C-H_5_), 7.19 (m, 1H, *J* = 1.1, 7.1 Hz, C-H_6_), 7.30 (dd, 1H, *J* = 8.3, 0.8 Hz, C-H_7_), 7.62 (dt, 1H, *J* = 7.9, 0.9 Hz, C-H_4_); δ_C_ (75 MHz, CDCl_3_) 17.1 (CH_2_, CH_2_), 25.1 (CH_2_, CH_2_), 26.2 (CH_2_, CH_2_), 29.5 (CH_2_, CH_2_), 46.0 (CH_2_, NCH_2_), 101.3 (CH, aromatic CH), 109.3 (CH, aromatic CH), 119.4 (C, CN), 119.5 (CH, aromatic CH), 121.1 (CH, aromatic CH), 121.5 (CH, aromatic CH), 127.7 (CH, aromatic CH), 128.7 (C, aromatic C), 135.9 (C, aromatic C); *m*/*z* (ES+) 213.4 [M + H]^+^ (100%); HRMS (ES+): exact mass calculated for C_14_H_17_N_2_ 213.1392. Found 213.1386.

*2-(1-(5-Cyanopentyl)-1H-indol-3-yl)-2-oxoacetic acid* (**23**). To a solution of 6-(1*H*-indol-1-yl)hexanenitrile 6 (3.49 g, 16.4 mmol) in diethyl ether (100 mL) at 0 °C was added oxalyl chloride (2.20 mL, 1.48 g/mL, 24.64 mmol) dropwise over a 20 min. The resultant deep orange slurry was allowed to stir at room temperature for 30 min. After this time saturated, aqueous sodium bicarbonate solution was added very carefully (15 mL). The resulting biphasic mixture was left to stir overnight. Following this, the reaction mixture was vacuum filtered, and the product cake was washed with ice-cold diethyl ether (10 mL) and ice-cold water (5 mL) before being allowed to dry to yield the oxoacetic acid as a pink solid (4.03 g, 14.2 mmol, 86%), m.p. 133–135 °C: ν_max_/cm^−1^ (KBr) 3428, 2952, 2241, 1752, 1628, 1520, 1148; δ_H_ (400 MHz, DMSO-*d*_6_) 1.38 (m, 2H, CH_2_(CH_2_)_2_CN), 1.60 (m, 2H, CH_2_CH_2_CN), 1.83 (m, 2H, CH_2_(CH_2_)_3_CN), 2.48 (t, 2H, *J* = 2.4 Hz, CH_2_-CN), 4.34 (t, 2H, *J* = 7.2 Hz, N-CH_2_), 7.33 (m, 2H, C-H_5_, C-H_6_), 7.68 (d, 1H, *J* = 8.1 Hz, C-H_7_), 8.22 (d, 1H, *J* = 8.0 Hz, C-H_4_), 8.52 (s, 1H, C-H_2_); δ_C_ (100 MHz, DMSO-*d*_6_) 16.0 (CH_2_, CH_2_), 24.3 (CH_2_, CH_2_), 25.2 (CH_2_, CH_2_), 28.6 (CH_2_, CH_2_), 46.2 (CH_2_, NCH_2_), 111.3 (CH, aromatic CH), 111.4 (C, aromatic C), 120.6 (C, CN), 121.4 (CH, aromatic CH), 123.0 (CH, aromatic CH), 123.7 (CH, aromatic CH), 126.2 (C, aromatic C), 136.6 (C, aromatic C), 140.3 (CH, aromatic CH), 165.2 (C, aromatic C=O), 180.4 (C, C=O); *m*/*z* (ES+) 285.3 [M + H]^+^ (100%); HRMS (ES+): exact mass calculated for C_16_H_17_N_2_O_3_: 285.1239. Found 285.1236.

*Potassium 2-(1-(5-Cyanopentyl)-1H-indol-3-yl)-2-oxoacetate* (**24**). To a solution of 2-(1-(5-cyanopentyl)-1*H*-indol-3-yl)-2-oxoacetic acid 7 (4.03 g, 14.17 mmol) in ethanol (200 mL) was added potassium hydroxide (0.83 g, 14.8 mmol). The reaction mixture was allowed to stir for 4 h before being isolated by vacuum filtration. The resulting product cake was allowed to dry to yield a beige solid (1.48 g, 4.6 mmol, 33 %), m.p. 167–170 °C: ν_max_/cm^−1^ (KBr) 3406, 2944, 2245, 1884, 1765, 1632 (broad), 1523, 1149; δ_H_ (400 MHz, DMSO-*d*_6_) 1.35 (m, 2H, CH_2_(CH_2_)_2_CN), 1.58 (m, 2H, CH_2_CH_2_CN), 1.79 (m, 2H, CH_2_(CH_2_)_3_CN), 2.47 (t, 2H, *J* = 7.12 Hz, CH_2_-CN), 4.25 (t, 2H, *J* = 7.26, N-CH_2_), 7.19 (m, 2H, C-H_5_, C-H_6_), 7.55 (d, 1H, *J* = 8.09 Hz, C-H_7_), 8.18 (s, 1H, C-H_2_), 8.20 (d, 1H, *J* = 7.58 Hz, C-H_4_); δ_C_ (100 MHz, DMSO-*d*_6_) 16.0 (CH_2_, CH_2_), 24.4 (CH_2_, CH_2_), 25.3 (CH_2_, CH_2_), 28.8 (CH_2_, CH_2_), 45.7 (NCH_2_), 110.5 (CH, aromatic CH), 113.1 (C, CN), 120.6 (C, aromatic C), 121.4 (CH, aromatic CH), 121.5 (CH, aromatic CH), 122.3 (CH, aromatic CH), 126.5 (C, aromatic C), 136.2 (C, aromatic C), 138.3 (CH, aromatic CH), 169.6 (C, C=O), 192.9 (C, C=O); *m*/*z* (ES−) 283.4 [M−H]^−^ (100%); HRMS (ES+): exact mass calculated for C_16_H_17_N_2_O_3_: 285.1239. Found 285.1232.

*Ethyl 2-oxo-2-(1H-pyrrolo[2,3-b]pyridin-3-yl)acetate* (**26**). 7-Azaindole (5.00 g, 42 mmol) was added to a suspension of aluminium trichloride (30.00 g, 225 mmol) in DCM (500 mL), and the resulting mixture was allowed to stir for 30 min at room temperature. After this time, ethyl chlorooxoacetate (24 mL, 215 mmol) was added dropwise over 20 min with the resulting mixture left to stir vigorously at room temperature overnight. The reaction mixture was then poured over ice cold water, and the layers were separated. The aqueous layer was washed with DCM (4× 50 mL). Combined organic layers were then washed very carefully with ice-cold, saturated aqueous sodium bicarbonate solution (3× 20 mL) and brine (1× 100 mL) before being dried over anhydrous magnesium sulphate and concentrated under reduced pressure to yield a brown oil. Purification by flash column chromatography (60:40, hexane/ethyl acetate) afforded the oxoacetate as a fluffy, light yellow solid (4.83 g, 22.1 mmol, 53%), m.p. 157–158 °C; ν_max_/cm^−1^ (KBr) 3418, 1872, 1723, 1611, 1174; δ_H_ (400 MHz, DMSO-*d*_6_) 1.35 (t, 3H, *J* = 7.1 Hz, OCH_2_CH_3_), 4.38 (q, 2H, *J* = 14.2, 7.1 Hz, OCH_2_CH_3_), 7.34 (dd, 1H, *J* = 7.9, 4.7 Hz, C-H_5_), 8.40 (dd, 1H, *J* = 4.7, 1.6 Hz, C-H_6_), 8.47 (dd, 1H, *J* = 7.9, 1.6 Hz, C-H_4_), 8.57 (s, 1H, C-H_2_); *m*/*z* (ES+) 219.3 [M + H]^+^ 100%.

*Potassium 2-oxo-2-(1H-pyrrolo[2,3-b]pyridin-3-yl)acetate* (**27**). To a suspension of ethyl 2-oxo-2-(1*H*-pyrrolo[2,3-*b*]pyridin-3-yl)acetate 9 (4.83 g, 22.1 mmol) in a 1:1 solution of water/MeOH was added potassium carbonate (5.67 g, 41.03 mmol). The resulting mixture was allowed to stir for 5 h before the product was isolated by vacuum filtration and the cake dried overnight to yield the glyoxylate salt as a brilliant white powder (3.53 g, 15.5 mmol, 70%), m.p. > 250 °C; ν_max_/cm^−1^ (KBr) 3244, 3061, 2530, 1980, 1860, 1780, 1634, 1455; δ_H_ (400 MHz, DMSO-*d*_6_) 7.18 (q, 1H, *J* = 7.9, 4.5 Hz, C-H_5_), 8.13 (s, 1H, C-H_2_), 8.27 (dd, 1H, *J* = 4.7, 1.6 Hz, C-H_6_), 8.42 (dd, 1H, *J* = 7.8, 1.6 Hz, C-H_4_); *m*/*z* (ES−) 189.3 [M−H]^−^ 100%.

*3-(1-Acetyl-1H-indol-3-yl)-4-(3,4,5-trimethoxyphenyl)furan-2,5-dione* (**28**). Potassium 2-(1*H*-indol-3-yl)-2-oxoacetate 2 (703 mg, 3.09 mmol) was added to a round-bottomed flask along with 3,4,5-trimethoxyphenylacetic acid (700 mg, 3.09 mmol). Acetic anhydride was added (25 mL) and the reaction mixture was heated for 24 h at 80 °C. Following this, the acetic anhydride was removed under reduced pressure, and to the crude residue was added saturated aqueous sodium bicarbonate solution. This mixture was extracted with ethyl acetate (5× 50 mL). The ethyl acetate was then washed with saturated aqueous sodium bicarbonate solution (4× 50 mL), water (4× 50 mL) and brine (2× 100 mL) before being dried over anhydrous magnesium sulphate and concentrated under reduced pressure to yield a brown residue. Purification by flash chromatography (65:35, hexane/ethyl acetate) yielded a bright yellow solid (216 mg, 0.5 mmol, 16%), m.p. 153–155 °C; ν_max_/cm^−1^ (KBr) 3417, 2942, 1824, 1763, 1718, 1676, 1477; δ_H_ (400 MHz, CDCl_3_) 2.77 (s, 3H, acyl CH_3_), 3.50 (s, 6H, 2× *m*-OCH_3_), 3.89 (s, 3H, *p*-OCH_3_), 6.50 (d, 1H, *J* = 8.1 Hz, C-H_7_), 6.84 (s, 2H, Ar-CH_2′_ and Ar-CH_6′_), 7.03 (overlapping ddd, 1H, *J* = 8.1, 7.6, 0.9 Hz, C-H_5_), 7.34 (overlapping ddd, 1H, *J* = 8.4, 7.3, 1.0 Hz, C-H_6_), 8.23 (s, 1H, C-H_2_), 8.49 (d, 1H, *J* = 8.4 Hz, C-H_4_); δ_C_ (100 MHz, CDCl_3_) 24.0 (CH_3_, Acyl-CH_3_), 56.0 (2× CH_3_, 2× *m*-OCH_3_), 61.0 (CH_3_, *p*-OCH_3_), 108.2 (2× CH, 2× aromatic CH), 110.2 (C, aromatic C), 116.9 (CH, aromatic CH), 122.3 (CH, aromatic CH), 122.7 (C, aromatic C), 124.0 (CH, aromatic CH), 125.4 (C, aromatic C), 126.3 (CH, aromatic CH), 130.0 (C, aromatic C), 130.5 (CH, aromatic CH), 134.5 (C, aromatic C), 135.9 (C, aromatic C), 141.0 (C, aromatic C), 153.0 (2× C, 2× aromatic C), 165.0 (C, C=O), 165.3 (C, C=O), 168.6 (C, C=O); *m*/*z* (ES+) 422.2 [M + H]^+^ (20%); HRMS (ES+): exact mass calculated for C_23_H_20_NO_7_ 422.1240. Found 422.1227.

*3-(1-Methyl-1H-indol-3-yl)-4-(3,4,5-trimethoxyphenyl)furan-2,5-dione* (**29**). Potassium 2-(1-methyl-1*H*-indol-3-yl)-2-oxoacetate 5 (1.43 g, 5.93 mmol) and 3,4,5-trimethoxyphenylacetic acid (1.31 g, 5.8 mmol) were placed in a round-bottomed flask. Acetic anhydride (55 mL) was added, and the resulting mixture was heated for 24 h at 80 °C. Following this, the excess acetic anhydride was removed under reduced pressure, and to the solid residue was added saturated aqueous sodium bicarbonate solution. The crude organic material was extracted with ethyl acetate (4× 50 mL). The combined ethyl acetate washings were then washed with saturated aqueous sodium bicarbonate solution (3× 100 mL), water (3× 100 mL) and brine (2× 100 mL) before being dried over anhydrous magnesium sulphate and concentrated under reduced pressure to yield a sticky red solid. Purification by flash chromatography (70:30, hexane/ethyl acetate) yielded the desired maleic anhydride as a red solid (293 mg, 0.75 mmol, 13 %), m.p. 175–177 °C; ν_max_/cm^−1^ (KBr) 2940, 1987, 1846, 1814, 1748, 1601, 1247, 1124; δ_H_ (400 MHz, CDCl_3_) 3.51 (s, 6H, 2× *m*-OCH_3_), 3.89 (s, 3H, *p*-OCH_3_), 3.92 (s, 3H, N-CH_3_), 6.42 (d, 1H, *J* = 8.2 Hz, C-H_7_), 6.81 (s, 2H, Ar-CH_2′_ and Ar-CH_6′_), 6.90 (overlapping ddd, 1H, *J* = 8.1, 7.2, 0.9 Hz, C-H_5_), 7.21 (overlapping ddd, 1H, *J* = 8.0, 7.1, 0.9 Hz, C-H_6_), 7.35 (d, 1H, *J* = 8.3 Hz, C-H_4_), 8.05 (d, 1H, *J* = 1.3 Hz, C-H_2_); δ_C_ (75 MHz, CDCl_3_) 33.7 (CH_3_, N-CH_3_), 55.9 (2× CH_3_, 2× *m*-OCH_3_), 61.0 (CH_3_, *p*-OCH_3_), 104.2 (C, aromatic C), 107.7 (2× CH, 2× aromatic CH), 110.1 (CH, aromatic CH), 121.3 (CH, aromatic CH), 123.2 (CH, aromatic CH), 123.3 (CH, aromatic CH), 124.1 (C, aromatic C), 124.2 (C, aromatic C), 128.1 (C, aromatic C), 132.5 (C, aromatic C), 136.0 (CH, aromatic CH), 137.5 (C, aromatic C), 139.7 (C, aromatic C), 152.8 (2× C, 2× aromatic C), 165.9 (C, C=O), 166.4 (C, C=O); *m*/*z* (ES+) 394.4 [M + H]^+^ (40%); HRMS (ES+): exact mass calculated for C_22_H_20_NO_6_ 394.1291. Found 394.1283.

*6-(3-(2,5-Dioxo-4-(3,4,5-trimethoxyphenyl)-2,5-dihydrofuran-3-yl)-1H-indol-1-yl)hexanenitrile* (**30**). Potassium 2-(1-(5-cyanopentyl)-1*H*-indol-3-yl)-2-oxoacetate 8 (511 mg, 1.58 mmol) and 3,4,5-trimethoxyphenylacetic acid (365 mg, 1.62 mmol) were added to a round-bottomed flask. Acetic anhydride (15 mL) was added, and the mixture was heated at 80 °C for 24 h. Following this, the excess acetic anhydride was removed under reduced pressure. Saturated aqueous sodium bicarbonate solution was added to the residue to remove any residual acetic anhydride. Crude organic material was then extracted with ethyl acetate (4× 50 mL). The combined ethyl acetate extracts were then washed with saturated aqueous sodium bicarbonate solution (3× 50 mL), water (3× 50 mL) and brine (3× 50 mL) before being dried over anhydrous magnesium sulphate and concentrated under reduced pressure to yield a red residue. Purification by flash chromatography (50:50, hexane/ethyl acetate) yielded the maleic anhydride as a red solid (333 mg, 0.7 mmol, 44%), m.p. 134–136 °C; ν_max_/cm^−1^ (KBr) 3056, 2247, 1815, 1748, 1464; δ_H_ (400 MHz, CDCl_3_) 1.56 (m, 2H, CH_2_(CH_2_)_2_CN), 1.73 (m, 2H, CH_2_CH_2_CN), 1.99 (m, 2H, CH_2_(CH_2_)_3_CN), 2.37 (t, 2H, *J* = 7 Hz, CH_2_CN), 3.52 (s, 6H, 2× *m*-OCH_3_), 3.89 (s, 3H, *p*-OCH_3_), 4.26 ppm (t, 2H, *J* = 7.1 Hz, N-CH_2_), 6.46 (d, 1H, *J* = 8.12 Hz, C-H_7_), 6.81 (s, 2H, Ar-CH_2′_ and Ar-CH_6′_), 6.92 (dt, 1H, *J* = 8.0, 0.9 Hz, C-H_5_), 7.23 (dt, 1H, *J* = 8.0, 0.9 Hz, C-H_6_), 7.37 (d, 1H, *J* = 8.3 Hz, C-H_4_), 8.07 (d, 1H, *J* = 1.46 Hz, C-H_2_); δ_C_ (100 MHz, CDCl_3_) 17.1 (CH_2_, CH_2_), 24.9 (CH_2_, CH_2_), 26.0 (CH_2_, CH_2_), 29.2 (CH_2_, CH_2_), 46.8 (CH_2_, N-CH_2_), 55.9 (2× CH_3_, 2× *m*-OCH_3_), 61.0 (CH_3_, *p*-OCH_3_), 104.5 (C, aromatic C), 107.8 (2× CH, 2× aromatic CH), 110.1 (CH, aromatic CH), 119.2 (C, CN), 121.4 (CH, aromatic CH), 123.4 (2× CH, 2× aromatic CH), 124.0 (C, aromatic C), 124.3 (C, aromatic C), 128.4 (C, aromatic C), 132.4 (C, aromatic C), 134.7 (CH, aromatic CH), 136.7 (C, aromatic C), 139.8 (C, aromatic C), 152.9 (2× C, 2× aromatic C), 165.8 (C, C=O), 166.4 (C, C=O); *m*/*z* (ES+) 475.2 [M + H]^+^ (100%); HRMS (ES+): exact mass calculated for C_27_H_27_N_2_O_6_ 475.1869. Found 475.1863

*3-(1-Acetyl-1H-pyrrolo[2,3-b]pyridin-3-yl)-4-(3,4,5-trimethoxyphenyl)furan-2,5-dione* (**31**). Potassium 2-oxo-2-(1*H*-pyrrolo[2,3-*b*]pyridin-3-yl)acetate 10 (410 mg, 1.8 mmol) and 3,4,5-trimethoxyphenylacetic acid (407 mg, 1.8 mmol) were added to a round-bottomed flask. Acetic anhydride (20 mL) was added and the mixture was heated for 24 h at 80 °C. Following this, the excess acetic anhydride was removed under reduced pressure. Saturated aqueous sodium bicarbonate solution was added to the residue in order to remove any excess acetic anhydride. Crude organic material was then extracted with ethyl acetate (4× 50 mL). The combined ethyl acetate extracts were then washed with saturated aqueous sodium bicarbonate solution (3× 50 mL), water (3× 50 mL) and brine (3× 50 mL) before being dried over anhydrous magnesium sulphate and concentrated under reduced pressure to yield a dull golden residue. Purification by flash chromatography (65:35, hexane/ethyl acetate) yielded the maleic anhydride as a light orange solid (208 mg, 0.49 mmol, 27%), m.p. 103–105 °C; ν_max_/cm^−1^ (KBr) 3311, 1824, 1757, 1625, 1580; δ_H_ (400 MHz, CDCl_3_) 3.12 (s, 3H, acyl CH_3_), 3.58 (s, 6H, 2× *m*-OCH_3_), 3.90 (s, 3H, *p*-OCH_3_), 6.81 (s, 2H, Ar-CH_2′_ and Ar-CH_6′_), 6.88 (dd, 1H, *J* = 8.05, 1.38 Hz, C-H_4_), 7.00 (m, 1H, *J* = 8.0, 4.7 Hz, C-H_5_), 8.38 (dd, 1H, *J* = 4.7, 1.6 Hz, C-H_6_), 8.76 (s, 1H, C-H_2_); δ_C_ (100 MHz, CDCl_3_) 26.1 (CH_3_, Acyl CH_3_), 56.1 (2× CH_3_, 2× *m*-OCH_3_), 61.1 (CH_3_, *p*-OCH_3_), 107.0 (C, aromatic C), 107.9 (2× CH, 2× aromatic CH), 118.9 (CH, aromatic CH), 119.3 (C, aromatic C), 122.7 (C, aromatic C), 130.4 (C, aromatic C), 130.7 (CH, aromatic CH), 131.0 (CH, aromatic CH), 135.0 (C, aromatic C), 141.0 (C, aromatic C), 145.2 (CH, aromatic CH), 147.6 (C, aromatic C), 153.2 (2× C, 2× aromatic C), 164.5 (C, C=O), 164.8 (C, C=O), 168.4 (C, C=O); *m*/*z* (ES+) 423.2 [M + H]^+^ (40%); HRMS (ES+): exact mass calculated for C_22_H_19_N_2_O_7_ 423.1192. Found 423.1189.

Synthesis of *aryl hydroxymaleimides*: General Procedure (i): To a solution of the maleic anhydride (1 eq.) in acetonitrile (or DMF) was added hydroxylamine hydrochloride (5 eq.) and triethylamine (5 eq.). The mixture was heated for 24 h at 80 °C. Following this, 1 M aqueous HCl was added to quench the reaction. Organic components were extracted with ethyl acetate, and the combined extracts were washed with saturated aqueous sodium bicarbonate solution, water, brine, dried over anhydrous magnesium sulphate and concentrated under reduced pressure to yield the corresponding hydroxymaleimide.

*1-Hydroxy-3-(1H-indol-3-yl)-4-(3,4,5-trimethoxyphenyl)-1H-pyrrole-2,5-dione* (**32**). Applying General Procedure (i) starting from maleic anhydride 11 (125 mg, 0.33 mmol), hydroxylamine HCl (115 mg, 1.65 mmol) and triethylamine (0.23 mL, 0.726 g/mL, 1.65 mmol) in acetonitrile (15 mL), hydroxymaleimide 15 was formed as a deep red solid (107.3 mg, 0.27 mmol, 82%), m.p. 123–125 °C; ν_max_/cm^−1^ (KBr) 3262, 2943, 1767, 1711 (broad), 1601, 1562, 1124; δ_H_ (400 MHz, DMSO-*d*_6_) 3.40 (s, 6H, 2× *m*-OCH_3_), 3.69 (s, 3H, *p*-OCH_3_), 6.37 (d, 1H, *J* = 8.05 Hz, C-H_7_), 6.75 (s, 2H, Ar-CH_2′_ and Ar-CH_6′_), 6.79 (t, 1H, *J* = 7.7 Hz, C-H_5_), 7.11 (t, 1H, *J* = 7.6 Hz, C-H_6_), 7.48 (d, 1H, *J* = 8.17 Hz, C-H_4_), 8.05 (s, 1H, C-H_2_), 10.54 (brs, 1H, N-H), 11.98 (brs, 1H, N-OH); δ_C_ (100 MHz, DMSO-*d*_6_) 55.5 (2× CH_3_, 2× *m*-OCH_3_), 60.1 (CH_3_, *p*-OCH_3_), 104.1 (C, aromatic C), 107.6 (2× CH, 2× aromatic CH), 112.2 (CH, aromatic CH), 119.8 (CH, aromatic CH), 121.6 (CH, aromatic CH), 122.2 (CH, aromatic CH), 123.6 (C, aromatic C), 124.6 (C, aromatic C), 125.2 (C, aromatic C), 128.8 (C, aromatic C), 131.6 (CH, aromatic CH), 136.5 (C, aromatic C), 138.2 (C, aromatic C), 152.3 (2× C, 2× aromatic C), 167.6 (C, C=O), 167.9 (C, C=O); *m*/*z* (ES+) 395.6 [M + H]^+^ (10%); HRMS (ES+): exact mass calculated for C_21_H_19_N_2_O_6_ 395.1243. Found 395.1226.

*1-Hydroxy-3-(1-methyl-1H-indol-3-yl)-4-(3,4,5-trimethoxyphenyl)-1H-pyrrole-2,5-dione* (**33**). Applying General Procedure (i) starting from maleic anhydride 12 (257 mg, 0.65 mmol), hydroxylamine HCl (248 mg, 3.25 mmol) and triethylamine (0.45 mL, 0.726 g/mL, 3.25 mmol) in dry DMF (30 mL), hydroxymaleimide 16 was formed as a red solid (226 mg, 0.55 mmol, 85%), m.p. > 250 °C; ν_max_/cm^−1^ (KBr) 3262, 3125, 2942, 1769, 1715 (broad), 1597, 1467, 1125; δ_H_ (400 MHz, DMSO-*d*_6_) 3.40 (s, 6H, 2× *m*-OCH_3_), 3.68 (s, 3H, *p*-OCH_3_), 3.93 (s, 3H, N-CH_3_), 6.32 (d, 1H, *J* = 7.98 Hz, C-H_7_), 6.73 (s, 2H, Ar-CH_2′_ and Ar-CH_6′_), 6.82 (overlapping ddd, 1H, *J* = 8.1, 7.7, 1.0 Hz, C-H_5_), 7.18 (overlapping ddd, 1H, *J* = 8.2, 7.5, 0.9 Hz, C-H_6_), 7.53 (d, 1H, *J* = 8.25 Hz, C-H_4_), 8.11 (s, 1H, C-H_2_), 10.56 (brs, 1H, N-OH); δ_C_ (100 MHz, DMSO-*d*_6_) 33.1 (CH_3_, N-CH_3_), 55.5 (2× CH_3_, 2× *m*-OCH_3_), 59.7 (CH_3_, *p*-OCH_3_), 103.2 (C, aromatic C), 107.7 (2× CH, 2× aromatic CH), 110.6 (CH, aromatic CH), 120.1 (CH, aromatic CH), 121.8 (CH, aromatic CH), 122.3 (CH, aromatic CH), 124.0 (C, aromatic C), 124.3 (C, aromatic C), 125.2 (C, aromatic C), 128.3 (C, aromatic C), 135.2 (CH, aromatic CH), 137.0 (C, aromatic C), 138.2 (C, aromatic C), 152.3 (2× C, 2× aromatic C), 167.6 (C, C=O), 167.8 (C, C=O); *m*/*z* (ES+) 409.2 [M + H]^+^ (30%); HRMS (ES+): exact mass calculated for C_22_H_21_N_2_O_6_ 409.1400. Found 409.1394.

*6-(3-(1-Hydroxy-2,5-dioxo-4-(3,4,5-trimethoxyphenyl)-2,5-dihydro-1H-pyrrol-3-yl)-1H-indol-1-yl)hexanenitrile* (**34**). Applying general procedure (i) starting from maleic anhydride 13 (257 mg, 0.54 mmol), hydroxylamine HCl (188 mg, 2.7 mmol) and triethylamine (0.38 mL, 0.726 g/mL, 2.7 mmol) in dry DMF (30 mL), hydroxymaleimide 17 was formed as a light red solid (219 mg, 0.45 mmol, 83%), m.p. 182–184 °C; ν_max_/cm^−1^ (KBr) 3467, 3213, 3000, 2942, 2260, 1720 (broad), 1626, 1580; δ_H_ (400 MHz, DMSO-*d*_6_) 1.34 (m, 2H, CH_2_(CH_2_)_2_CN), 1.58 (m, 2H, CH_2_CH_2_CN), 1.83 (m, 2H, CH_2_(CH_2_)_3_CN), 2.46 (t, 2H, *J* = 7.03 Hz, CH_2_-CN), 3.34 (s, 6H, 2× *m*-OCH_3_), 3.68 (s, 3H, *p*-OCH_3_), 4.34 (t, 2H, *J* = 6.81 , N-CH_2_), 6.40 (d, 1H, *J* = 8.10 Hz, CH_7_), 6.75 (s, 2H, Ar-CH_2′_ and Ar-CH_6′_), 6.82 (t, 1H, *J* = 7.74 Hz, C-H_5_), 7.17 (t, 1H, *J* = 7.62 Hz, C-H_6_), 7.59 (d, 1H, *J* = 8.21 Hz, C-H_4_), 8.09 (s, 1H, C-H_2_), 10.54 (brs, 1H, N-OH); δ_C_ (100 MHz, DMSO-*d*_6_) 16.0 (CH_2_, CH_2_), 24.2 (CH_2_, CH_2_), 25.1 (CH_2_, CH_2_), 28.7 (CH_2_, CH_2_), 45.6 (CH_2_, N-CH_2_), 55.4 (2× CH_3_, 2× *m*-OCH_3_), 60.1 (CH_3_, *p*-OCH_3_), 103.4 (C, aromatic C), 107.5 (2× CH, 2× aromatic CH), 110.7 (CH, aromatic CH), 120.0 (CH, aromatic CH), 120.5 (C, CN), 121.8 (CH, aromatic CH), 122.3 (CH, aromatic CH), 124.2 (C, aromatic C), 124.7 (C, aromatic C), 125.1 (C, aromatic C), 128.3 (C, aromatic C), 134.1 (CH, aromatic CH), 136.3 (C, aromatic C), 138.2 (C, aromatic C), 152.3 (2× C, 2× aromatic C), 167.5 (C, C=O), 167.8 (C, C=O); *m*/*z* (ES+) 490.2 [M + H]^+^ (70%); HRMS (ES+): exact mass calculated for C_27_H_28_N_3_O_6_ 490.1978. Found 490.1992.

*1-Hydroxy-3-(1H-pyrrolo[2,3-b]pyridin-3-yl)-4-(3,4,5-trimethoxyphenyl)-1H-pyrrole-2,5-dione* (**35**). Applying General Procedure (i) starting from maleic anhydride 14 (147 mg, 0.38 mmol), hydroxylamine HCl (136 mg, 2 mmol) and triethylamine (0.27 mL, 0.726 g/mL, 1.9 mmol) in acetonitrile (15 mL), hydroxymaleimide **35** was formed as an orange solid (106 mg, 0.27 mmol, 71%), m.p. > 250 °C; ν_max_/cm^−1^ (KBr) 3157, 3098, 2937, 1712 (broad), 1616, 1500; δ_H_ (400 MHz, DMSO-*d*_6_) 3.45 (s, 6H, 2× *m*-OCH_3_), 3.70 (s, 3H, *p*-OCH_3_), 6.68 (dd, 1H, *J* = 8.05, 1.54 Hz, C-H_4_), 6.74 (s, 2H, Ar-CH_2′_ and Ar-CH_6′_), 6.88 (m, 1H, *J* = 8.01, 4.69 Hz, C-H_5_), 8.14 (s, 1H, C-H_2_), 8.23 (dd, 1H, *J* = 4.69, 1.53 Hz, C-H_6_), 10.61 (brs, 1H, N-OH), 12.50 (brs, 1H, N-H); δ_C_ (100 MHz, DMSO-*d*_6_) 55.6 (2× CH_3_, 2× *m*-OCH_3_), 60.2 (CH_3_, *p*-OCH_3_), 102.9 (C, aromatic C), 107.6 (2× CH, 2× aromatic CH), 116.1 (CH, aromatic CH), 116.2 (C, aromatic C), 124.9 (C, aromatic C), 126.1 (C, aromatic C), 128.3 (C, aromatic C), 129.5 (CH, aromatic CH), 131.7 (CH, aromatic CH), 138.5 (C, aromatic C), 143.7 (CH, aromatic CH), 148.8 (C, aromatic C), 152.5 (2× C, 2× aromatic C), 167.3 (C, C=O), 167.5 (C, C=O); *m*/*z* (ES+) 396.2 [M + H]^+^ (50%); HRMS (ES+): exact mass calculated for C_20_H_18_N_3_O_6_ 396.1196. Found 396.1181.

*3-(1-Methyl-1H-pyrrolo[2,3-b]pyridin-3-yl)-3-oxo-2-(3,4,5-trimethoxyphenyl)propanenitrile* (**39**). A solution of Lithium diisopropylamide (LDA) (1.8 M, 9.8 mL, 17.7 mmol) in THF (10 mL) was then cooled to −78 °C under a nitrogen atmosphere. A second solution of 3,4,5-trimethoxyphenyl acetonitrile (**37**, 1.36 g, 6.5 mmol) in THF (5 mL) was then added in a dropwise manner, with the resulting mixture stirred for 1 h at −78 °C. A suspension of 1-methyl-1*H*-pyrrolo[2,3-*b*]pyridine-3-carbonyl chloride (**38**, 1.40 g, 7.2 mmol) in THF (25 mL) was slowly added to the reaction mixture over a 20-min period. Once the addition was complete, the reaction mixture was warmed to 35 °C and allowed to stir at this temperature, still under an inert atmosphere, for 12 h. The solvent was evaporated under reduced pressure, and the resultant residue was dissolved in ethyl acetate (40 mL). The organic phase was washed with a saturated aqueous sodium bicarbonate solution (50 mL), water (50 mL) and then brine (50 mL), before being dried over magnesium sulphate. The solvent was removed under reduced pressure, and the crude residue was subjected to flash column chromatography (hexane/ethyl acetate, 30:70) to yield oxopropanenitrile **39** as a yellow solid (1.09 g, 46%): m.p. 137–140 °C; v_max_/cm^−1^ (KBr) 2933, 2243, 1728, 1643, 1595, 1450, 1130; δ_H_ (400 MHz, CDCl_3_) 3.83 [3H, s, *p*-OCH_3_], 3.87 [6H, s, 2× *m*-OCH3], 3.94 [3H, s, NCH_3_], 5.23 [1H, s, CH_α_], 6.70 [2H, s, C-H_2′6′_], 7.28 [1H, q, *J* 7.9, 4.8, C-H_5_], 7.97 [1H, s, C-H_2_], 8.43 [1H, dd, *J* 4.8, 1.6, C-H_6_], 8.60 [1H, dd, *J* 7.9, 1.6, C-H_4_]; δ_C_ (75 MHz, CDCl_3_) 32.3 (CH_3_, NCH_3_), 47.8 (CH, CHCN), 56.4 (2CH_3_, 2× *m*-OCH_3_), 60.9 (CH_3_, *p*-OCH_3_), 105.1 (2CH, 2× aromatic CH), 111.4 (C, aromatic C), 117.3 (C, aromatic C), 119.3 (C, C≡N), 119.4 (CH, aromatic CH), 126.5 (C, aromatic C), 131.2 (CH, aromatic CH), 136.0 (CH, aromatic CH), 138.5 (C, aromatic C), 145.4 (CH, aromatic CH), 148.2 (C, aromatic C), 154.0 (2C, 2× aromatic C), 182.6 (C, C=O); *m*/*z* (ES+) 364.1 [M−H]^−^ (100%); HRMS (ES+): exact mass calculated for C_20_H_20_N_3_O_4_ 366.1454. Found 366.1472.

*5-(1-Methyl-1H-pyrrolo[2,3-b]pyridin-3-yl)-4-(3,4,5-trimethoxyphenyl)-1H-pyrazol-3-amine* (**40**). To a solution of 3-(1-methyl-1*H*-pyrrolo[2,3-*b*]pyridin-3-yl)-3-oxo-2-(3,4,5-trimethoxyphenyl)propanenitrile (**39**, 1.02 g, 2.8 mmol) in absolute alcohol (30 mL) was added 12 M aqueous HCl (1 mL) in a dropwise manner. Hydrazine hydrate (50% aqueous solution, 0.87 mL, 13.9 mmol) was then added, and the resulting mixture was heated to reflux for 26 h. Once cooled to room temperature, the solvent was evaporated under reduced pressure. The residue was dissolved in ethyl acetate (50 mL), then washed successively with saturated aqueous sodium bicarbonate solution (40 mL), water (40 mL) and then brine (40 mL), before being dried over magnesium sulphate. The solvent was removed under reduced pressure, and the crude brown residue was subjected to flash column chromatography (ethyl acetate/methanol, 90:10) to yield pure aminopyrazole **40** as a light brown solid (0.66 g, 62%): m.p. 135–137 °C; v_max_/cm^−1^ (KBr) 3199, 2931, 1602, 1518, 1470, 1351, 1115; δ_H_ (300 MHz, DMSO-*d*_6_) 3.53 [6H, s, 2× *m*-OCH_3_], 3.64 [3H, s, *p*-OCH_3_], 3.82 [3H, s, NCH_3_], 4.68 [2H, bs, NH_2_], 6.53 [2H, s, C-H_2′6′_], 6.98 [1H, q, *J* 7.9, 4.6, C-H_5_], 7.55 [2H, m, C-H_2,4_], 8.24 [1H, dd, *J* 4.6, 1.3, C-H_6_], 11.73 [1H, bs, NH]; δ_C_ (75 MHz, DMSO-*d*_6_) 30.8 (C, NCH_3_), 55.5 (2C, 2× *m*-OCH_3_), 60.0 (C, *p*-OCH_3_), 94.9 (C, broad aromatic C), 106.1 (2CH, 2× aromatic CH), 115.5 (CH, aromatic CH), 117.9 (C, aromatic C), 125.4 (C, broad aromatic C), 128.2 (CH, aromatic CH), 128.8 (CH, aromatic CH), 129.4 (C, aromatic C), 135.4 (C, aromatic C), 141.9 (C, broad aromatic C), 142.7 (CH, aromatic CH), 147.2 (C, aromatic C), 151.8 (C, aromatic C), 152.7 (2C, 2× aromatic C); *m*/*z* (ES+) 380.2 [M + H]^+^ (100%); HRMS (ES+): exact mass calculated for C_20_H_22_N_5_O_3_ 380.1723. Found 380.1724.

*7-(1-Methyl-1H-pyrrolo[2,3-b]pyridin-3-yl)-8-(3,4,5-trimethoxyphenyl)pyrazolo[1,5-a][1,3,5]triazine-2,4(1H,3H)-dione* (**41**). 5-(1-Methyl-1H-pyrrolo[2,3-b]pyridin-3-yl)-4-(3,4,5-trimethoxyphenyl)-1H-pyrazol-3-amine (**40**, 100 mg, 0.26 mmol) was dissolved in DCM (5 mL) containing three drops of triethylamine, and the resulting solution was cooled to 0 °C in an ice bath. This solution was then treated, while stirring, with *N*-chlorocarbonyl isocyanate (0.03 mL, 0.29 mmol). The reaction mixture was subsequently allowed to warm to room temperature and was stirred for 15 h. After careful addition of water (3 mL), a solid precipitated, which was filtered off and then washed with water (4× 10 mL), followed by ether (2× 10 mL). The precipitate was desiccated at 50 °C overnight to give the desired pyrazolotriazinedione **41** as a white solid (51 mg, 45%): m.p. 284–285 °C; v_max_/cm^−1^ (KBr) 3215, 3067, 1761, 1713, 1647, 1586, 1411, 1127; δ_H_ (300MHz, DMSO-*d*_6_) 3.72 [6H, s, 2× *m*-OCH_3_], 3.74 [3H, s, *p*-OCH_3_], 3.78 [3H, s, NCH_3_], 6.64 [2H, s, C-H_2′,6′_], 7.18 [1H, q, *J* 7.8, 4.9, C-H_5_], 7.31 [1H, s, C-H_2_], 8.32-8.34 [2H, m, C-H_4,6_], 11.59 [1H, bs, C=C-NHCO], 11.74 [1H, bs, CONHCO]; δ_C_ (75 MHz, DMSO-*d*_6_) 31.0 (CH_3_, NCH_3_), 55.8 (2CH_3_, 2× *m*-OCH_3_), 60.1 (CH_3_, *p*-OCH_3_), 102.0 (C, aromatic C), 104.8 (C, aromatic C), 107.9 (2CH, 2× aromatic CH), 116.5 (CH, aromatic CH), 118.0 (C, aromatic C), 124.9 (C, aromatic C), 129.5 (CH, aromatic CH), 130.0 (CH, aromatic CH), 137.2 (C, aromatic C), 138.1 (C, aromatic C), 143.3 (CH, aromatic CH), 144.1 (C, aromatic C), 147.2 (C, aromatic C), 148.7 (C, C=O), 149.7 (C, C=O), 153.0 (2C, 2× aromatic C); *m*/*z* (ES+) 447.2 [M−H]^−^ (100%); HRMS (ES+): exact mass calculated for C_22_H_21_N_6_O_5_ 449.1573. Found 449.1587.

*2-(1-Methyl-1H-pyrrolo[2,3-b]pyridin-3-yl)-5,7-bis(trifluoromethyl)-3-(3,4,5-trimethoxyphenyl)pyrazolo[1,5-a]pyrimidine* (**42**). To a stirred solution of 5-(1-methyl-1H-pyrrolo[2,3-*b*]pyridin-3-yl)-4-(3,4,5-trimethoxyphenyl)-1*H*-pyrazol-3-amine (**40**, 60 mg, 0.16 mmol) in acetic acid (5 mL), was added hexafluoroacetylacetone (0.44 mL, 3.2 mmol). The mixture was then heated to 80 °C for 22 h. Once cooled to room temperature, the excess solvent was removed under reduced pressure. The residue was dissolved in ethyl acetate (20 mL), then washed with water (20 mL) and brine (20 mL), before being dried over magnesium sulphate. The solvent was removed under reduced pressure, and the crude residue was subjected to flash column chromatography (hexane/ethyl acetate, 60:40) to yield **42** as an orange solid (69 mg, 78%): m.p. 176–178 °C; v_max_/cm^−1^ (KBr) 2923, 2841, 1586, 1560, 1509, 1279, 1131; δ_H_ (300MHz, CDCl_3_) 3.71 [6H, s, 2× *m*-OCH_3_], 3.81 [3H, s, *p*-OCH_3_], 3.88 [3H, s, NCH_3_], 6.82 [2H, s, C-H_2′6′_], 7.11 [1H, q, *J* 8.0, 4.8, C-H_5_], 7.33 [1H, s, C-H_2_], 7.44 [1H, s, CHCCF_3_], 8.33-8.38 [2H, m, C-H_4,6_]; δ_C_ (75 MHz, CDCl_3_) 30.6 (CH_3_, NCH_3_), 55.1 (2CH_3_, 2× *m*-OCH_3_), 60.0 (CH_3_, *p*-OCH_3_), 100.4 (CH, aromatic CH), 104.3 (C, aromatic C), 106.2 (2CH, 2× aromatic CH), 110.0 (C, aromatic C), 116.2 (CH, aromatic CH), 112.8-123.7 (C, q, *J_F-C_* 274.9, CF_3_), 113.8-124.8 (C, q, *J_F-C_* 274.6, CF_3_), 117.8 (C, aromatic C), 124.5 (C, aromatic C), 129.1 (CH, aromatic CH), 129.7 (CH, aromatic CH), 132.8-134.4 (C, q, *J_F-C_* 39.0, aromatic C), 137.0 (C, aromatic C), 143.0 (CH, aromatic CH), 143.6-145.1 (C, q, *J_F-C_* 38.2, aromatic C), 145.3 (C, aromatic C), 147.0 (C, aromatic C), 151.8 (C, aromatic C), 152.6 (2C, 2× aromatic C); *m*/*z* (ES+) 552.2 [M + H]^+^ (100%); HRMS (ES+): exact mass calculated for C_25_H_20_N_5_O_3_F_6_ 552.1470. Found 552.1493.

*2,2,2-Trifluoro-N-(5-(1-methyl-1H-pyrrolo[2,3-b]pyridin-3-yl)-4-(3,4,5-trimethoxyphenyl)-1H-pyrazol-3-yl)acetamide* (**43**). To a solution of 5-(1-methyl-1H-pyrrolo[2,3-b]pyridin-3-yl)-4-(3,4,5-trimethoxyphenyl)-1*H*-pyrazol-3-amine (**40**, 60 mg, 0.16 mmol) in acetonitrile (10 mL) was added trifluoroacetic anhydride (0.03 mL, 0.19 mmol). The resulting mixture was heated to reflux for 28 h. Once cooled to room temperature, the solvent was evaporated under reduced pressure, and the crude brown residue was subjected to flash column chromatography (hexane/ethyl acetate, 30:70), yielding trifluoroacetamide **43** as an-off white solid (52 mg, 68%): m.p. 129–131 °C; v_max_/cm^−1^ (KBr) 3215, 3067, 1713, 1603, 1586, 1411; δ_H_ (300 MHz, DMSO-*d*_6_) 3.49 [6H, s, 2× *m*-OCH_3_], 3.66 [3H, s, *p*-OCH_3_], 3.91 [3H, s, NCH_3_], 6.53 [2H, s, C-H_2′,6′_], 7.03 [1H, q, *J* 7.9, 4.6, C-H_5_], 7.32 [1H, d, *J* 6.5, C-H_4_], 7.86 [1H, s, C-H_2_], 8.31 [1H, d, *J* 3.5, C-H_6_], 11.37 [1H, bs, NHCOCF_3_], 13.30 [1H, bs, C=N-NH-C]; δ_C_ (75 MHz, DMSO-*d*_6_) 31.0 (CH_3_, NCH_3_), 55.4 (2CH_3_, 2× *m*-OCH_3_), 60.0 (CH_3_, *p*-OCH_3_), 102.0 (C, aromatic C), 105.8 (2CH, 2× aromatic CH), 110.2-121.6 (C, q, *J_F-C_* 288.0, CF_3_), 113.9 (C, aromatic C), 115.9 (CH, aromatic CH), 117.3 (C, aromatic C), 127.1 (C, aromatic C), 128.2 (CH, aromatic CH), 129.4 (CH, aromatic CH), 136.2 (C, aromatic C), 143.1 (CH, aromatic CH), 147.2 (C, aromatic C), 152.7 (2C, 2× aromatic C), 152.9 (C, aromatic C), 155.8-157.2 (C, q, *J_F-C_* 36.2, C=O); *m*/*z* (ES+) 474.3 [M−H]^−^ (100%); HRMS (ES+): exact mass calculated for C_22_H_21_N_5_O_4_F_3_ 476.1546. Found 476.1532.

*1-(5-Amino-3-(1-methyl-1H-pyrrolo[2,3-b]pyridin-3-yl)-4-(3,4,5-trimethoxyphenyl)-1H-pyrazol-1-yl)ethanone* (**44**). To a solution of 5-(1-methyl-1H-pyrrolo[2,3-b]pyridin-3-yl)-4-(3,4,5-trimethoxyphenyl)-1*H*-pyrazol-3-amine (**40**, 60 mg, 0.16 mmol) in acetonitrile (10 mL) was added acetic anhydride (0.016 mL, 0.17 mmol). The resulting mixture was then heated to reflux for 20 h. Once cooled to room temperature, the solvent was removed under reduced pressure. The crude residue was purified by flash column chromatography (hexane/ethyl acetate, 50:50) to yield acetylated product **44** as an off-white solid (48 mg, 71%): m.p. 171–172 °C; v_max_/cm^−1^ (KBr) 3454, 3358, 2927, 1704, 1624, 1509, 1404, 1124; δ_H_ (300 MHz, CDCl_3_) 2.80 [3H, s, NCOCH_3_], 3.79 [3H, s, *p*-OCH_3_], 3.79 [6H, s, 2× *m*-OCH_3_], 3.92 [3H, s, NCH_3_], 5.56 [2H, bs, NH_2_], 6.59 [2H, s, C-H_2′,6′_], 7.05 [1H, s, C-H_2_], 7.14 [1H, q, *J* 7.9, 4.7, C-H_5_], 8.36 [1H, dd, *J* 4.7, 1.5, C-H_6_], 8.47 [1H, dd, *J* 7.9, 1.6, C-H_4_]; δ_C_ (75 MHz, CDCl_3_) 23.3 (CH_3_, NCOCH_3_), 31.4 (CH_3_, NCH_3_), 56.2 (CH_3_, 2× *m*-OCH_3_), 61.0 (CH_3_, *p*-OCH_3_), 101.9 (C, aromatic C), 105.9 (C, aromatic C), 107.1 (CH, 2× aromatic CH), 116.6 (CH, aromatic CH), 118.8 (C, aromatic C), 127.4 (C, aromatic C), 129.3 (CH, aromatic CH), 130.7 (CH, aromatic CH), 137.5 (C, aromatic C), 143.7 (CH, aromatic CH), 147.0 (C, aromatic C), 147.9 (C, aromatic C), 148.5 (C, aromatic C), 153.9 (C, 2× aromatic C), 173.7 (C, C=O); *m*/*z* (ES+) 422.3 [M + H]^+^ (100%); HRMS (ES+): exact mass calculated for C_22_H_24_N_5_O_4_ 422.1828. Found 422.1813.

*5-Amino-N-methyl-3-(1-methyl-1H-pyrrolo[2,3-b]pyridin-3-yl)-4-(3,4,5-trimethoxyphenyl)-1H-pyrazole-1-carbothioamide* (**45**). To a solution of 5-(1-methyl-1*H*-pyrrolo[2,3-*b*]pyridin-3-yl)-4-(3,4,5-trimethoxyphenyl)-1*H*-pyrazol-3-amine (**40**, 60 mg, 0.16 mmol) in acetonitrile (10 mL) was added methyl isothiocyanate (14 mg, 0.19 mmol). The mixture was heated to reflux for 22 h, before then being cooled to room temperature. The solvent was evaporated under reduced pressure, and the crude residue was subjected to flash column chromatography (hexane/ethyl acetate, 50:50), yielding pure thiourea **45** as a white solid (55 mg, 76%): m.p. 162–164 °C; v_max_/cm^−1^ (KBr) 3378, 2931, 1601, 1523, 1406, 1362, 1129; δ_H_ (300 MHz, CDCl_3_) 3.33 [3H, d, *J* 5.0, NHCH_3_], 3.78 [6H, s, 2× *m*-OCH_3_], 3.80 [3H, s, *p*-OCH_3_], 3.92 [3H, s, NCH_3_], 6.47 [2H, bs, NH_2_], 6.59 [2H, s, C-H_2′,6′_], 7.08 [1H, s, C-H_2_], 7.12 [1H, q, *J* 7.9, 4.7, C-H_5_], 8.27 [1H, dd, *J* 7.9, 1.6, C-H_4_], 8.36 [1H, dd, *J* 4.7, 1.5, C-H_6_], 9.24 [1H, bd, *J* 4.7, NHCH_3_]; δ_C_ (75 MHz, CDCl_3_) 30.9 (CH_3_, NHCH_3_), 31.4 (CH_3_, NCH_3_), 56.2 (2CH_3_, 2× *m*-OCH_3_), 61.0 (CH_3_, *p*-OCH_3_), 102.5 (C, aromatic C), 105.4 (C, aromatic C), 107.2 (2CH, 2× aromatic CH), 116.5 (CH, aromatic CH), 118.6 (C, aromatic C), 127.6 (C, aromatic C), 129.7 (CH, aromatic CH), 130.2 (CH, aromatic CH), 137.5 (C, aromatic C), 143.7 (CH, aromatic CH), 145.7 (C, aromatic C), 147.8 (C, aromatic C), 148.1 (C, aromatic C), 153.9 (2C, 2× aromatic C), 176.8 (C, C=S); *m*/*z* (ES+) 453.2 [M + H]^+^ (100%); HRMS (ES+): exact mass calculated for C_22_H_25_N_6_O_3_S 453.1709. Found 453.1718.

## 4. Conclusions

We report the synthesis and evaluation of a novel template for the investigation of the inhibition of cancer cell growth. The synthetic route allows for late stage differentiation to explore the effect of substitution on the headgroup between indole and trimethoxyphenyl subgroups. While disappointing in terms of hydroxymaleimide raw potency, the results of the cell growth inhibition NCI-60 cell screen illustrate the possibility of a similar mode of bioactivity across the four series of 3,4-diaryl heterocyclic derivatives. Appreciable selectivity was recorded for the three parent aminopyrazoles **40**, **46** and **51**. Upon extension of the 5-aminopyrazole core towards bicyclic systems such as triazinedione **41**, the overall mean antiproliferative potency was diminished. However, it was highly notable that conversion of aminopyrazole **46** to pyrimidine **48** resulted in an extraordinary increase in both selectivity and potency against HOP-92 lung cancer cells.

In contrast to their bicyclic analogues, monosubstitution of the 5-aminopyrazole core with thiourea and acetyl functionalities resulted in an increase in overall mean growth inhibition in each case, except for acetylated derivative **49**. Also of particular note is the substituent directed progression seen from **33**–**45**, **50** and finally **55**, the most potent compound of the entire series. Taking the core hydroxymaleimide **33** and converting to a 5-aminopyrazole thiourea **55** yields significant growth inhibition, and the difference in **45** and **50** confirms which orientation is most potent. It is evident that conversion of the headgroup to novel substituted heterocycles can imbue potency and some selectivity in cancer cell lines. This finding is of particular relevance to Compounds **9** and **10** seen earlier, albeit that the substituent orientation that delivers potency against VEGF-R2 kinase is reversed.

Despite the overall mean growth inhibition evident in all three series of aminopyrazole derivatives, the lack of cytotoxicity points to a cytostatic (or cell cycle) mechanism of action. Taking this into account, further SAR study using other monosubstituents is imperative towards the future realization of this class of compounds as chemotherapeutic agents. Similarly, further investigative work will aim to identify the molecular targets associated with the cancer cell lines against which considerable selectivity was observed, namely HOP-92 lung cancer, SNB-75 CNS and UO-31, CAKI-1 renal cancer cell lines. As a result of the topological assays, it is clear topo II inhibition is not a relevant mechanism of action, and to further this, screening of protein kinase and tubulin binding are proposed for the active novel 3,4-diaryl-5-aminopyrazole derivatives.

## Figures and Tables

**Table 1:**
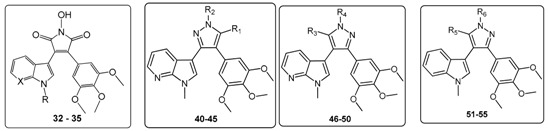
NCI-60 single dose results for 3,4-diaryl-5-aminopyrazoles.

No.	Substituents		NSC No.	Mean (%)	Selected Cells (% Growth)				Renal Cells Mean (%)	Regioisomer Correlation
					HOP-92	SNB-75	CAKI-1	UO-31		
	**X**	**R**								
**32**	CH	H	776698	95.8	89.6	91.0	103.3	71.5	94.9	
**33**	CH	CH_3_	776695	95.4	-	77.9	100.4	66.4	93.4	
**34**	CH	(CH_2_)_5_CN	776696	96.8	69.1	96.3	106.4	77.3	94.9	
**35**	N	H	776697	98.4	83.4	94.0	101.9	86.1	97.3	
	**R_1_**	**R_2_**								
**40**	NH_2_	H	763892	79.8	47.9	34.4	48.9	49.6	76.4	0.791
**41**	-NHCONHCO-		763893	101.5	87.7	67.2	89.8	94.2	100.7	0.366
**42**	-NC(CF_3_)CHC(CF_3_)-		763894	95.4	-	73.2	82.3	71.2	92.6	0.632
**44**	NH_2_	COCH_3_	763896	76.6	-	8.5	39.7	33.1	68.7	0.475
**45**	NH_2_	CSNHCH_3_	763895	78.6	-	26.5	34.1	35.9	71.1	0.805
	**R_3_**	**R_4_**								
**46**	NH_2_	H	754616	75.1	30.9	38.1	50.0	51.7	70.8	0.791
**47**	-NHCONHCO-		754617	104.3	53.5	99.7	118.4	94.8	101.4	0.366
**48**	-NC(CF_3_)CHC(CF_3_)-		754618	94.0	-3.1	67.2	-	73.7	92.5	0.632
**49**	NH_2_	COCH_3_	763899	104.2	86.4	101.9	81.0	92.8	97.6	0.475
**50**	NH_2_	CSNHCH_3_	763898	66.5	-	39.7	18.2	25.3	61.5	0.805
	**R_5_**	**R_6_**								
**51**	NH_2_	H	763905	70.6	-	4.8	18.8	24.6	65.0	
**52**	-NHCONHCO-		763910	75.1	-	47.2	52.9	43.9	80.3	
**53**	-NC(CF_3_)CHC(CF_3_)-		763909	92.9	48.6	56.3	55.2	61.4	87.5	
**54**	NH_2_	COCH_3_	763906	69.4	-	40.3	18.1	23.7	60.6	
**55**	NH_2_	CSNHCH_3_	763907	59.3	0.3	27.8	16.3	17.9	53.2	

## Supplementary Materials

The supplementary material incorporating NMR spectra and NCI data are available online at www.mdpi.com/1424-8247/10/3/62/s1.
