# Supplementary material for: Synthesis and Antiproliferative Activity of Novel Heterocyclic Indole-Trimethoxyphenyl Conjugates
Source: Pharmaceuticals (Basel). 2017 Jul 5;10(3):62. doi: 10.3390/ph10030062 (PMC5620606; doi:10.3390/ph10030062)
Supplement: Supplementary file 1 [file pharmaceuticals-10-00062-s001.pdf]

pharmaceuticals

Supplementary Materials: Synthesis and Antiproliferative Activity of Novel Heterocyclic Indole-Trimethoxyphenyl Conjugates

Michael M. Cahill, Kevin D. O'Shea, Larry T. Pierce, Hannah J. Winfield, Kevin S. Eccles, Simon E. Lawrence and Florence O. McCarthy

<u>**Contents**</u>

- General procedures 2
- Supplementary chemical data 3
- NMR spectra 5
- X-ray crystal structure data 46
- NCI 60 cell screen data 48
- Topoisomerase II inhibition assay 67
- References 68

General Procedures

Solvents were distilled prior to use as follows: dichloromethane was distilled from phosphorous pentoxide; ethyl acetate was distilled from potassium carbonate; ethanol and methanol were distilled from magnesium in the presence of iodine; toluene was distilled from sodium and benzophenone; hexane was distilled prior to use; tetrahydrofuran was freshly distilled from sodium and benzophenone. Diethyl ether was obtained pure from Riedel-de Haën. Organic phases were dried using anhydrous magnesium sulphate. All commercial reagents were used without further purification unless otherwise stated. Infrared spectra were recorded as a thin film on sodium chloride plates for liquids or potassium bromide (KBr) disc for solids on a Perkin Elmer Spectrum 100 FT-IR spectrometer. ^{1}H (300 MHz) and ^{13}C (75 MHz) NMR spectra were recorded on a Bruker Avance 300 NMR spectrometer. ^{1}H (400 MHz) NMR spectra were recorded on a Bruker Avance 400 NMR spectrometer. ^{1}H (600 MHz) and ^{13}C (150 MHz) NMR spectra were recorded on a Bruker Avance III 600 MHz NMR spectrometer equipped with a dual CH cryoprobe. All spectra were recorded at room temperature (~20 °C) in deuterated chloroform ($CDCl_3$) with tetramethylsilane (TMS) as an internal standard, or deuterated dimethylsulfoxide (DMSO-d_6). ^{1}H NMR spectra recorded in deuterated dimethylsulfoxide (DMSO-d_6) were assigned using the DMSO-d_6 peak as the reference peak. Chemical shifts (δ_H and δ_C) are expressed in parts per million (ppm) relative to the reference peak. Coupling constants (*J*) are expressed in Hertz (Hz). Splitting patterns in ^{1}H NMR spectra are designated as s (singlet), br s (broad singlet), d (doublet), br d (broad doublet), t (triplet), q (quartet), dd (doublet of doublets), dt (doublet of triplets), ddd (doublet of doublet of doublets), ddt (doublet of doublet of triplets) and m (multiplet). Low resolution mass spectra were recorded on a Waters Quattro Micro triple quadrupole spectrometer (QAA1202) in electrospray ionisation (ESI) mode using 50% acetonitrile-water containing 0.1% formic acid as eluent. High resolution mass spectra (HRMS) were recorded on a Waters LCT Premier Time of Flight spectrometer (KD160) in electrospray ionisation (ESI) mode using 50% acetonitrile-water containing 0.1% formic acid as eluent. Melting points were measured in a uni-melt Thomas Hoover capillary melting point apparatus and are uncorrected. Thin layer chromatography (TLC) was carried out on precoated silica gel plates (Merck 60 PF254), and visualisation was achieved by UV light detection (254 nm). Single crystal data were collected on either a Bruker SMART X2S diffractometer (**54** and **55**) or a Bruker APEX II DUO diffractometer (**53**) using Mo Kα (λ = 0.7107 Å) radiation, as previously described [1]. Calculations were made using the APEX2 and SHELX software [2,3].

Supplementary Chemical Data

2-(1H-Indol-3-yl)-2-oxoacetic acid (**17**). Indole (**16**, 10.02 g, 85 mmol) was dissolved in diethyl ether (150 mL) and was cooled to 0 °C in an ice bath. Oxalyl chloride (11.0 mL, 1.48 g/mL, 128 mmol) was carefully added drop wise over 20 min. The resulting yellow mixture was allowed to stir at room temperature for 30 min. After this time, the reaction mixture was once again cooled to 0 °C using an ice bath. Saturated, aqueous sodium bicarbonate solution (20 mL) was added very carefully and the resultant red and yellow biphasic mixture was allowed to stir overnight. Following this, the acid was isolated by vacuum filtration washed with ice-cold ether (5 mL) and allowed to air dry to yield the oxoacetic acid (**17**) as a bright yellow solid (14.80 g, 78.2 mmol, 92%); mp 216–217 °C (literature [4]: 213–214 °C), ν_{max}/cm^{-1} (KBr) 3249, 1907, 1790, 1696, 1614, 1505; δ_H (400 MHz, DMSO-*d*$_6$) 7.28 (m, 2H, C-H$_5$, C-H$_6$), 7.55 (dd, 1H, *J* = 6.1, 1.9 Hz, C-H$_7$), 8.18 (dd, 1H, *J* = 6.6, 2.1 Hz, C-H$_4$), 8.42 (d, 1H, *J* = 3.3 Hz, C-H$_2$), 12.34 (brs, 1H, NH); *m*/*z* (ES+) 190.3 $[M + H]^+$ (40%).

Potassium 2-(1H-indol-3-yl)-2-oxoacetate (**18**). To a solution of 2-(1*H*-indol-3-yl)-2-oxoacetic acid 1 (14.75 g, 77.9 mmol) in ethanol (250 mL) was added potassium hydroxide (4.37 g, 78 mmol). The resultant mixture was allowed to stir at room temperature for 4 h, after which time the product was isolated by vacuum filtration and washed with ice cold ethanol (20 mL) to yield a light pink solid (10.64 g, 46.8 mmol, 60%), m.p. > 250°C; ν_{max}/cm^{-1} (KBr) 3597, 3393, 2547, 1787, 1596, 1500; δ_H (400 MHz, DMSO-*d*$_6$) 7.14 (m, 2H, C-H$_5$, C-H$_6$), 7.46 (dd, 1H, *J* = 6.8, 1.4 Hz, C-H$_7$), 8.09 (d, 1H, *J* = 2.8 Hz, C-H$_2$), 8.17 (dd, 1H, *J* = 7.1, 1.6 Hz C-H$_4$), 12.2 (brs, 1H, NH); δ_C (100 MHz, DMSO-*d*$_6$) 112.1 (CH, aromatic CH), 113.9 (C, aromatic C), 121.9 (2× CH, 2× aromatic CH), 122.2 (CH, aromatic CH), 125.9 (C, aromatic C), 135.8 (C, aromatic C), 136.5 (CH, aromatic CH), 170.0 (C, C=O), 193.1 (C, C=O); *m*/*z* (ES−) 188.4 $[M-H]^-$ (100%).

1-Methyl-1H-indole (**19**). To a solution of indole (10.20 g, 87.06 mmol) in dry DMF (200 mL) at 0°C was added sodium hydride (5.20 g, 130 mmol) in a portion-wise manner. The resultant mixture was allowed to stir for 30 min, after which time a solution of methyl iodide (8.30 mL, 133.3 mmol) in dry DMF (20 mL) was added dropwise. The reaction mixture was then allowed to stir overnight. Following this, the reaction mixture was carefully poured into ice-cold water and this was then extracted with ethyl acetate (4× 50 mL). Combined ethyl acetate layers were then washed with 1 M aqueous HCl (4× 50 mL), water (5× 50 mL) and brine (4× 50 mL) before being dried over anhydrous magnesium sulphate and concentrated under reduced pressure to yield the product as a clear oil which was used without further purification (9.79 g, 74.6 mmol, 86%); ν_{max}/cm^{-1} (NaCl) 3056, 2917, 1614, 1515; δ_H (400 MHz, $CDCl_3$) 3.76 (s, 3H, NCH_3), 6.48 (dd, 1H, *J* = 3.1, 0.8 Hz, C-H$_3$), 7.03 (d, 1H, *J* = 3.1 Hz, C-H$_2$), 7.10 (t, 1H, *J* = 7.0 Hz, C-H$_5$), 7.22 (t, 1H, *J* = 7.0 Hz, C-H$_6$), 7.31 (d, 1H, *J* = 8.1 Hz, C-H$_7$), 7.62 (d, 1H, *J* = 7.9 Hz, C-H$_4$); *m*/*z* (ES+) 132.3 $[M + H]^+$ (50%).

2-(1-Methyl-1H-indol-3-yl)-2-oxoacetic acid (**20**). To a solution of 1-methyl-1*H*-indole 3 (9.79 g, 74.64 mmol) in diethyl ether (150 mL) cooled to 0 °C was added oxalyl chloride (9.85 mL, 1.48 g/mL, 112 mmol) dropwise over 20 min. The resultant deep yellow slurry was allowed to stir at room temperature for 30 min before saturated aqueous sodium bicarbonate solution (20 mL) was added with care. The resultant biphasic mixture was allowed to stir overnight. Following this, the product was isolated by vacuum filtration to yield the oxoacetic acid as a cream coloured solid (7.60 g, 37.4 mmol, 50%), m.p. 158–161 °C (literature [5]: 155–158 °C) ν_{max}/cm^{-1} (KBr) 3495, 3250, 3145, 1754, 1618, 1308; δ_H (400 MHz, DMSO-*d*$_6$) 3.92 (s, 3H, N-CH$_3$), 7.34 (m, 2H, C-H$_5$, C-H$_6$), 7.61 (d, 1H, *J* = 7.8 Hz, C-H$_7$), 8.20 (d, 1H, *J* = 7.2 Hz, C-H$_4$), 8.49 (s, 1H, C-H$_2$), 13.91 (brs, 1H, OH); *m*/*z* (ES+) 204.3 $[M + H]^+$ (100%).

Potassium 2-(1-methyl-1H-indol-3-yl)-2-oxoacetate (**21**). To a solution of 2-(1-methyl-1*H*-indol-3-yl)-2-oxoacetic acid 4 (7.60 g, 37.4 mmol) in ethanol (100 mL) was added potassium hydroxide (2.09 g, 37.4 mmol). The resultant mixture was allowed to stir at room temperature for 4 h before the product was isolated by vacuum filtration. The product cake was then washed with ice cold ethanol (10 mL) and dried overnight to yield the potassium salt as a white solid (6.44 g, 26.7 mmol, 71 %), m.p. > 250 °C; ν_{max}/cm^{-1} (KBr) 3416, 3048, 2908, 2535, 1930, 1717, 1650; δ_H (400 MHz, DMSO-*d*$_6$) 3.85 (s, 3H, N-CH$_3$), 7.23

(m, 2H, C-H_5, C-H_6), 7.51 (d, 1H, *J* = 8.0 Hz, C-H_7), 8.18 (d, 1H, *J* = 7.4 Hz, C-H_4) 8.19 (s, 1H, C-H_2); δ_C (100 MHz, DMSO-d_6) 32.9 (CH_3, N-CH_3), 110.3 (CH, aromatic CH), 112.9 (C, aromatic C), 121.5 (2× CH, 2× aromatic CH), 122.3 (CH, aromatic CH), 126.3 (C, aromatic C), 137.0 (C, aromatic C), 139.3 (CH, aromatic CH), 169.7 (C, C=O), 192.7 (C=O); *m*/*z* (ES−) 202.4 $[M-H]^-$ (100%).

NMR Spectra

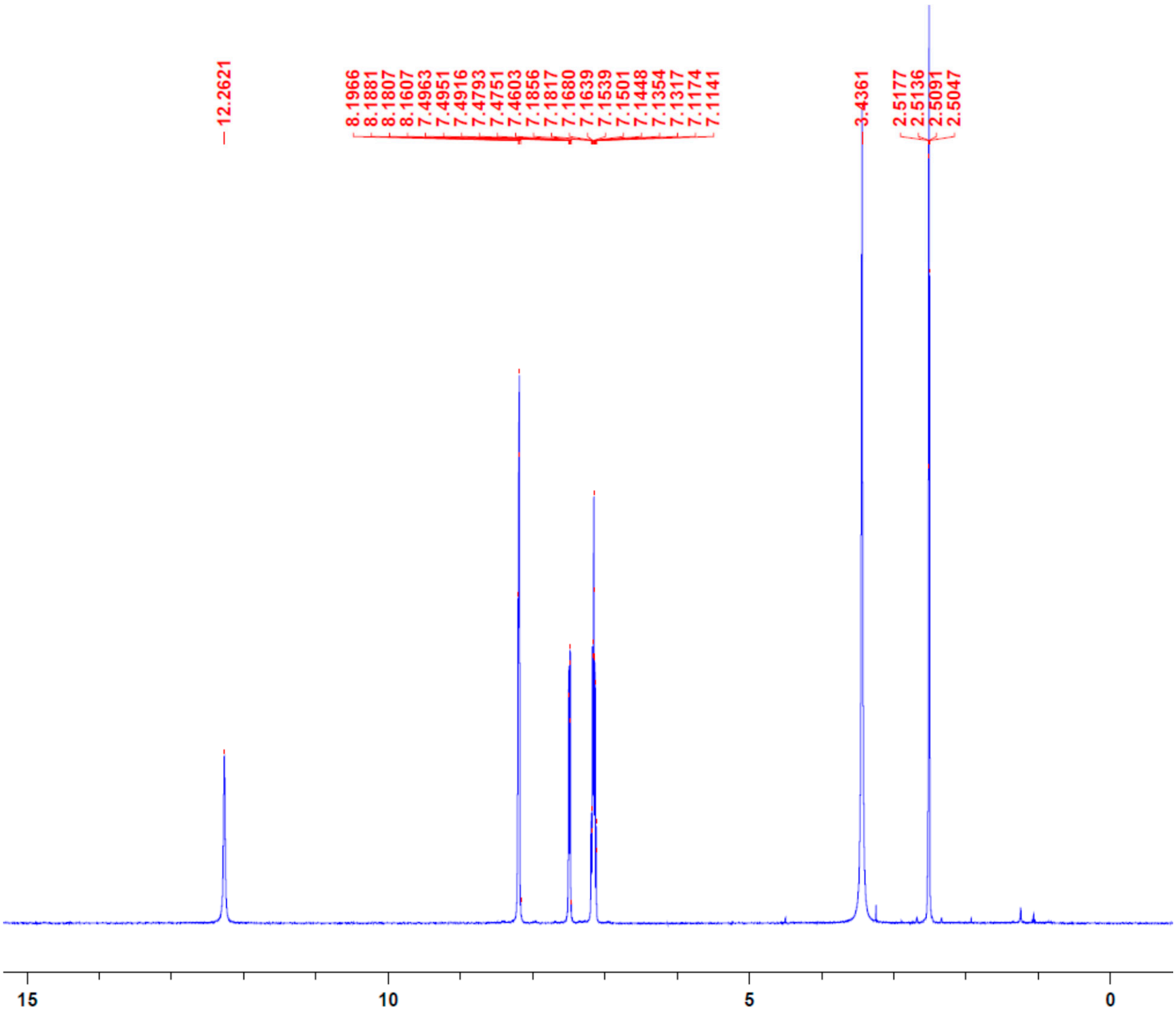

^{1}H NMR of **18.**

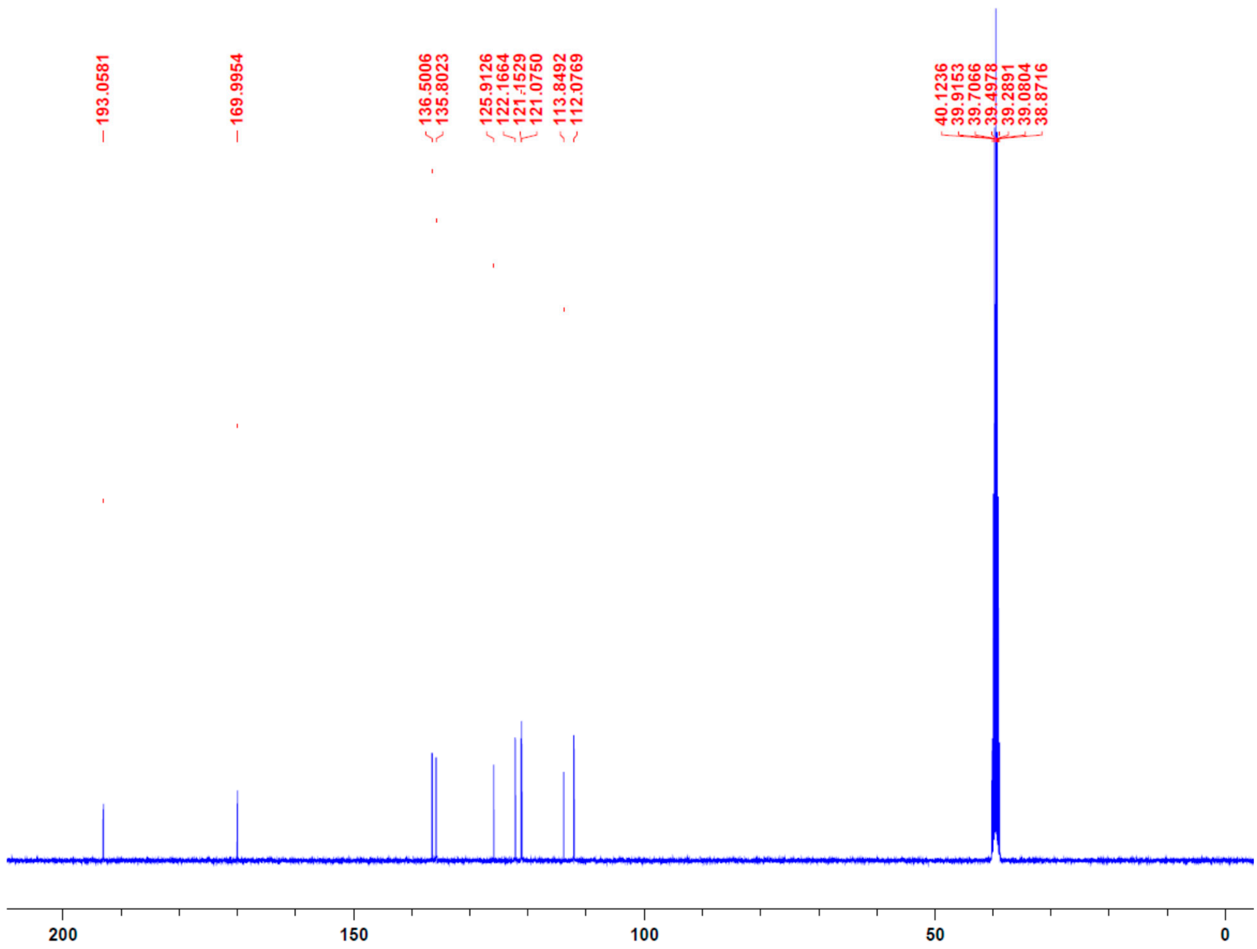

193.0581
169.9954
136.5006
135.8023
125.9126
122.1664
121.1529
121.0750
113.8492
112.0769
40.1236
39.9153
39.7066
39.4978
39.2891
39.0804
38.8716
200
150
100
50
0

^{13}C NMR of **18**

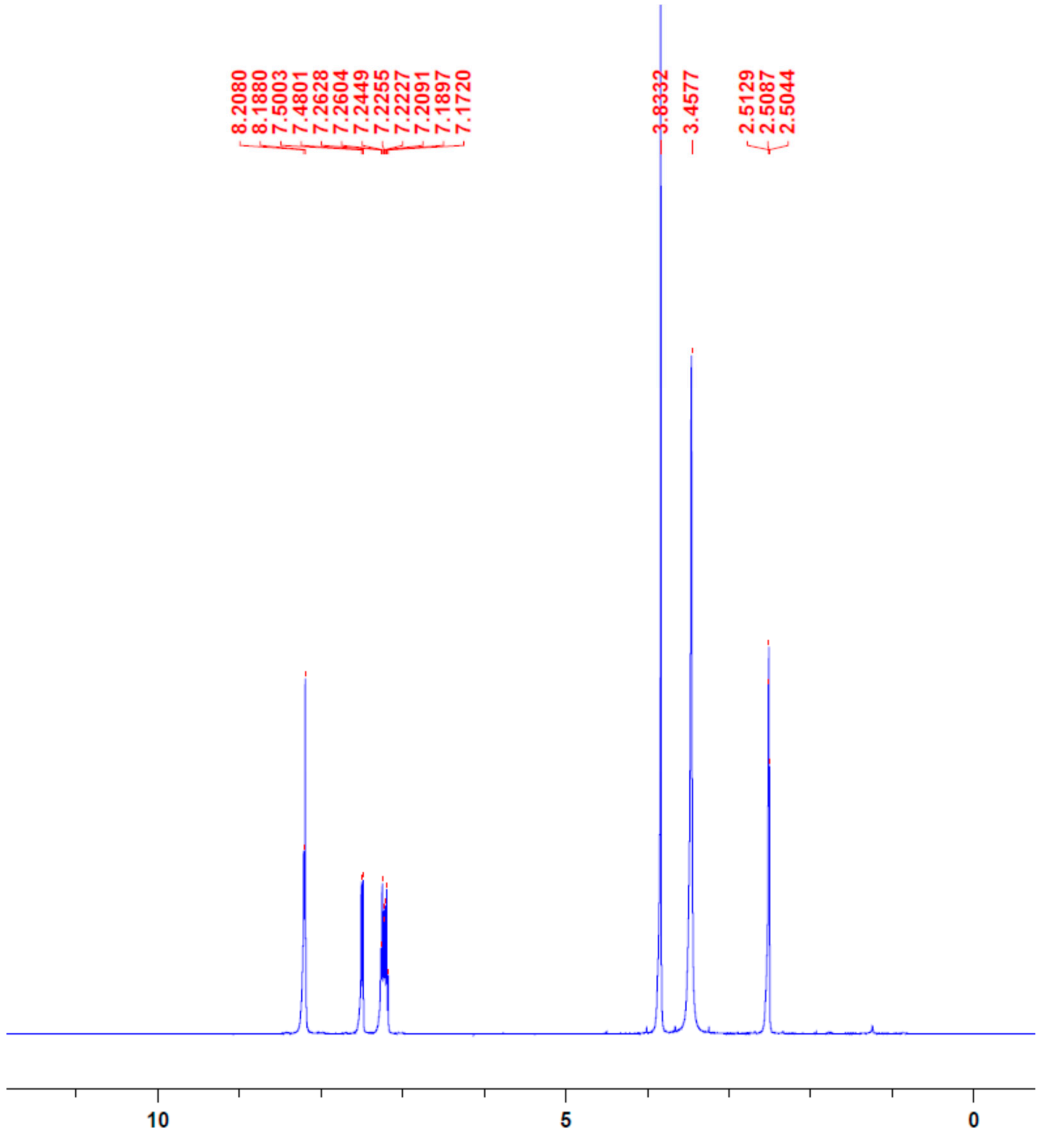
8.2080
8.1880
7.5003
7.4801
7.2628
7.2604
7.2449
7.2255
7.2227
7.2091
7.1897
7.1720
3.8332
3.4577
2.5129
2.5087
2.5044
10
5
0

^{1}H NMR of **21**

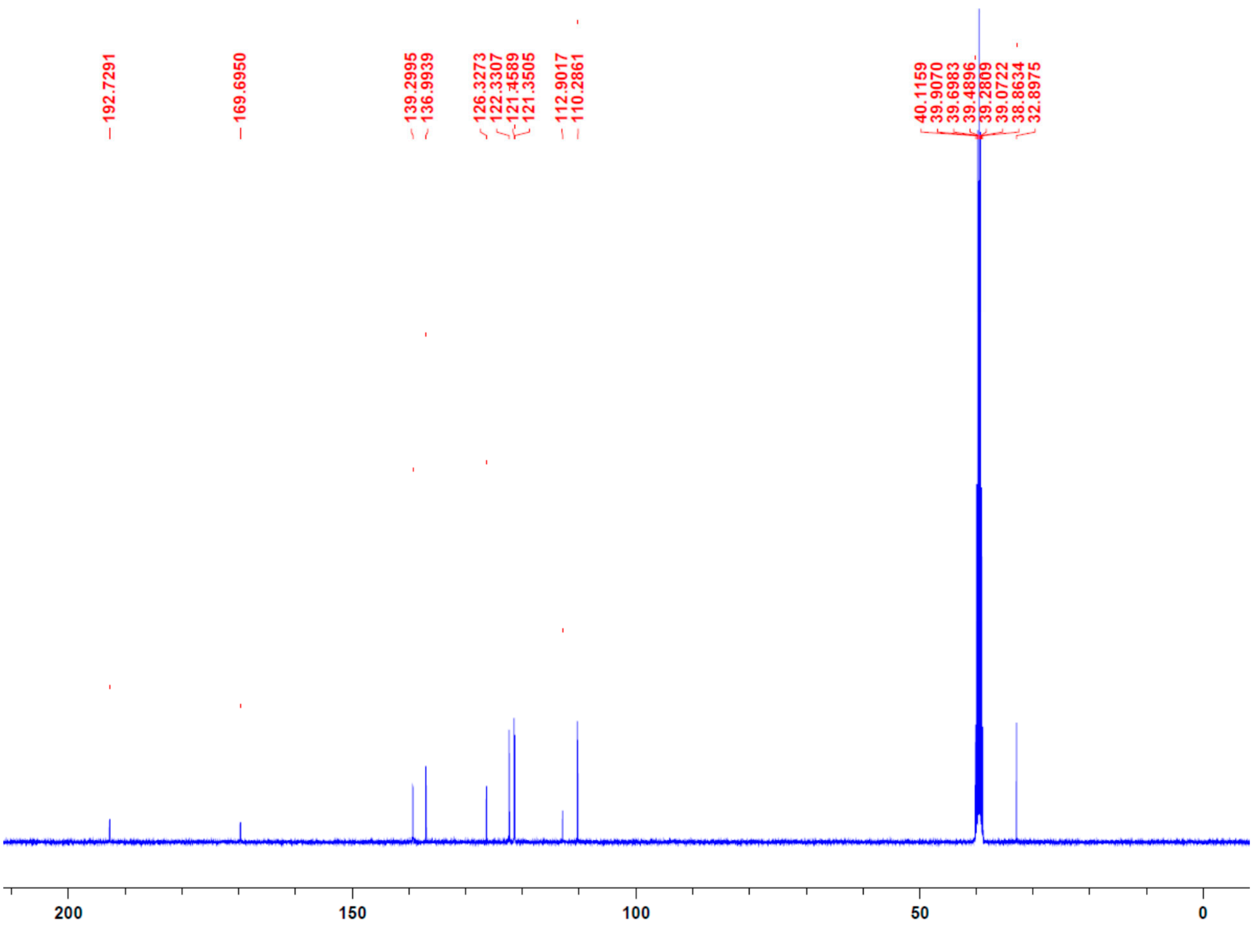
192.7291
169.6950
139.2995
136.9939
126.3273
122.3307
121.4589
121.3505
112.9017
110.2861
40.1159
39.9070
39.6983
39.4896
39.2809
39.0722
38.8634
32.8975
200
150
100
50
0

^{13}C NMR of **21**

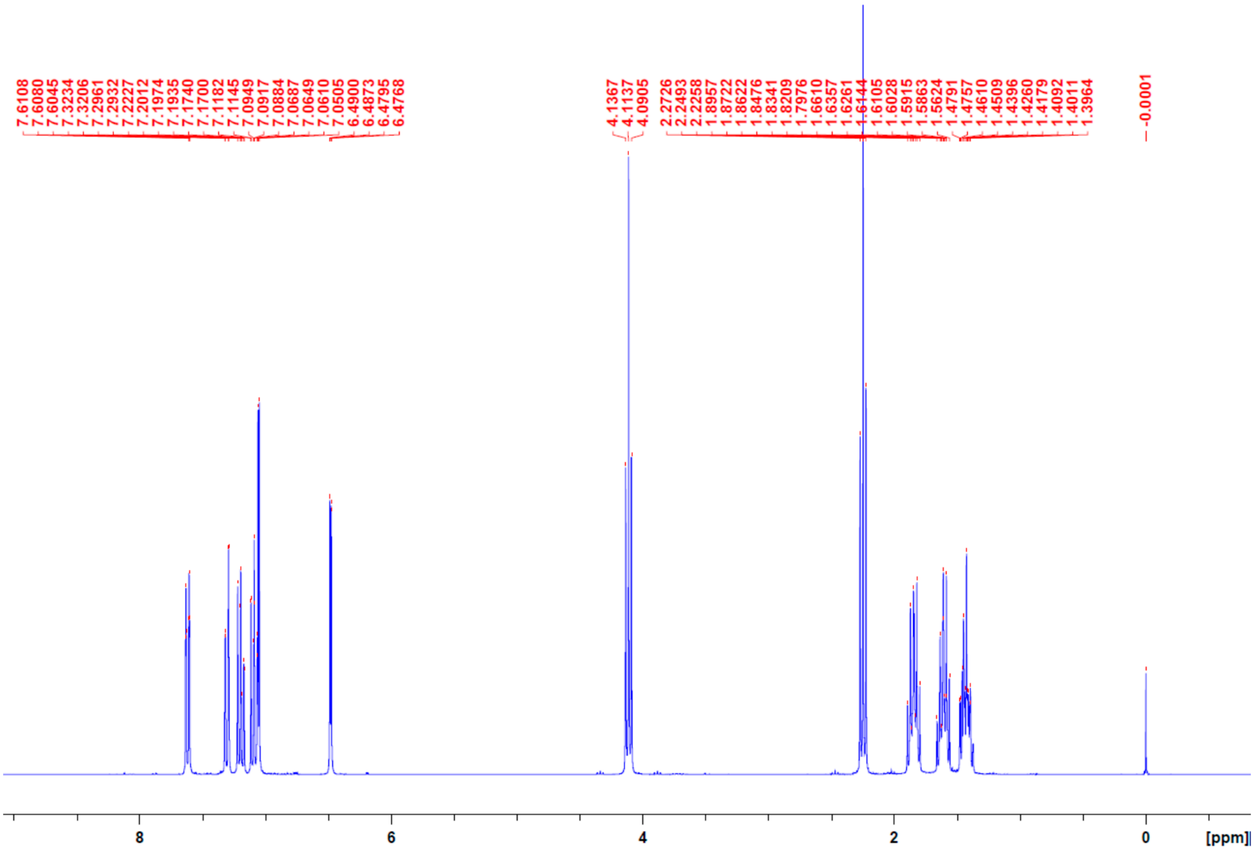
7.6108
7.6080
7.6045
7.3234
7.3206
7.2961
7.2932
7.2227
7.2012
7.1974
7.1935
7.1740
7.1700
7.1182
7.1145
7.0949
7.0917
7.0884
7.0687
7.0649
7.0610
7.0505
6.4900
6.4873
6.4795
6.4768
4.1367
4.1137
4.0905
2.2726
2.2493
2.2258
1.8957
1.8722
1.8622
1.8476
1.8341
1.8209
1.7976
1.6610
1.6357
1.6261
1.6144
1.6105
1.6028
1.5915
1.5863
1.5624
1.4791
1.4757
1.4610
1.4509
1.4396
1.4260
1.4179
1.4092
1.4011
1.3964
-0.0001
8
6
4
2
0
[ppm]

^{1}H NMR of **22**

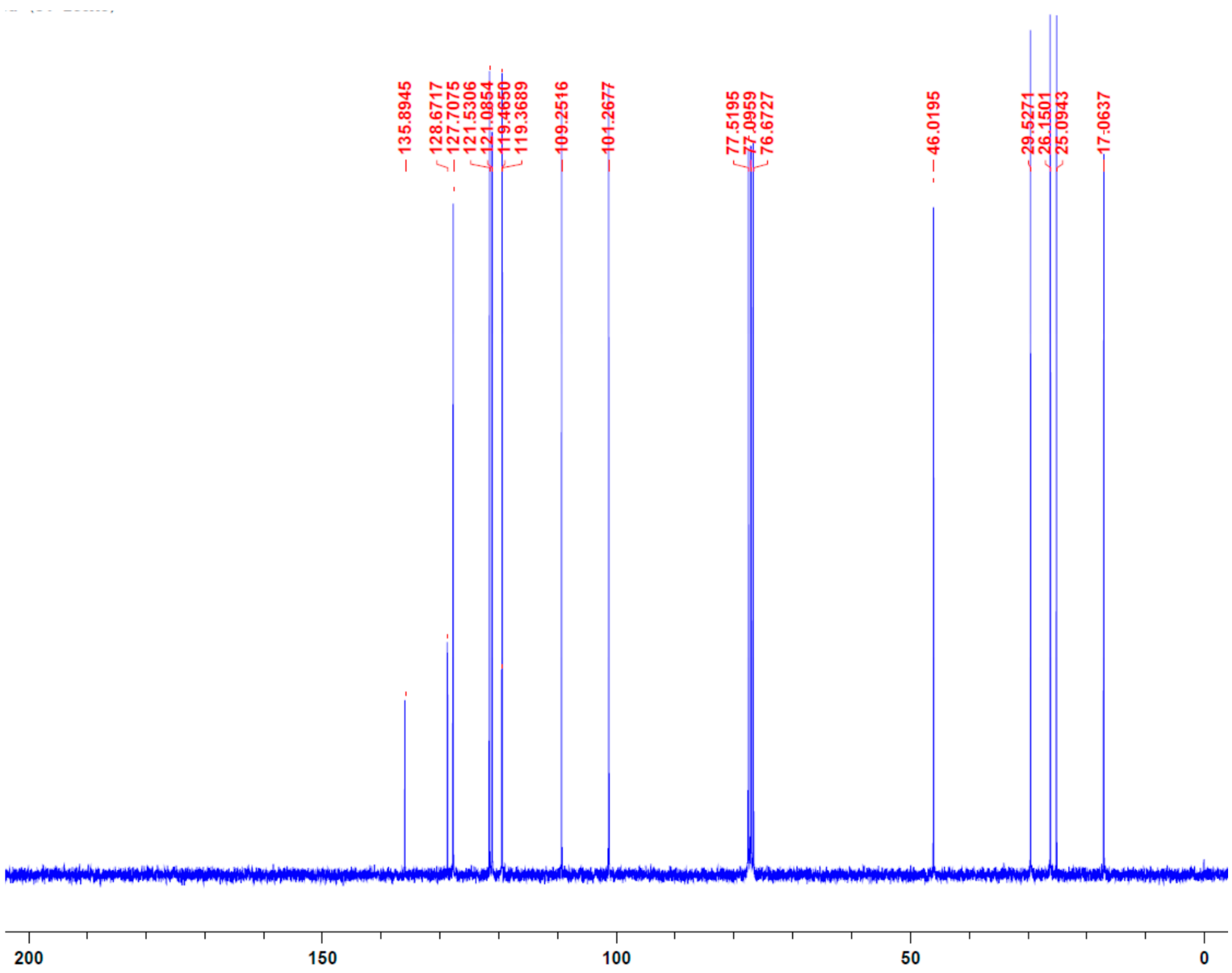

135.8945
128.6717
127.7075
121.5306
121.0854
119.4650
119.3689
109.2516
101.2677
77.5195
77.0959
76.6727
46.0195
29.5271
26.1501
25.0943
17.0637
200
150
100
50
0

^{13}C NMR of **22**

^{1}H NMR of **23**

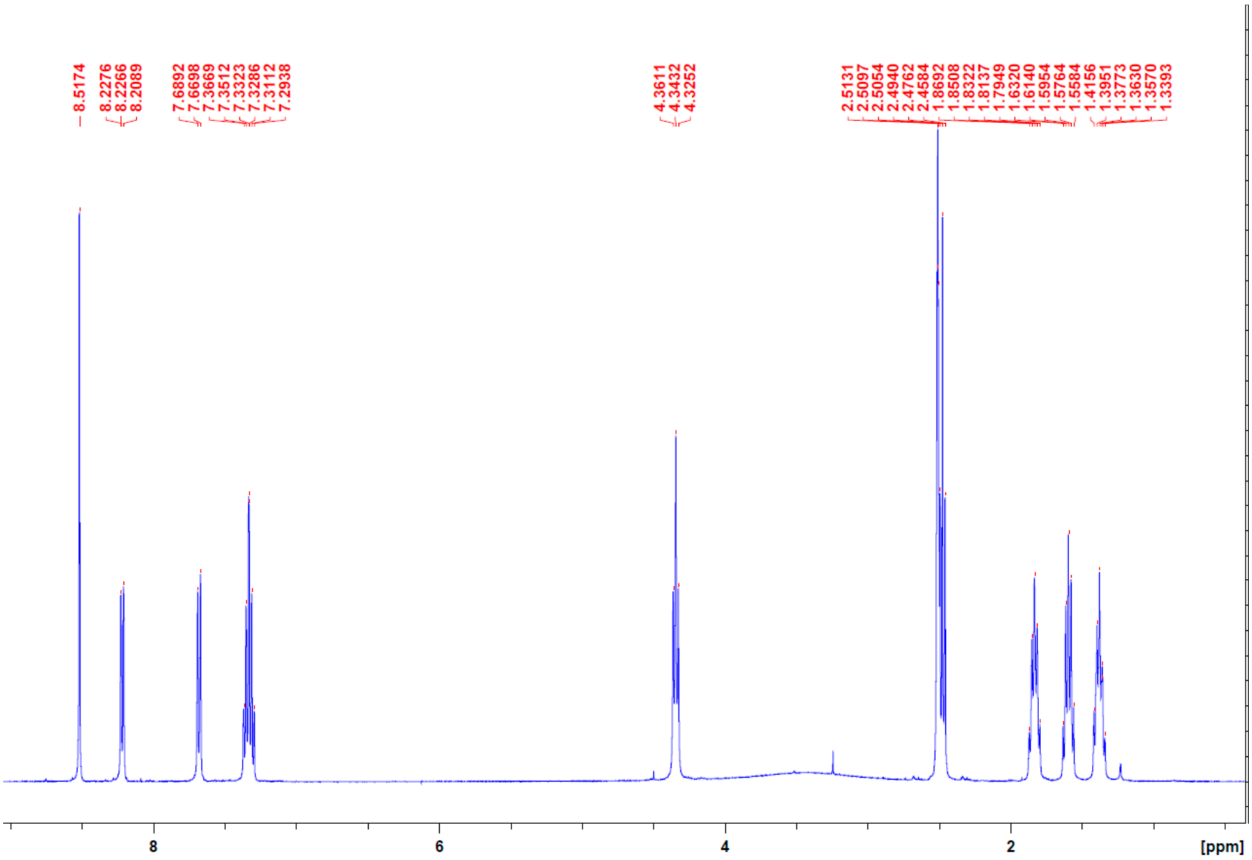
8.5174
8.2276
8.2266
8.2089
7.6892
7.6698
7.3669
7.3512
7.3323
7.3286
7.3112
7.2938
4.3611
4.3432
4.3252
2.5131
2.5097
2.5054
2.4940
2.4762
2.4584
1.8692
1.8508
1.8322
1.8137
1.7949
1.6320
1.6140
1.5954
1.5764
1.5584
1.4156
1.3951
1.3773
1.3630
1.3570
1.3393
8
6
4
2
[ppm]

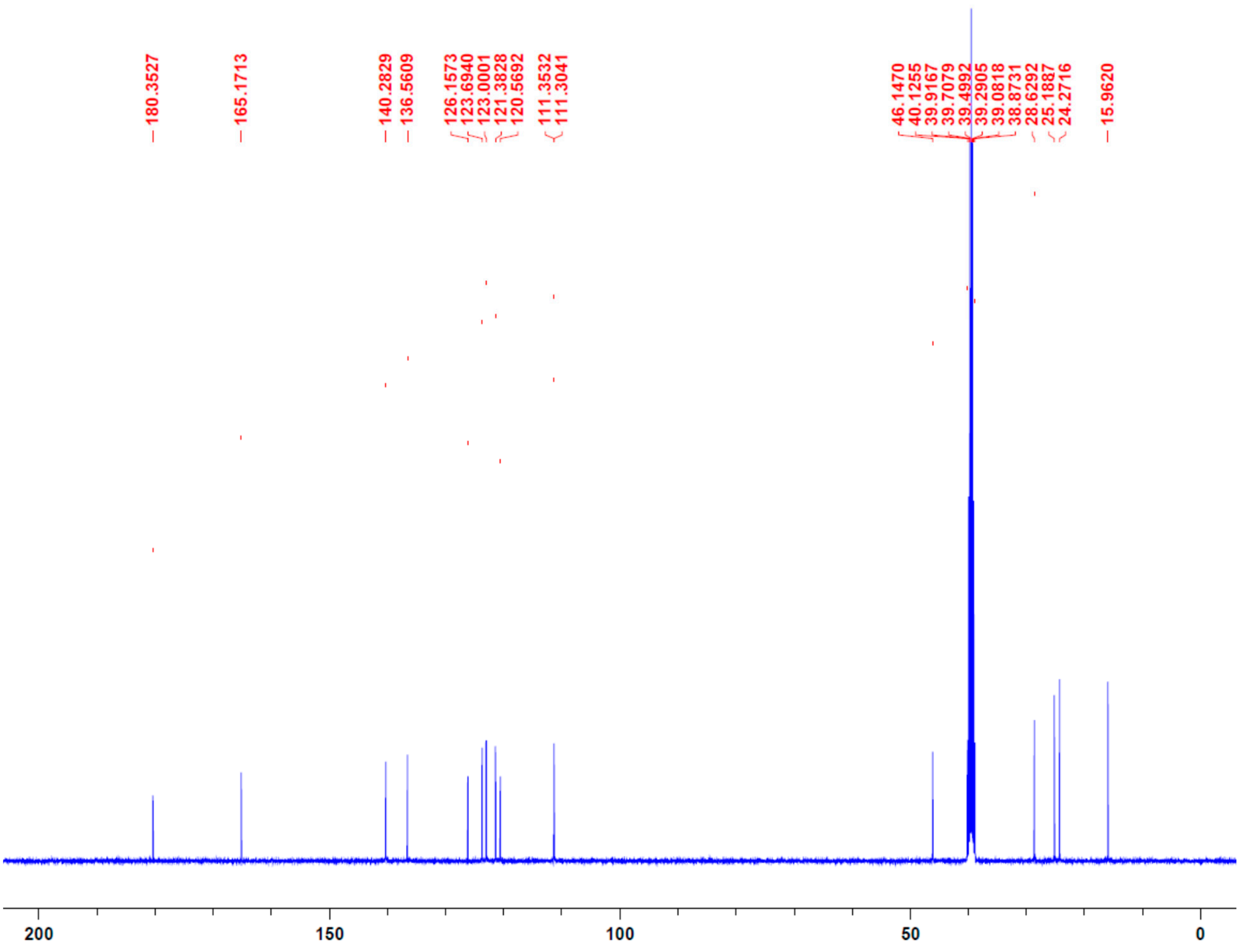
180.3527
165.1713
140.2829
136.5609
126.1573
123.6940
123.0001
121.3828
120.5692
111.3532
111.3041
46.1470
40.1255
39.9167
39.7079
39.4992
39.2905
39.0818
38.8731
28.6292
25.1887
24.2716
15.9620
200
150
100
50
0

^{13}C NMR of **23**

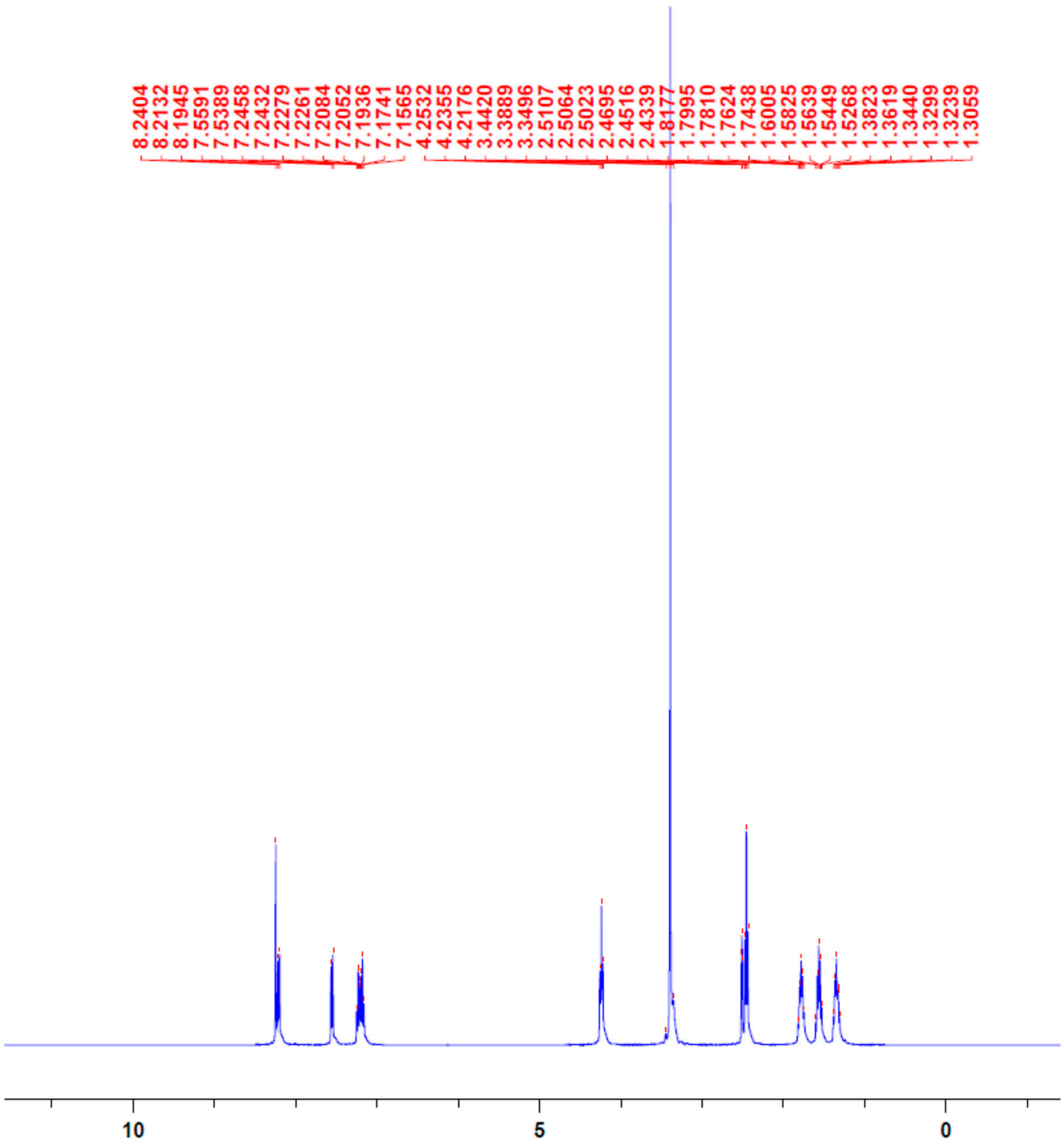
8.2404
8.2132
8.1945
7.5591
7.5389
7.2458
7.2432
7.2279
7.2261
7.2084
7.2052
7.1936
7.1741
7.1565
4.2532
4.2355
4.2176
3.4420
3.3889
3.3496
2.5107
2.5064
2.5023
2.4695
2.4516
2.4339
1.8177
1.7995
1.7810
1.7624
1.7438
1.6005
1.5825
1.5639
1.5449
1.5268
1.3823
1.3619
1.3440
1.3299
1.3239
1.3059
10
5
0

^{1}H NMR of **24**

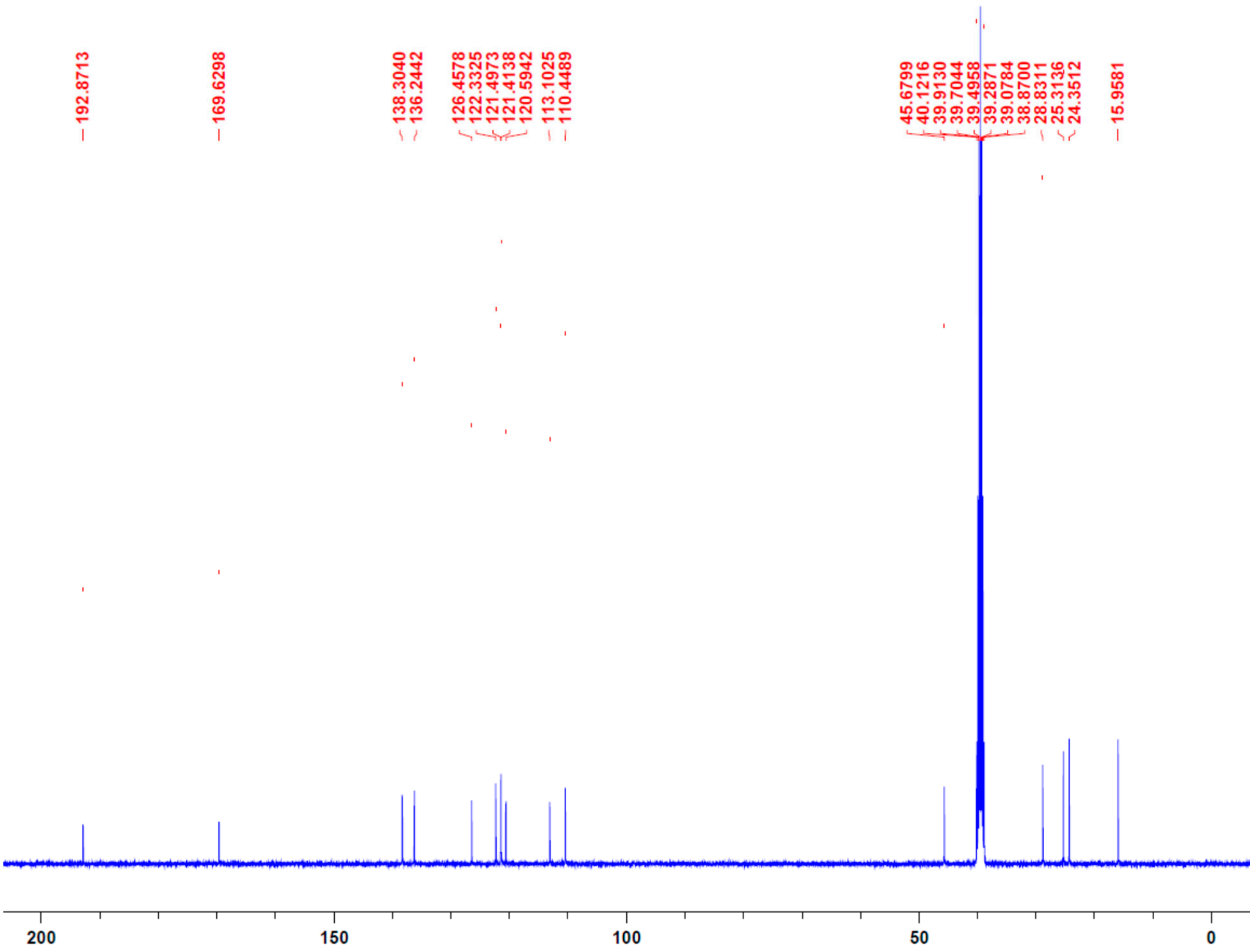
192.8713
169.6298
138.3040
136.2442
126.4578
122.3325
121.4973
121.4138
120.5942
113.1025
110.4489
45.6799
40.1216
39.9130
39.7044
39.4958
39.2871
39.0784
38.8700
28.8311
25.3136
24.3512
15.9581
200
150
100
50
0

^{13}C NMR of **24**

^{1}H NMR of **28**

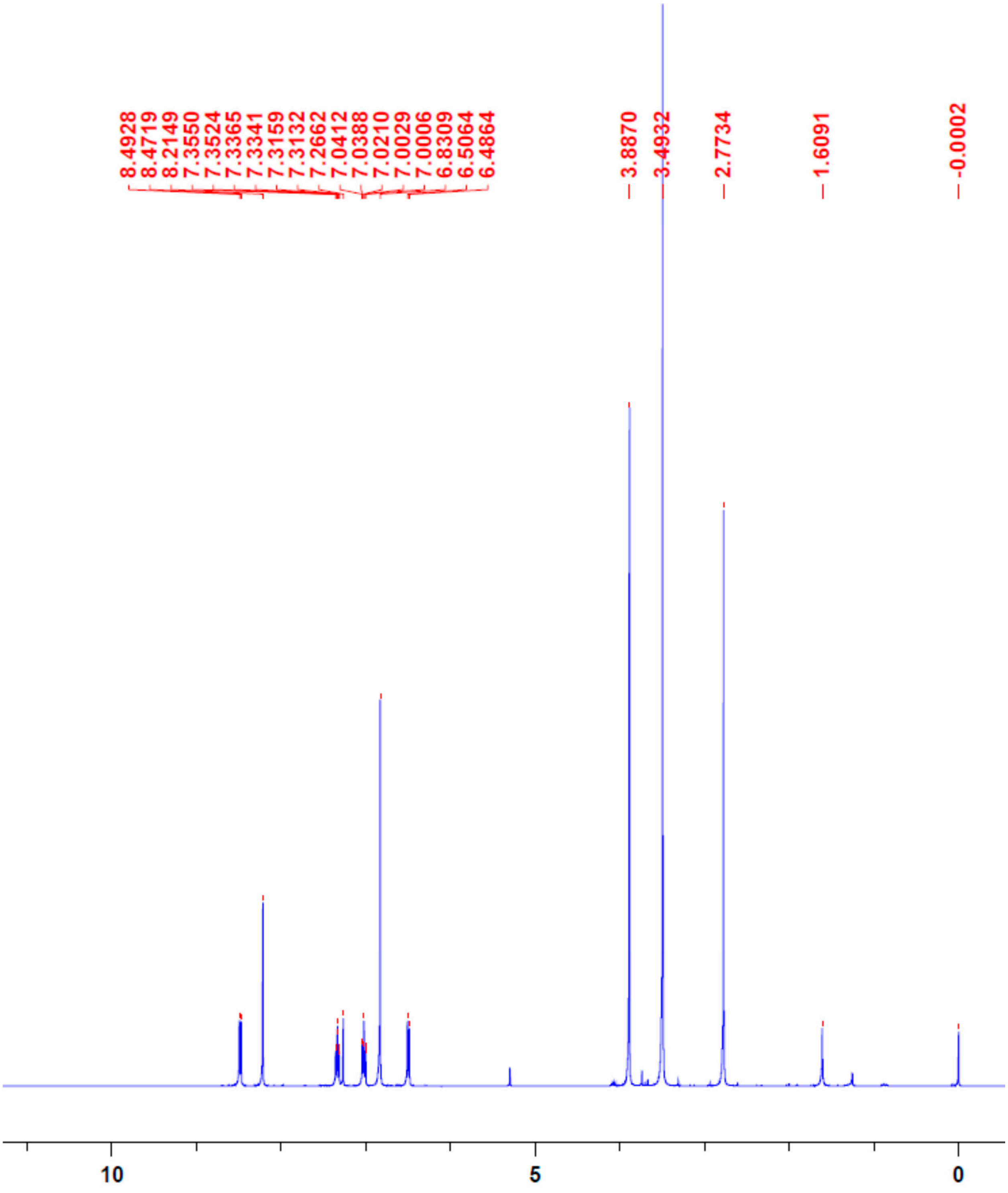
8.4928
8.4719
8.2149
7.3550
7.3524
7.3365
7.3341
7.3159
7.3132
7.2662
7.0412
7.0388
7.0210
7.0029
7.0006
6.8309
6.5064
6.4864
3.8870
3.4932
2.7734
1.6091
-0.0002
10
5
0

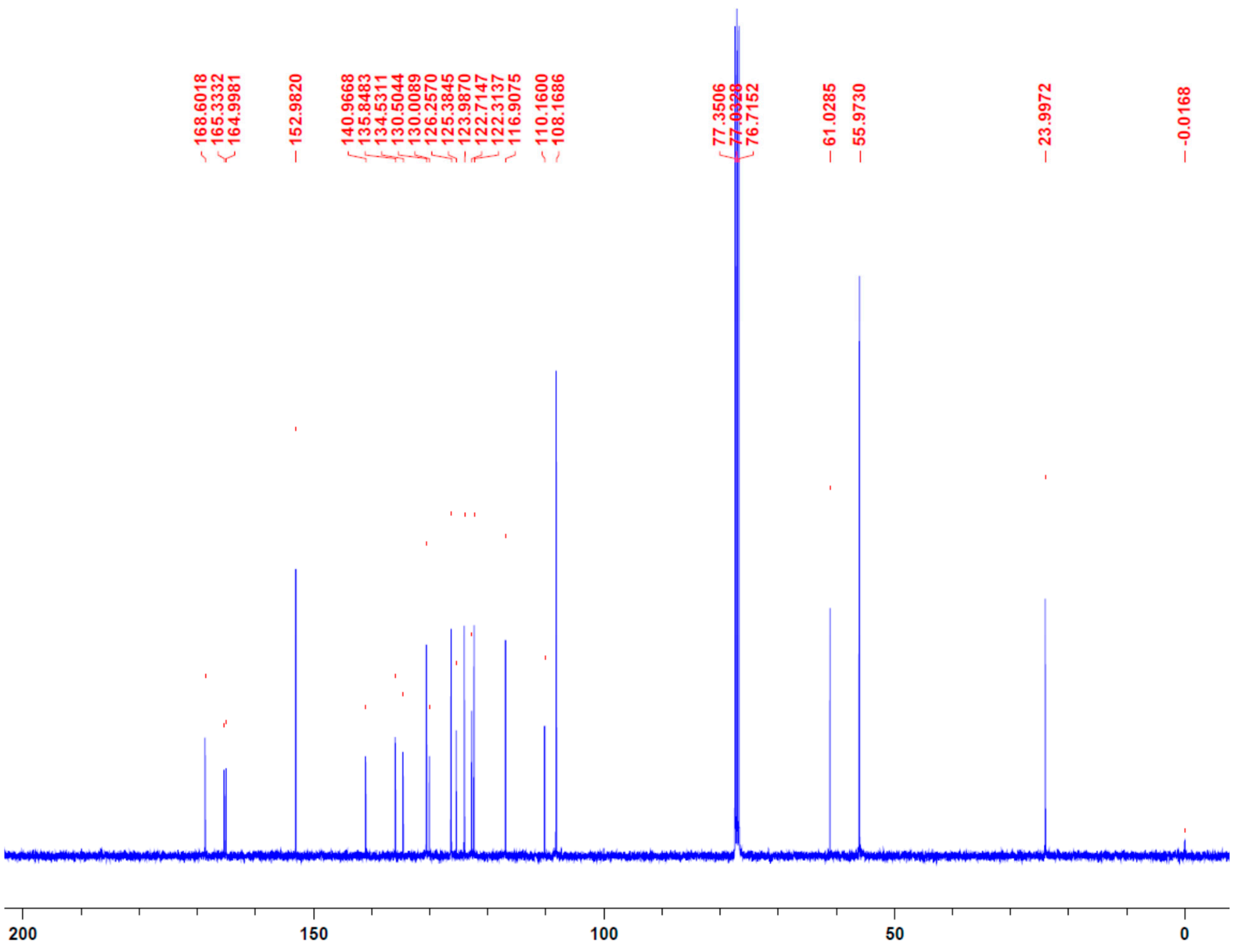
168.6018
165.3332
164.9981
152.9820
140.9668
135.8483
134.5311
130.5044
130.0089
126.2570
125.3845
123.9870
122.7147
122.3137
116.9075
110.1600
108.1686
77.3506
77.0328
76.7152
61.0285
55.9730
23.9972
-0.0168
200
150
100
50
0

^{13}C NMR of **28**

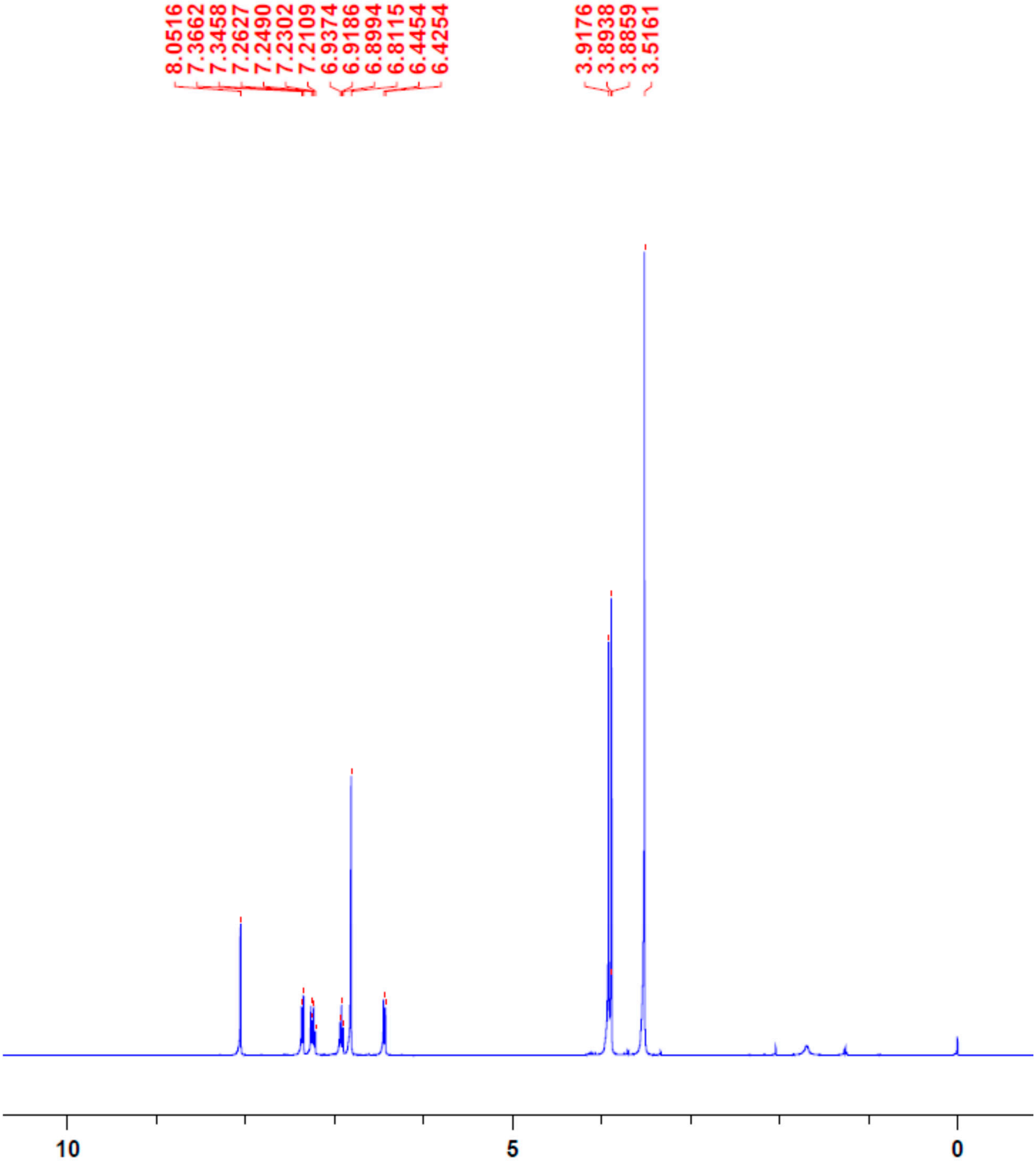
8.0516
7.3662
7.3458
7.2627
7.2490
7.2302
7.2109
6.9374
6.9186
6.8994
6.8115
6.4454
6.4254
3.9176
3.8938
3.8859
3.5161
10
5
0

^{1}H NMR of **29**

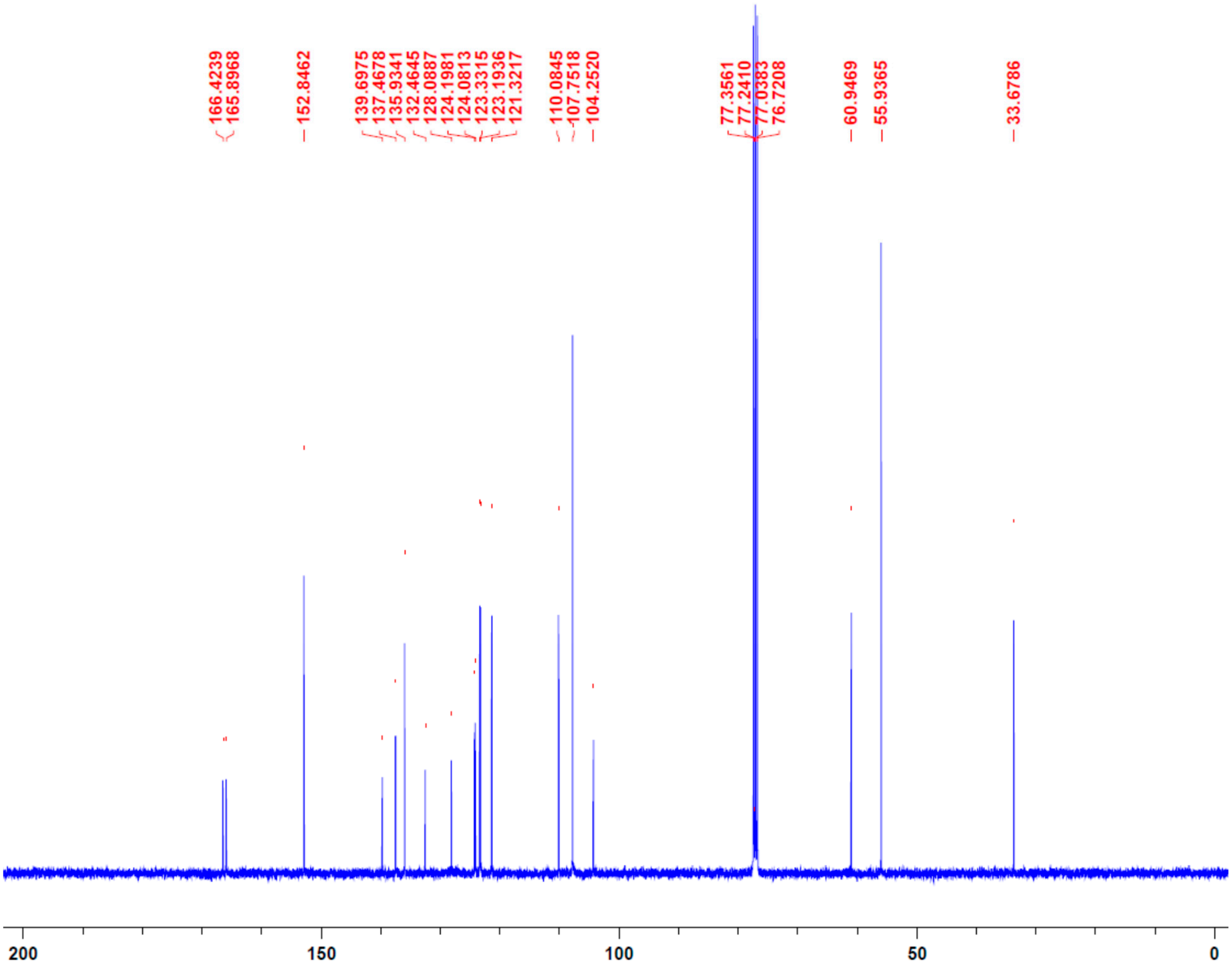
166.4239
165.8968
152.8462
139.6975
137.4678
135.9341
132.4645
128.0887
124.1981
124.0813
123.3315
123.1936
121.3217
110.0845
107.7518
104.2520
77.3561
77.2410
77.0383
76.7208
60.9469
55.9365
33.6786
200
150
100
50
0

^{13}C NMR of **29**

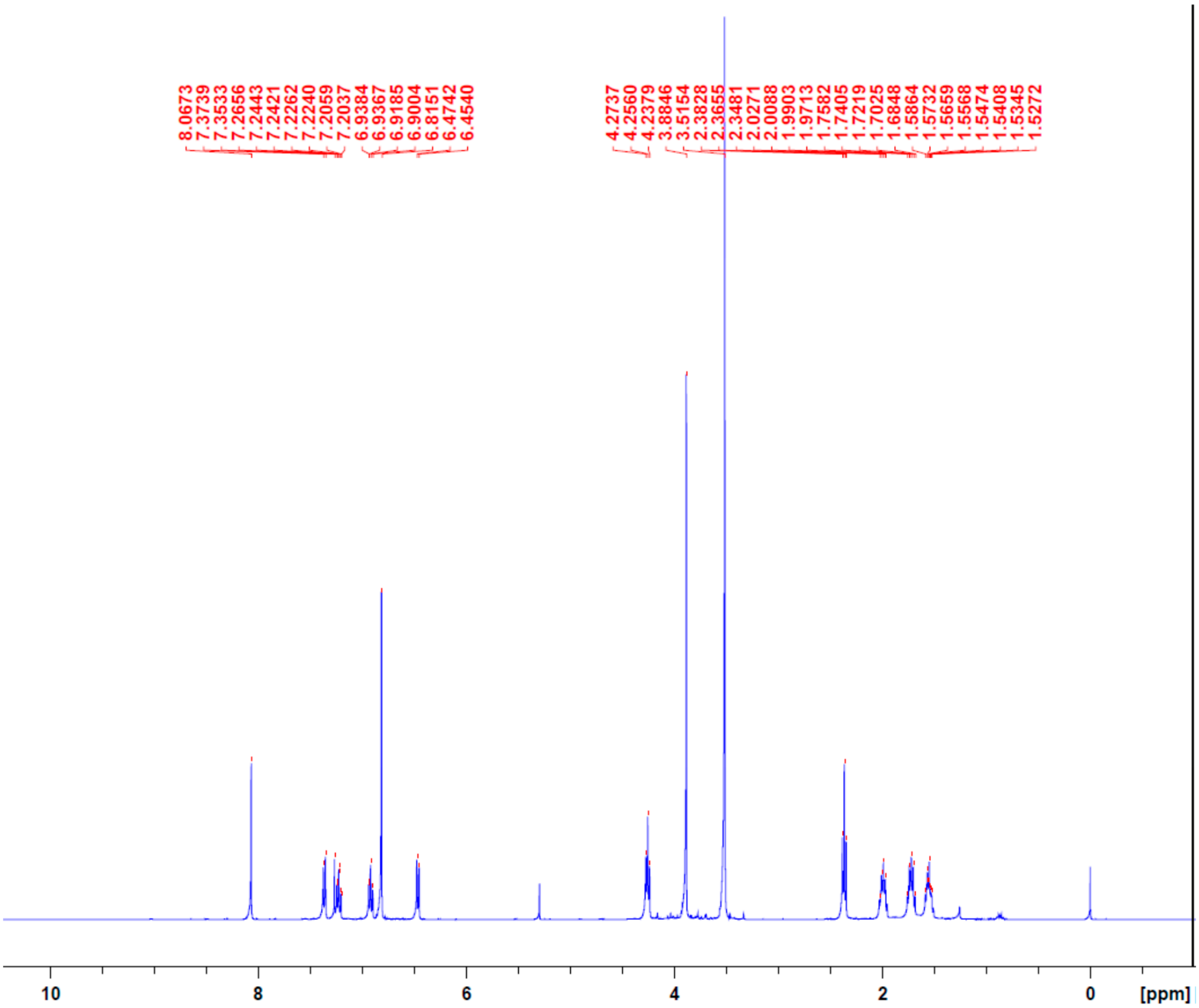
8.0673
7.3739
7.3533
7.2656
7.2443
7.2421
7.2262
7.2240
7.2059
7.2037
6.9384
6.9367
6.9185
6.9004
6.8151
6.4742
6.4540
4.2737
4.2560
4.2379
3.8846
3.5154
2.3828
2.3655
2.3481
2.0271
2.0088
1.9903
1.9713
1.7582
1.7405
1.7219
1.7025
1.6848
1.5864
1.5732
1.5659
1.5568
1.5474
1.5408
1.5345
1.5272
10
8
6
4
2
0
[ppm]

^{1}H NMR of **30**

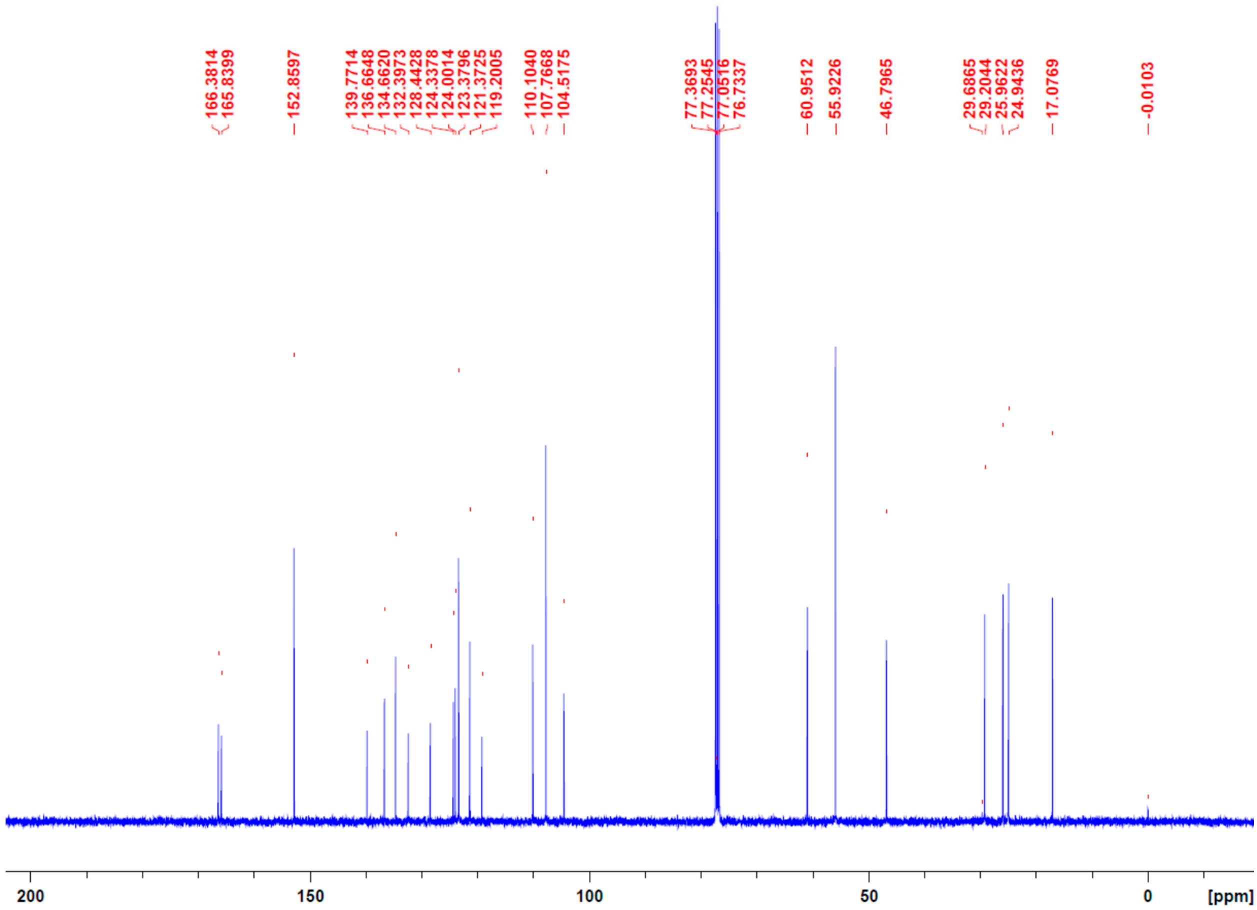
166.3814
165.8399
152.8597
139.7714
136.6648
134.6620
132.3973
128.4428
124.3378
124.0014
123.3796
121.3725
119.2005
110.1040
107.7668
104.5175
77.3693
77.2545
77.0516
76.7337
60.9512
55.9226
46.7965
29.6865
29.2044
25.9622
24.9436
17.0769
-0.0103
200
150
100
50
0
[ppm]

^{13}C NMR of **30**

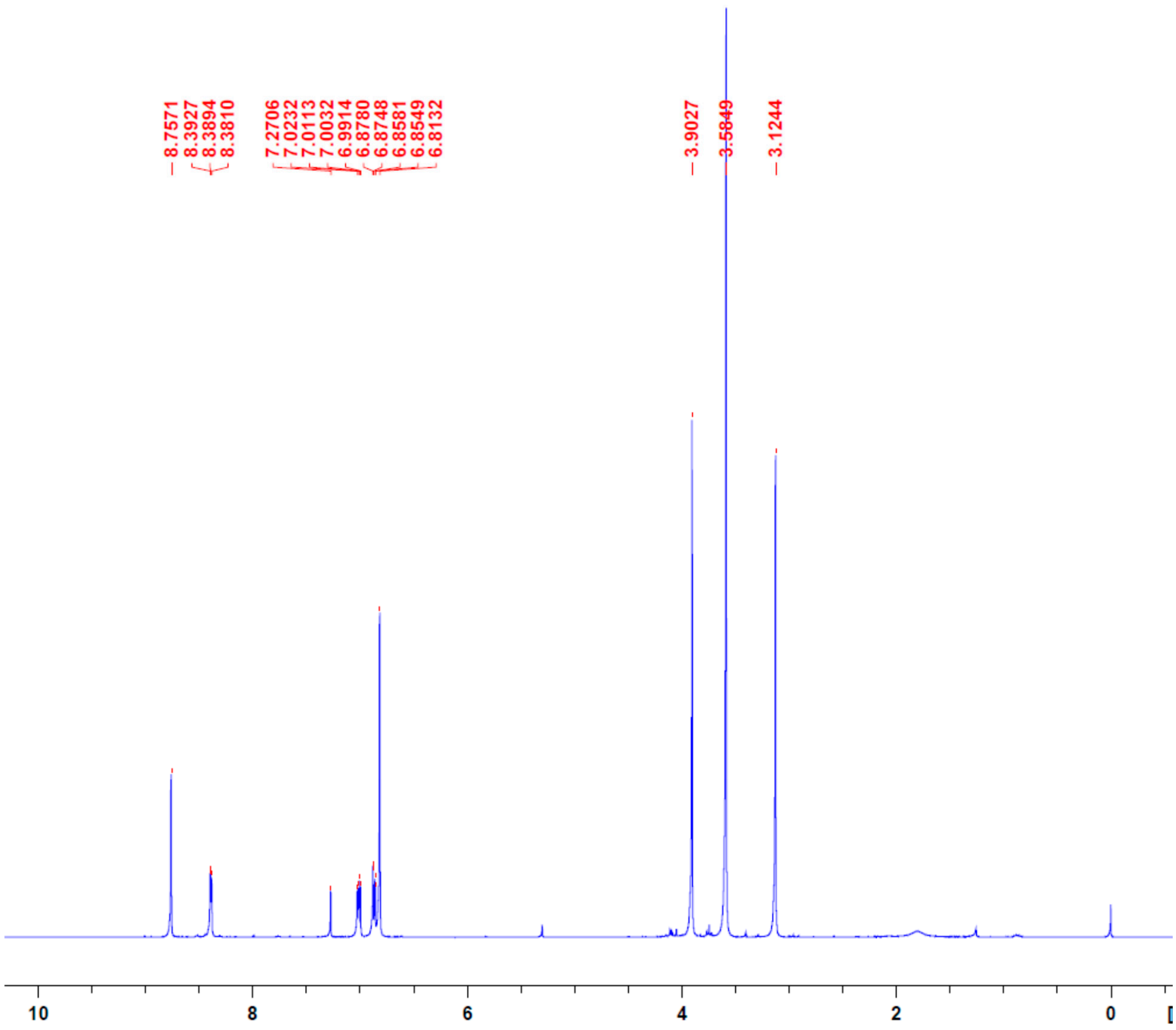
8.7571
8.3927
8.3894
8.3810
7.2706
7.0232
7.0113
7.0032
6.9914
6.8780
6.8748
6.8581
6.8549
6.8132
3.9027
3.5849
3.1244
10
8
6
4
2
0

^{1}H NMR of **31**

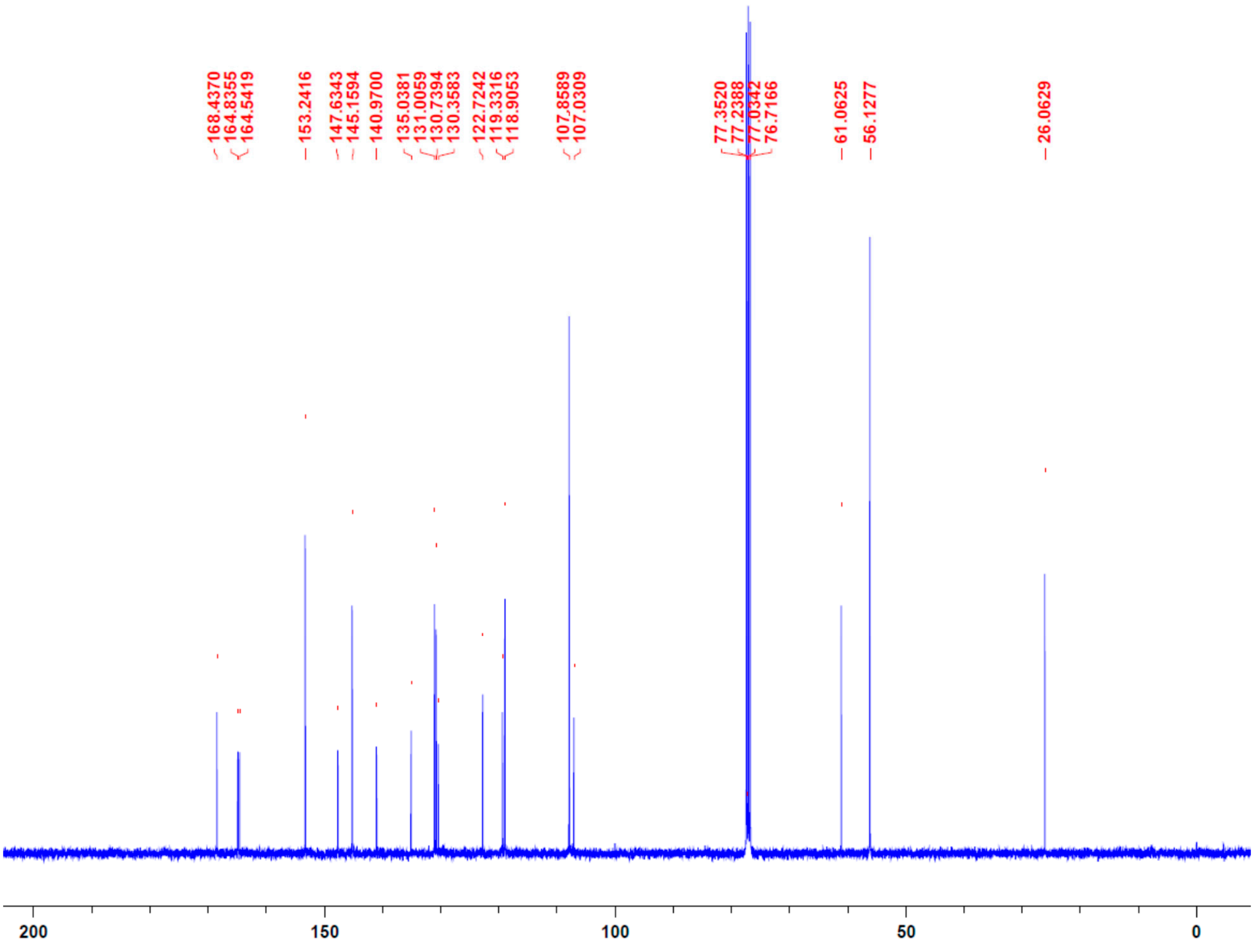
168.4370
164.8355
164.5419
153.2416
147.6343
145.1594
140.9700
135.0381
131.0059
130.7394
130.3583
122.7242
119.3316
118.9053
107.8589
107.0309
77.3520
77.2388
77.0342
76.7166
61.0625
56.1277
26.0629
200
150
100
50
0

^{13}C NMR of **31**

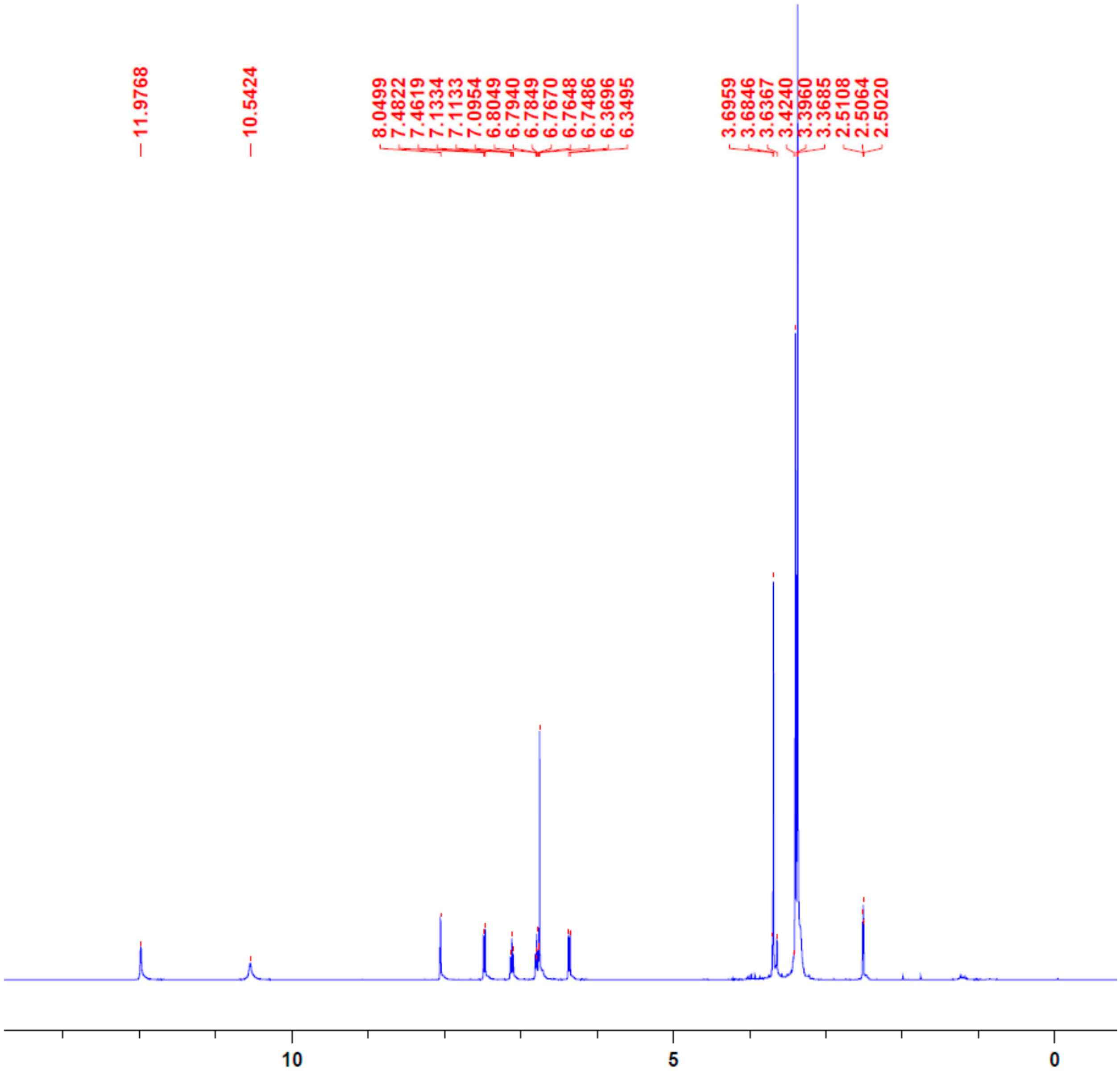
11.9768
10.5424
8.0499
7.4822
7.4619
7.1334
7.1133
7.0954
6.8049
6.7940
6.7849
6.7670
6.7648
6.7486
6.3696
6.3495
3.6959
3.6846
3.6367
3.4240
3.3960
3.3685
2.5108
2.5064
2.5020
10
5
0

^{1}H NMR of **32**

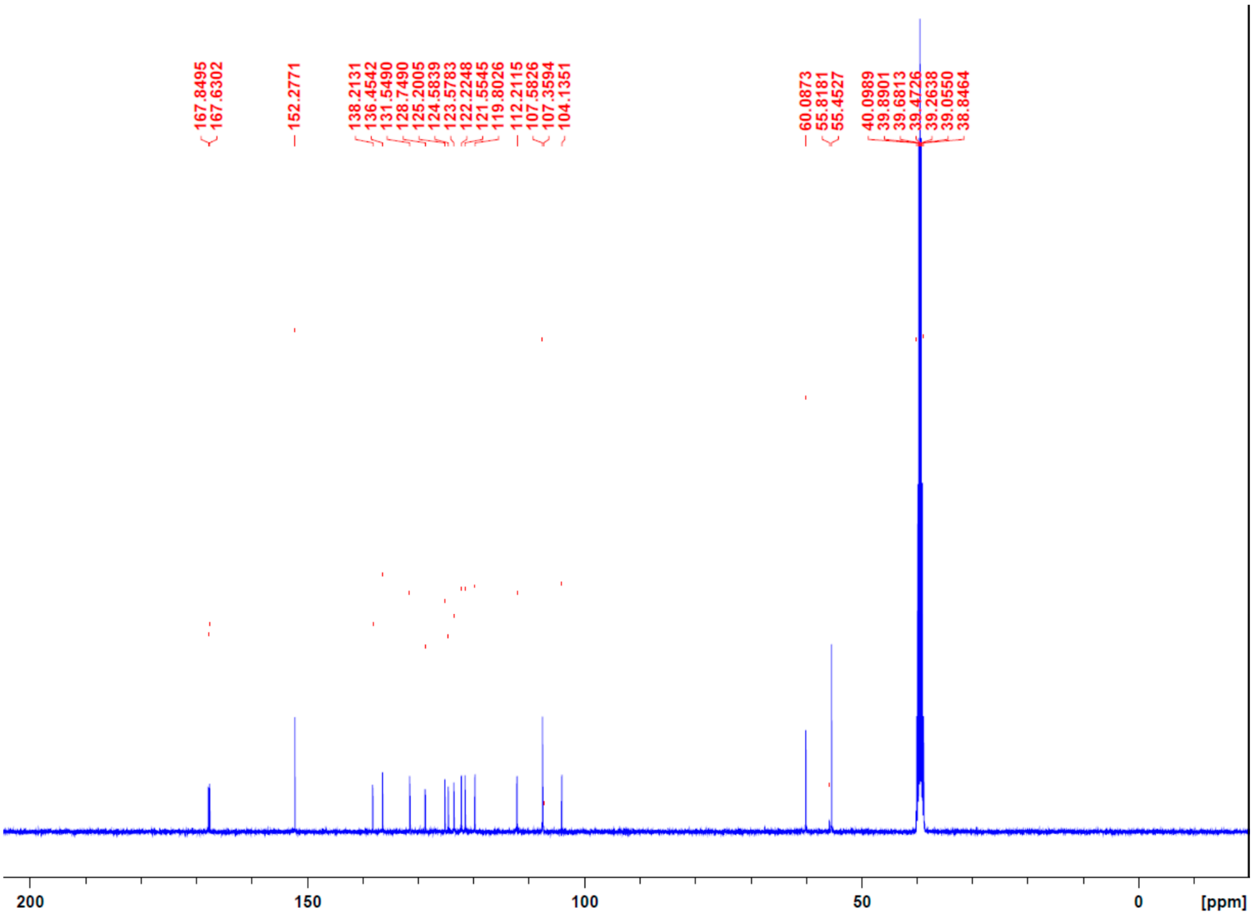
167.8495
167.6302
152.2771
138.2131
136.4542
131.5490
128.7490
125.2005
124.5839
123.5783
122.2248
121.5545
119.8026
112.2115
107.5826
107.3594
104.1351
60.0873
55.8181
55.4527
40.0989
39.8901
39.6813
39.4726
39.2638
39.0550
38.8464
200
150
100
50
0
[ppm]

^{13}C NMR of **32**

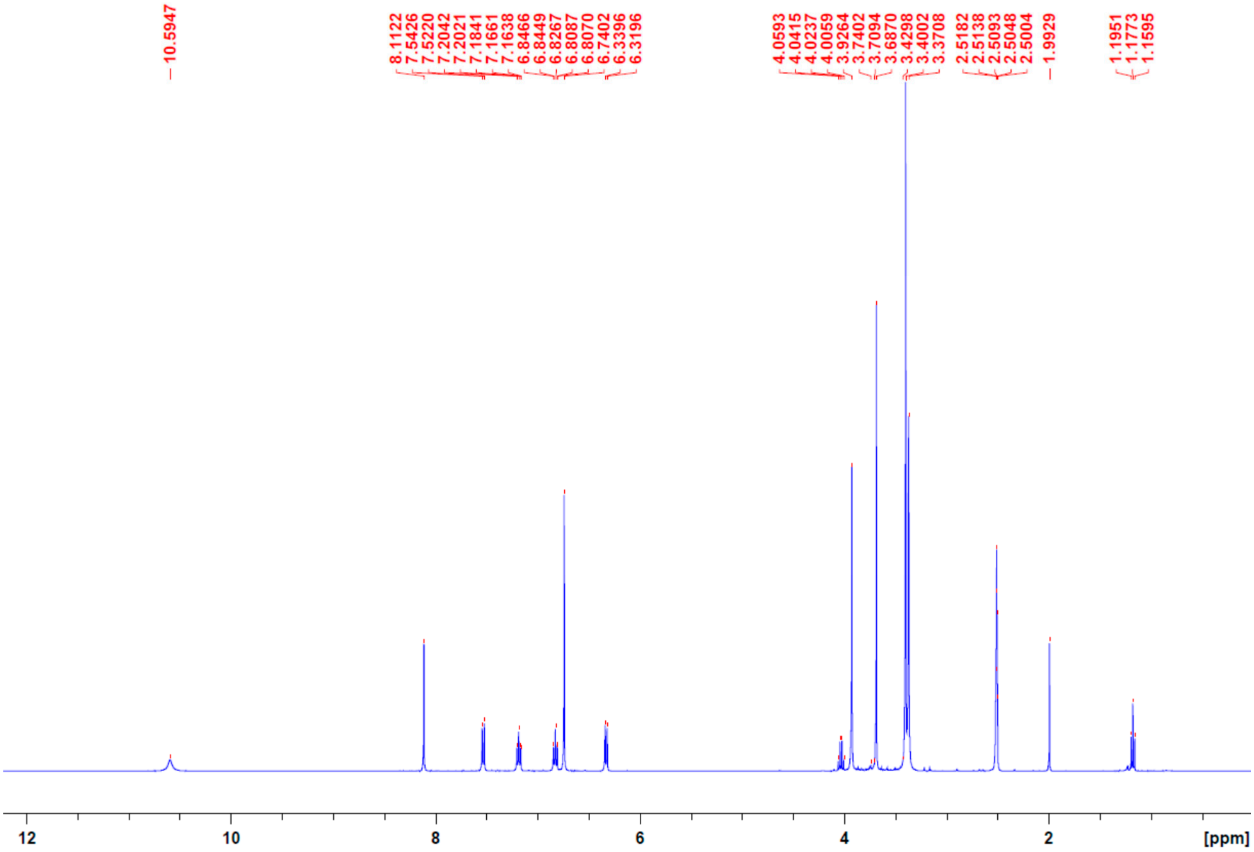
10.5947
8.1122
7.5426
7.5220
7.2042
7.2021
7.1841
7.1661
7.1638
6.8466
6.8449
6.8267
6.8087
6.8070
6.7402
6.3396
6.3196
4.0593
4.0415
4.0237
4.0059
3.9264
3.7402
3.7094
3.6870
3.4298
3.4002
3.3708
2.5182
2.5138
2.5093
2.5048
2.5004
1.9929
1.1951
1.1773
1.1595
12
10
8
6
4
2
[ppm]

¹H NMR of **33**

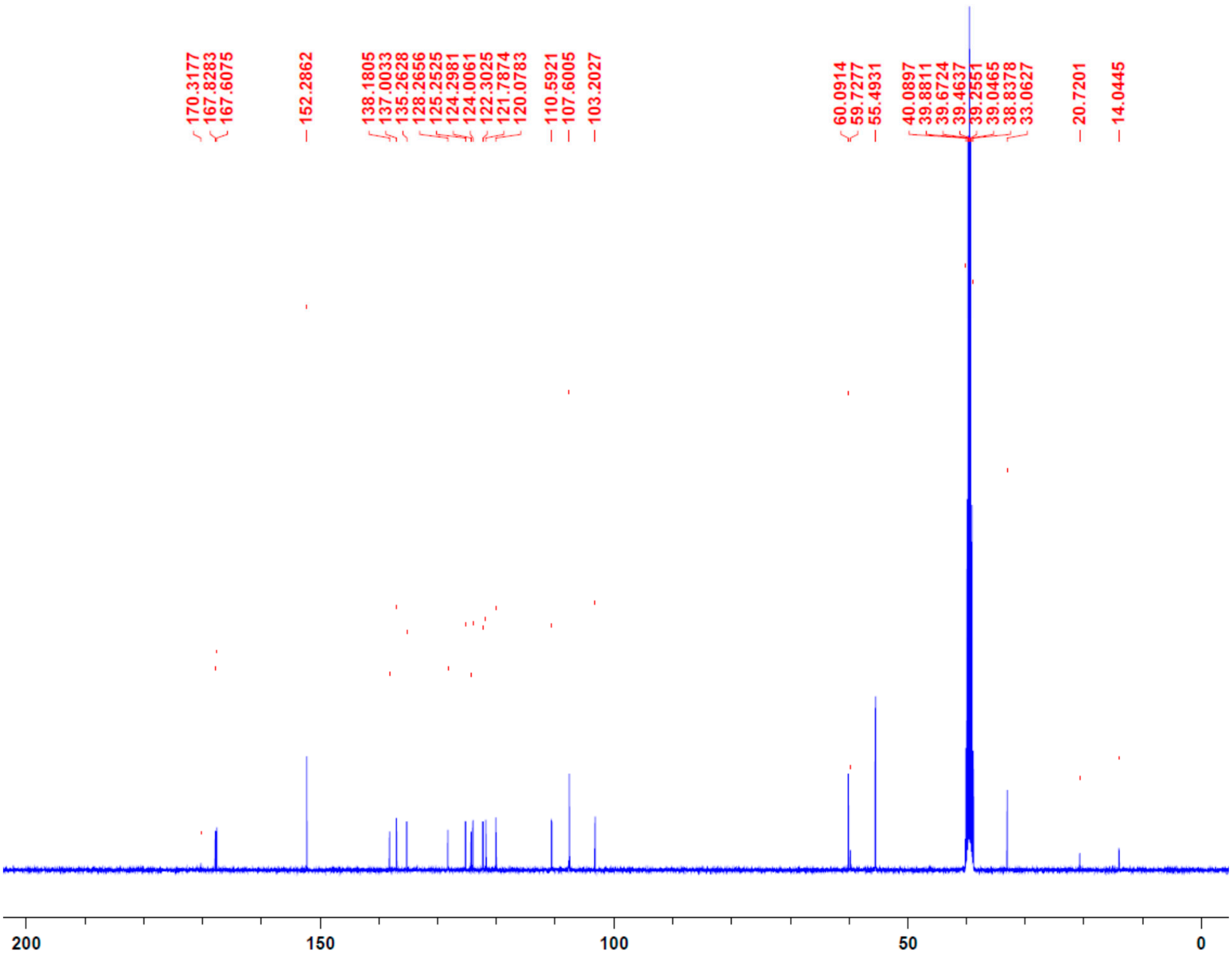
170.3177
167.8283
167.6075
152.2862
138.1805
137.0033
135.2628
128.2656
125.2525
124.2981
124.0061
122.3025
121.7874
120.0783
110.5921
107.6005
103.2027
60.0914
59.7277
55.4931
40.0897
39.8811
39.6724
39.4637
39.2551
39.0465
38.8378
33.0627
20.7201
14.0445
200
150
100
50
0

^{13}C NMR of **33**

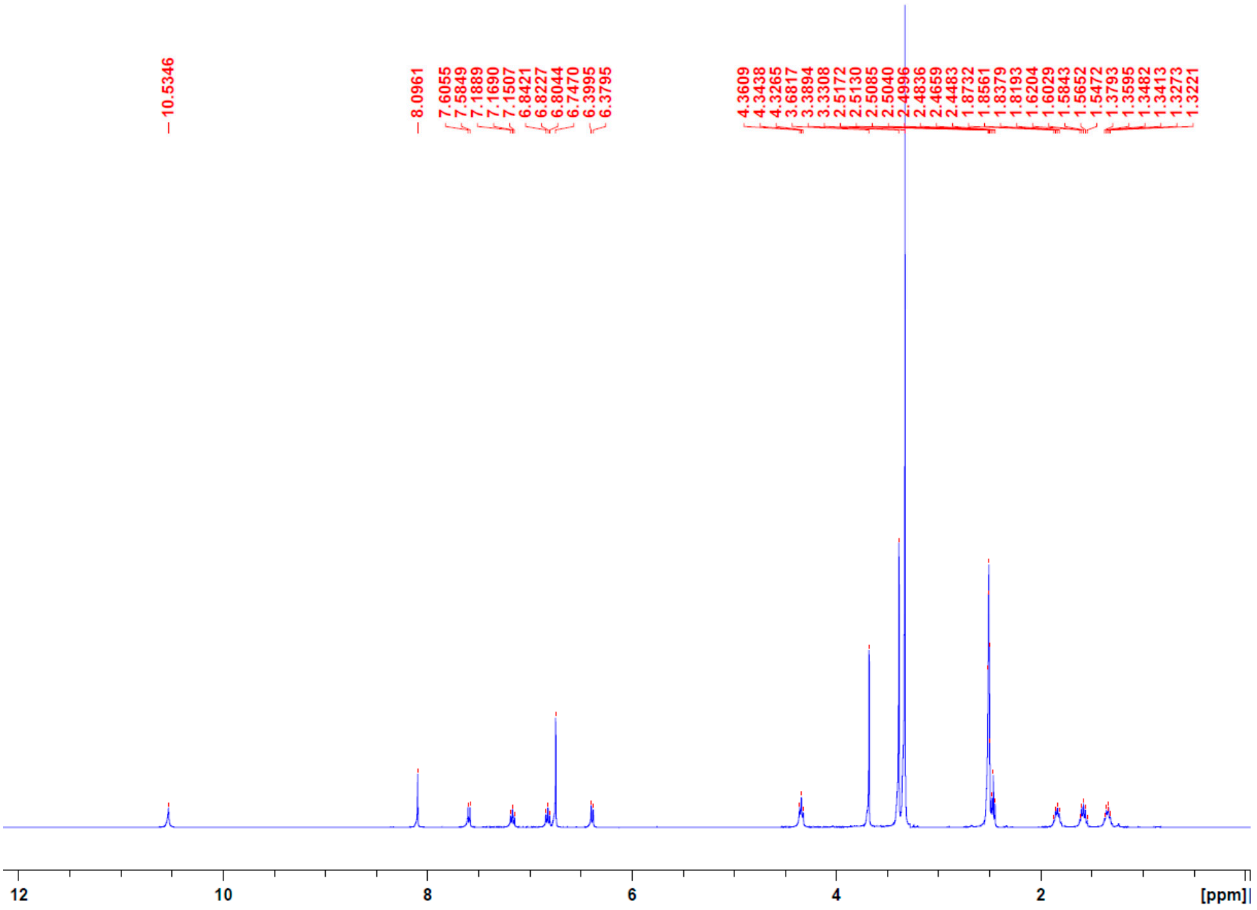
10.5346
8.0961
7.6055
7.5849
7.1889
7.1690
7.1507
6.8421
6.8227
6.8044
6.7470
6.3995
6.3795
4.3609
4.3438
4.3265
3.6817
3.3894
3.3308
2.5172
2.5130
2.5085
2.5040
2.4996
2.4836
2.4659
2.4483
1.8732
1.8561
1.8379
1.8193
1.6204
1.6029
1.5843
1.5652
1.5472
1.3793
1.3595
1.3482
1.3413
1.3273
1.3221
12
10
8
6
4
2
[ppm]

^{1}H NMR of **34**

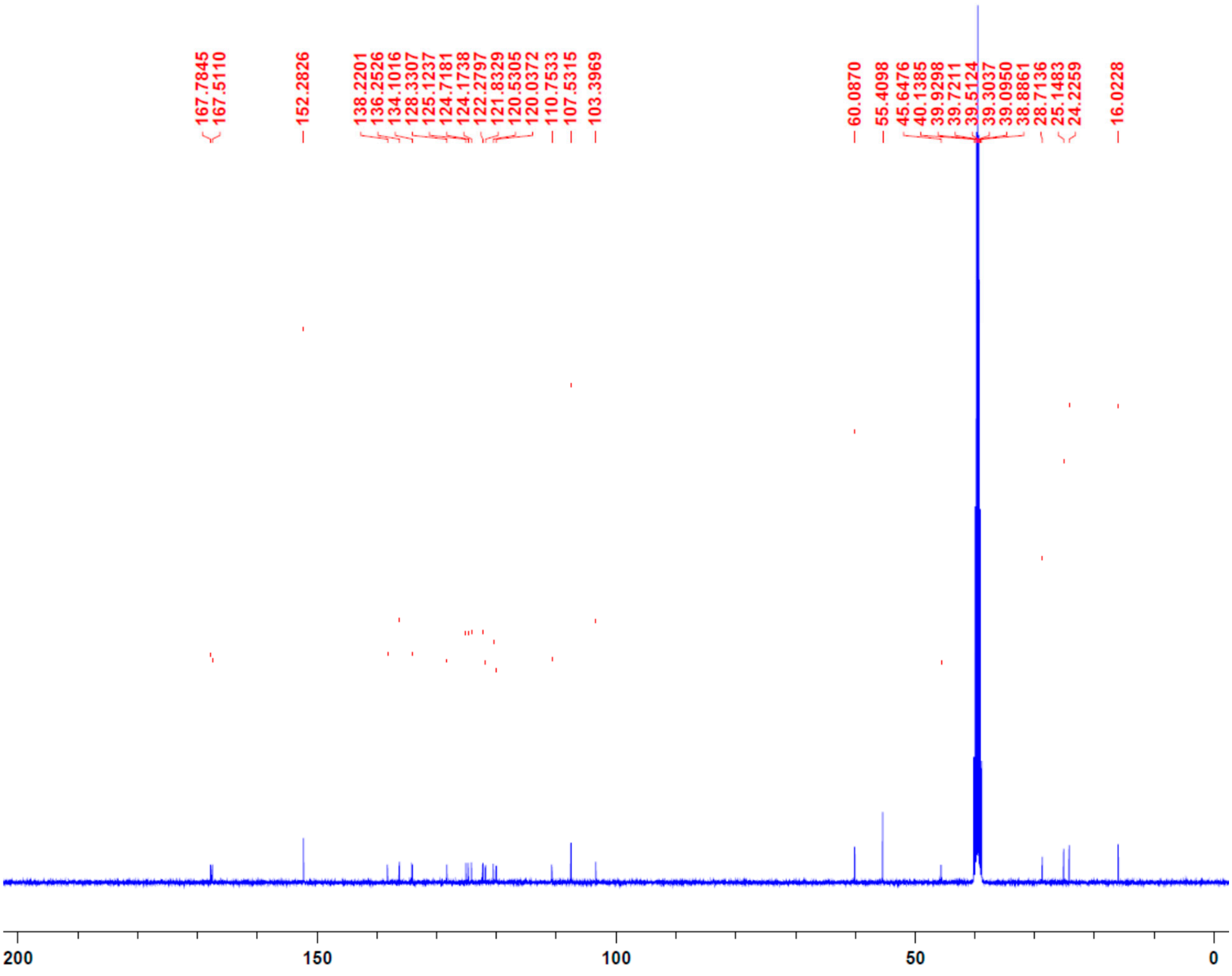
167.7845
167.5110
152.2826
138.2201
136.2526
134.1016
128.3307
125.1237
124.7181
124.1738
122.2797
121.8329
120.5305
120.0372
110.7533
107.5315
103.3969
60.0870
55.4098
45.6476
40.1385
39.9298
39.7211
39.5124
39.3037
39.0950
38.8861
28.7136
25.1483
24.2259
16.0228
200
150
100
50
0

^{13}C NMR of **34**

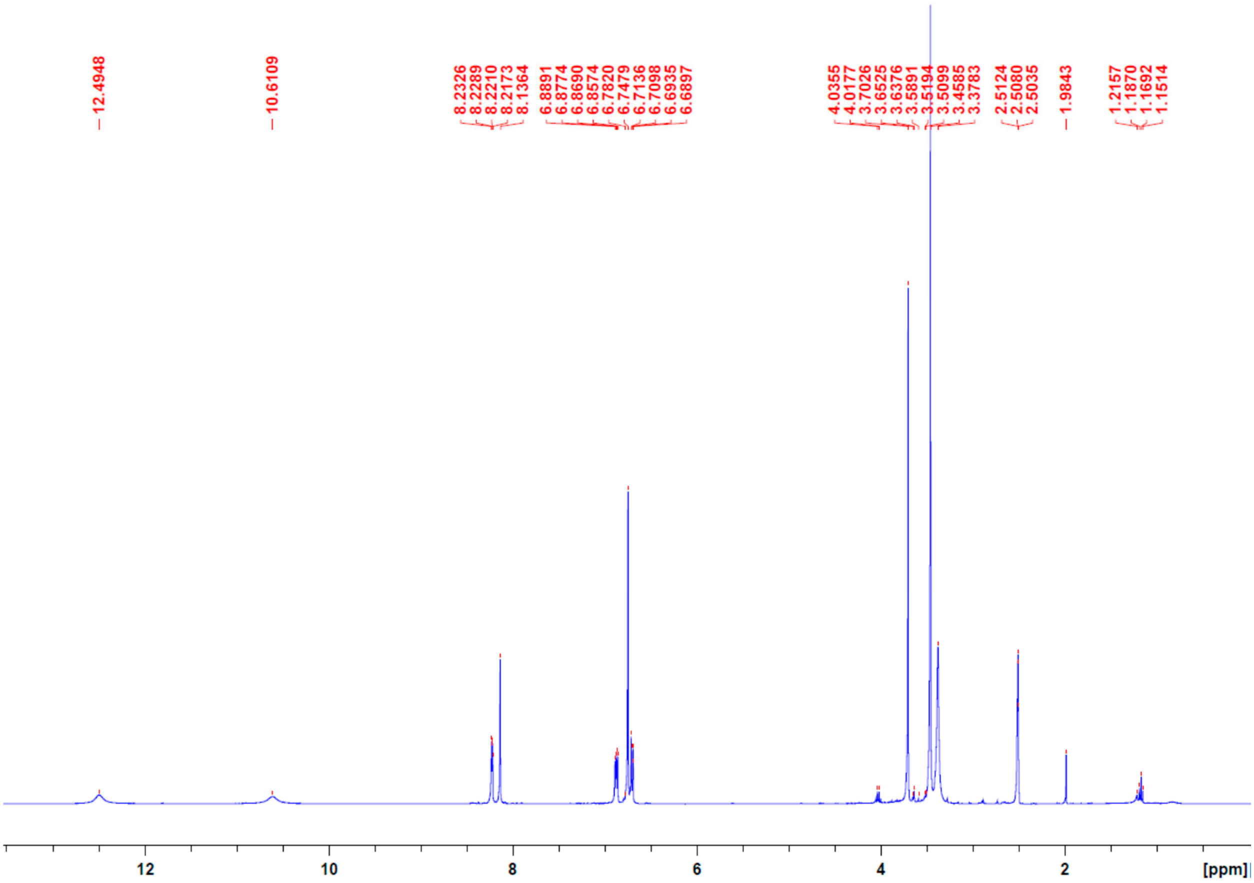
12.4948
10.6109
8.2326
8.2289
8.2210
8.2173
8.1364
6.8891
6.8774
6.8690
6.8574
6.7820
6.7479
6.7136
6.7098
6.6935
6.6897
4.0355
4.0177
3.7026
3.6525
3.6376
3.5891
3.5194
3.5099
3.4585
3.3783
2.5124
2.5080
2.5035
1.9843
1.2157
1.1870
1.1692
1.1514
12
10
8
6
4
2
[ppm]

^{1}H NMR of **35**

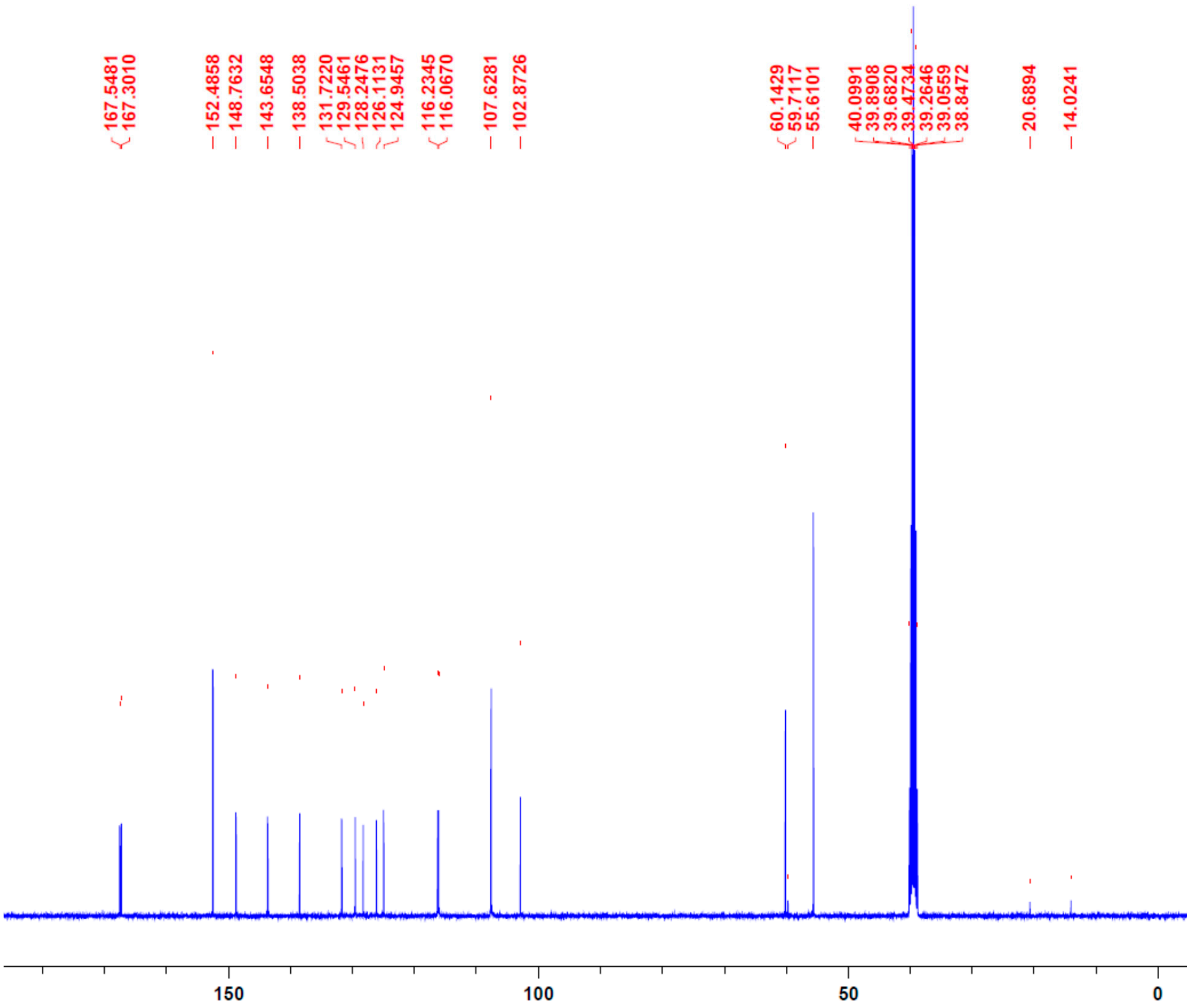
167.5481
167.3010
152.4858
148.7632
143.6548
138.5038
131.7220
129.5461
128.2476
126.1131
124.9457
116.2345
116.0670
107.6281
102.8726
60.1429
59.7117
55.6101
40.0991
39.8908
39.6820
39.4734
39.2646
39.0559
38.8472
20.6894
14.0241
150
100
50
0

^{13}C NMR of **35**

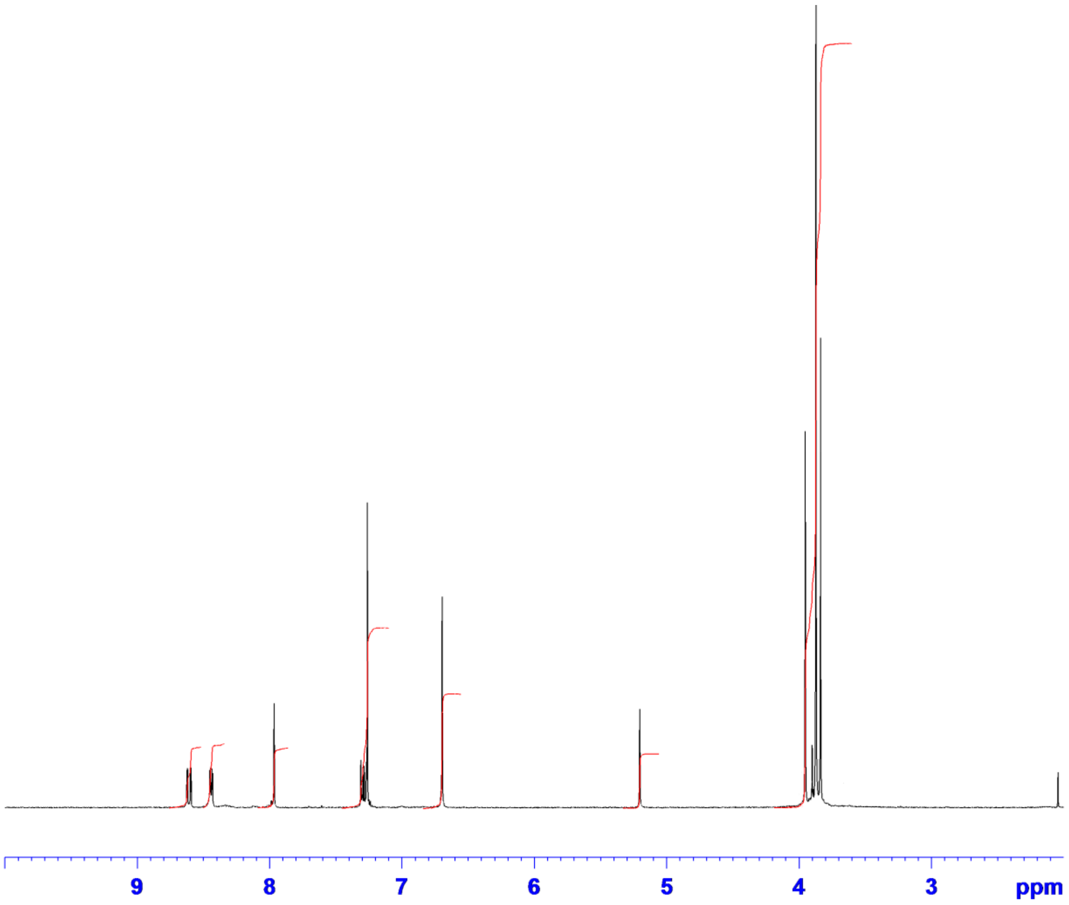

^{1}H NMR of β-ketonitrile **39**

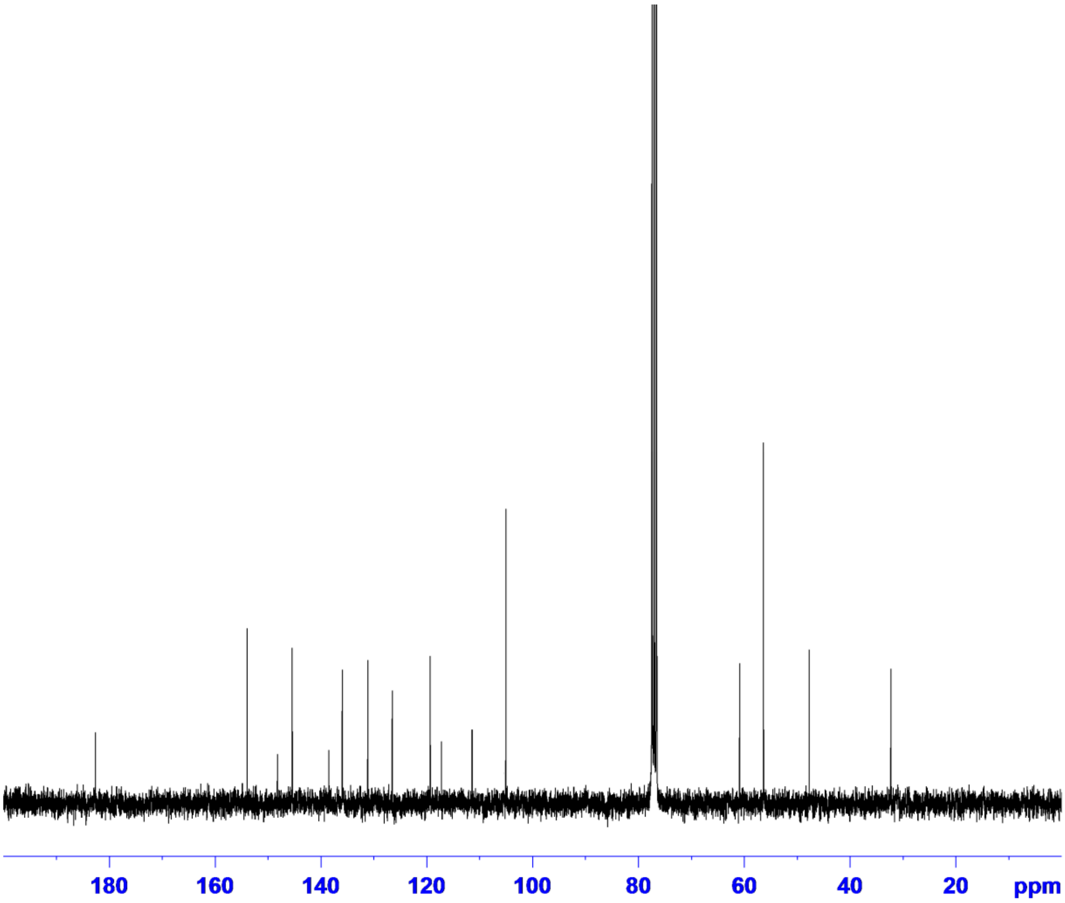

^{13}C NMR of β-ketonitrile **39**

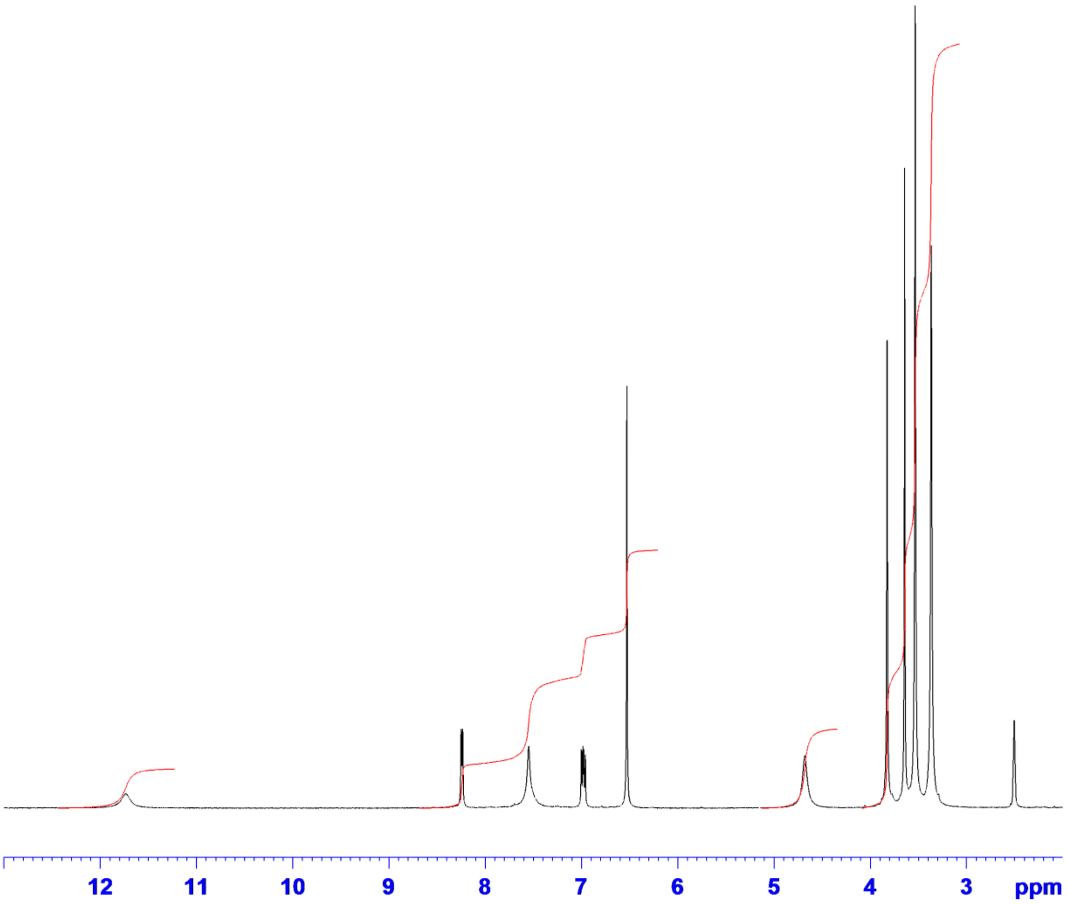

^{1}H NMR of aminopyrazole **40**

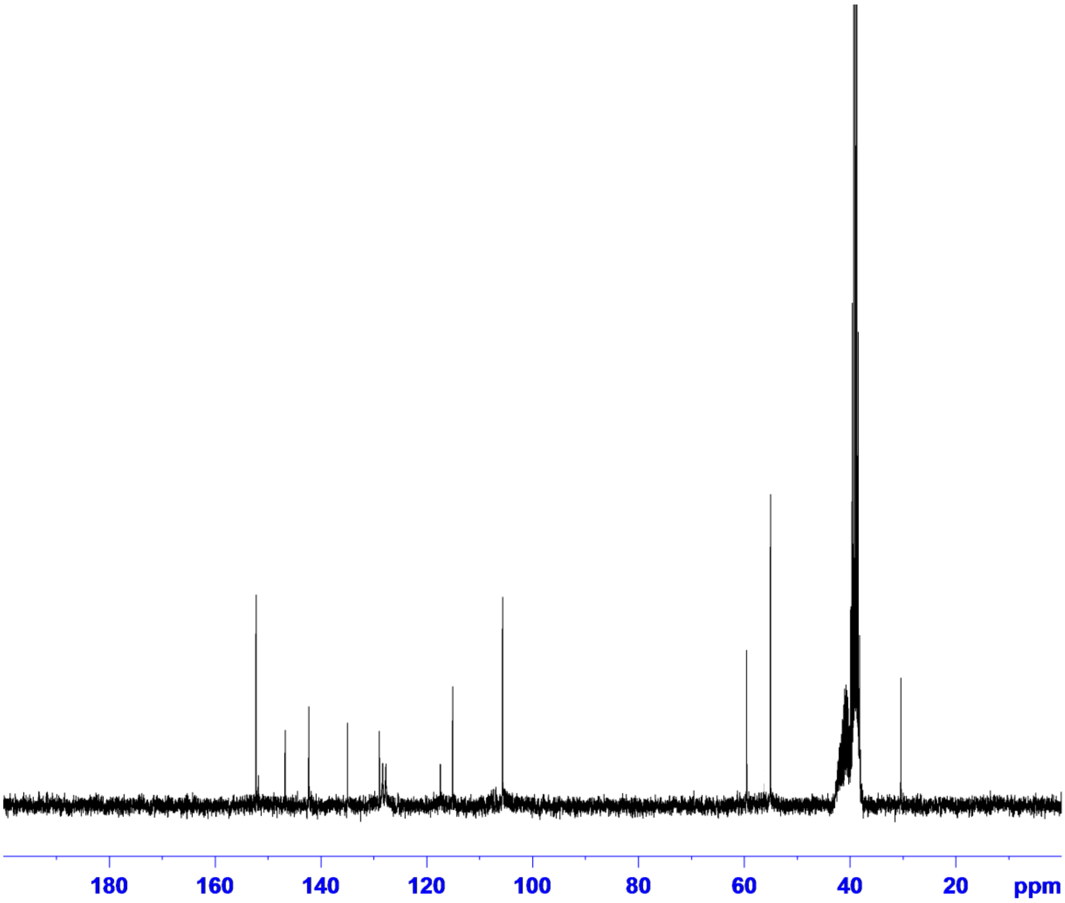

^{13}C NMR of aminopyrazole **40**

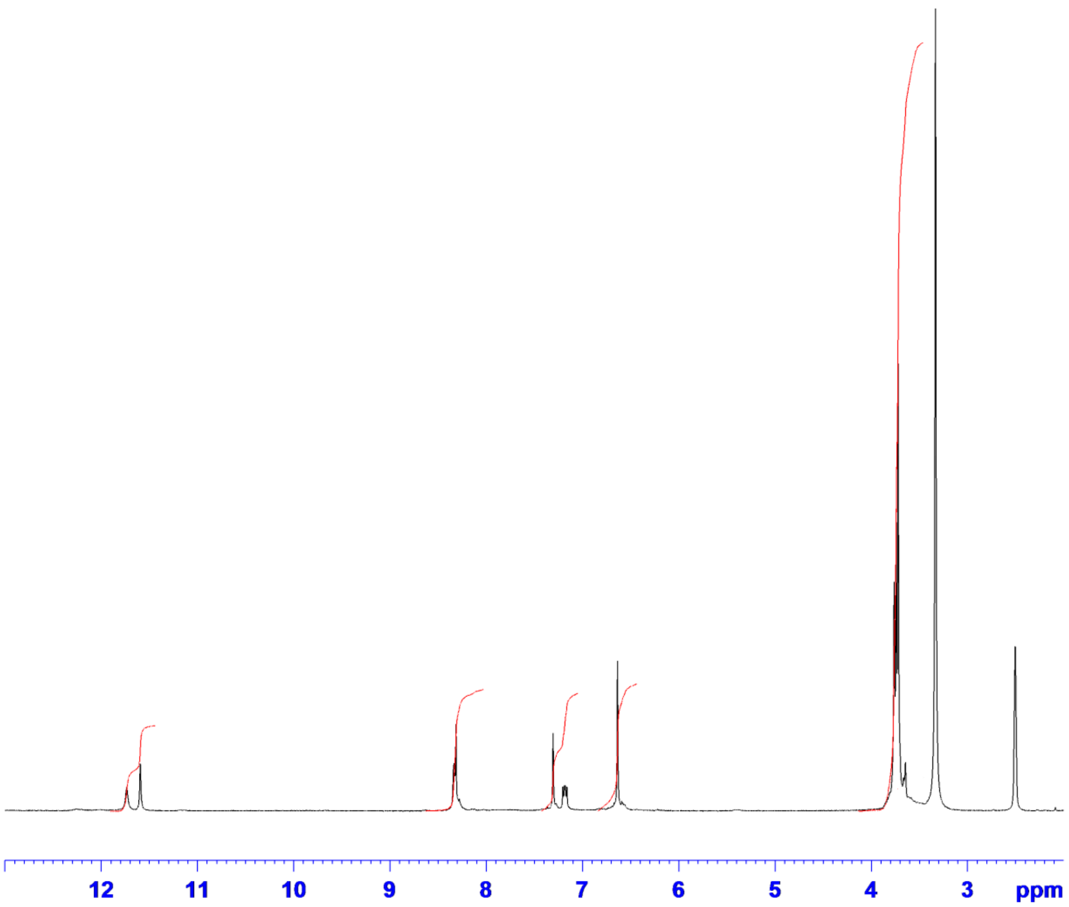

^{1}H NMR of triazinedione **41**

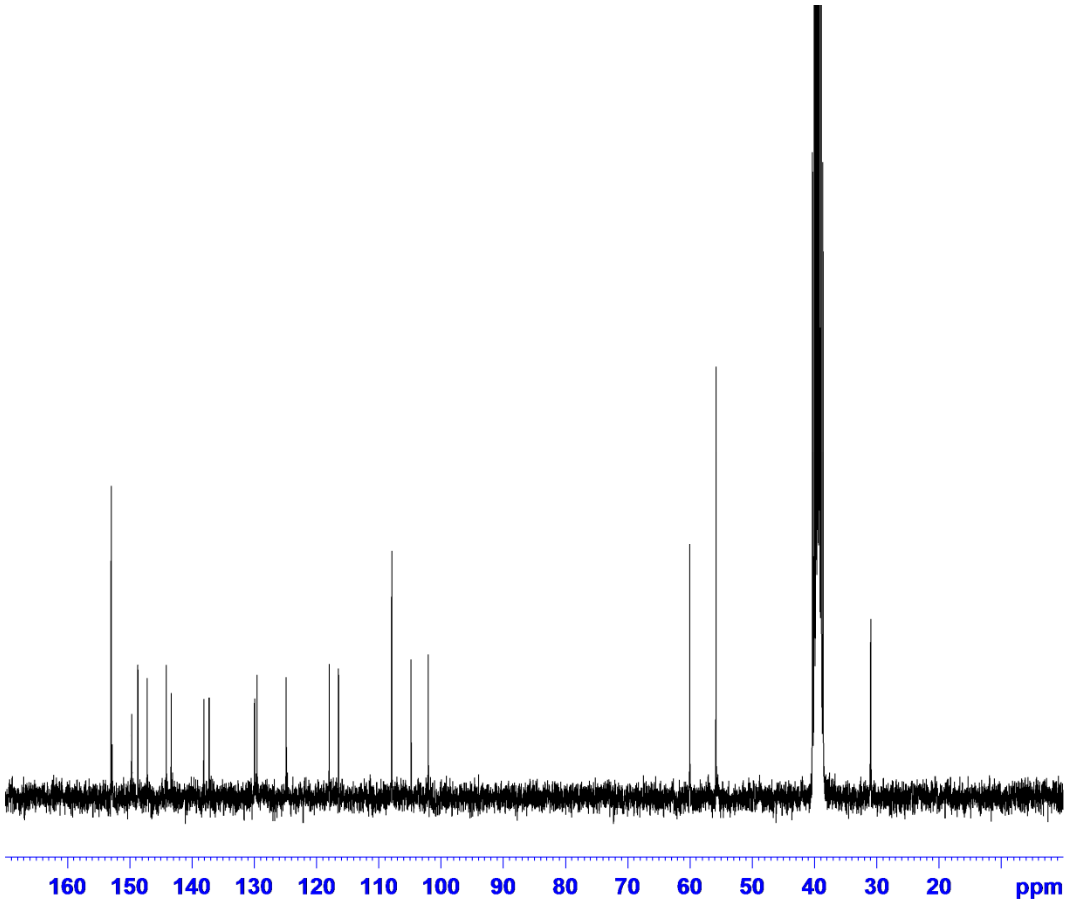

^{13}C NMR of triazinedione **41**

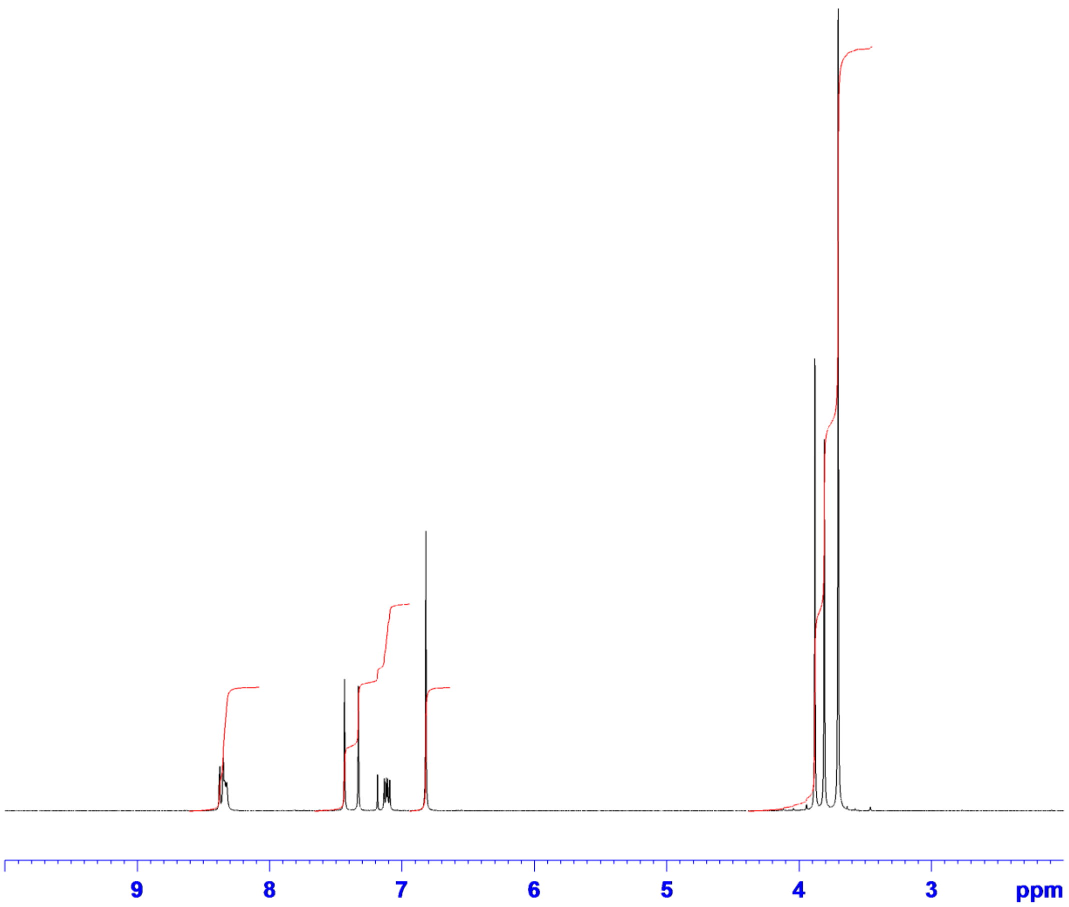

^{1}H NMR of pyrazolo[1,5-*a*]pyrimidine **42**

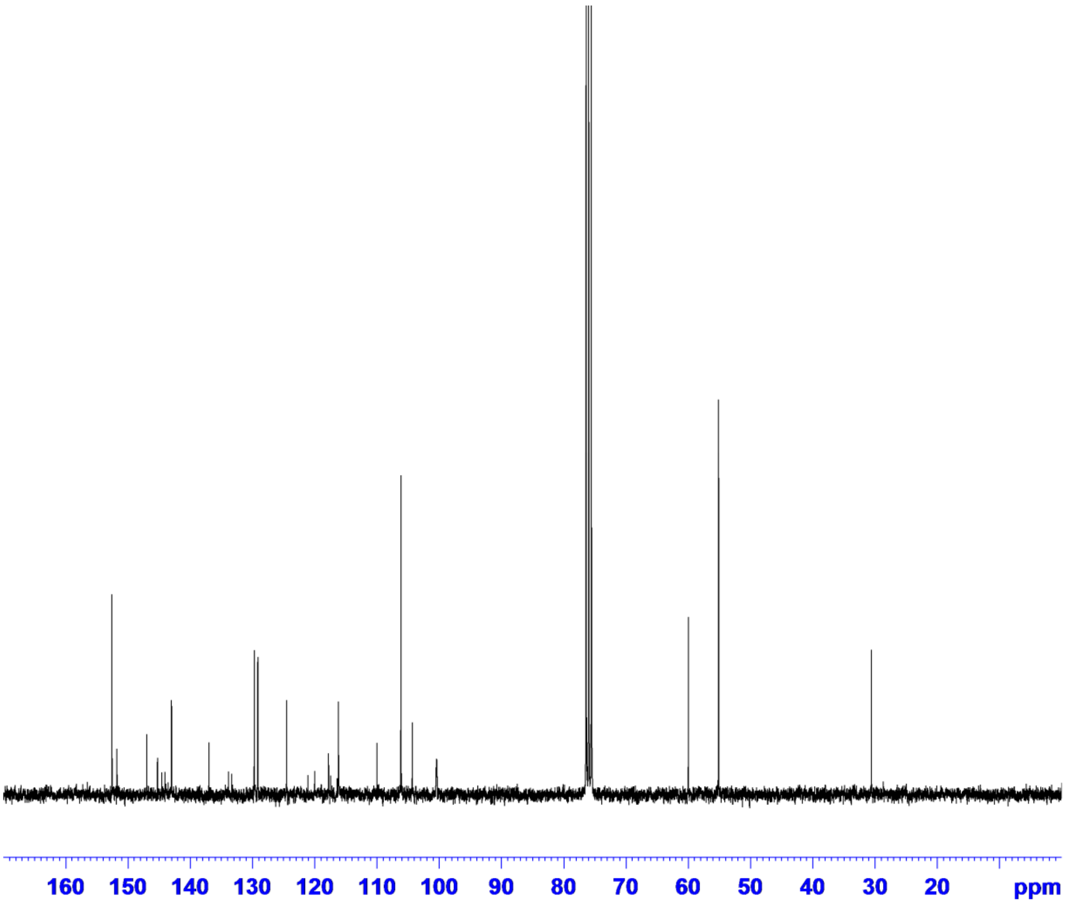

^{13}C NMR of pyrazolo[1,5-*a*]pyrimidine **42**

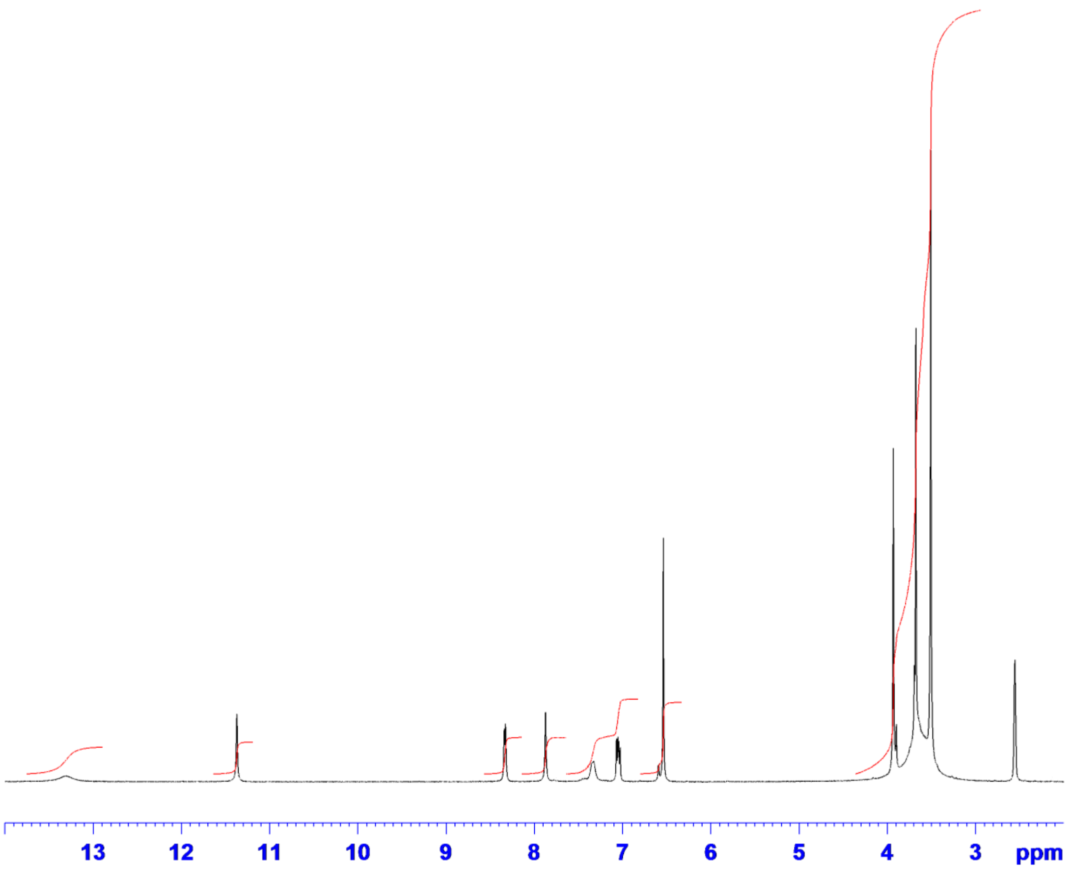

^{1}H NMR of trifluoroacetamide **43**

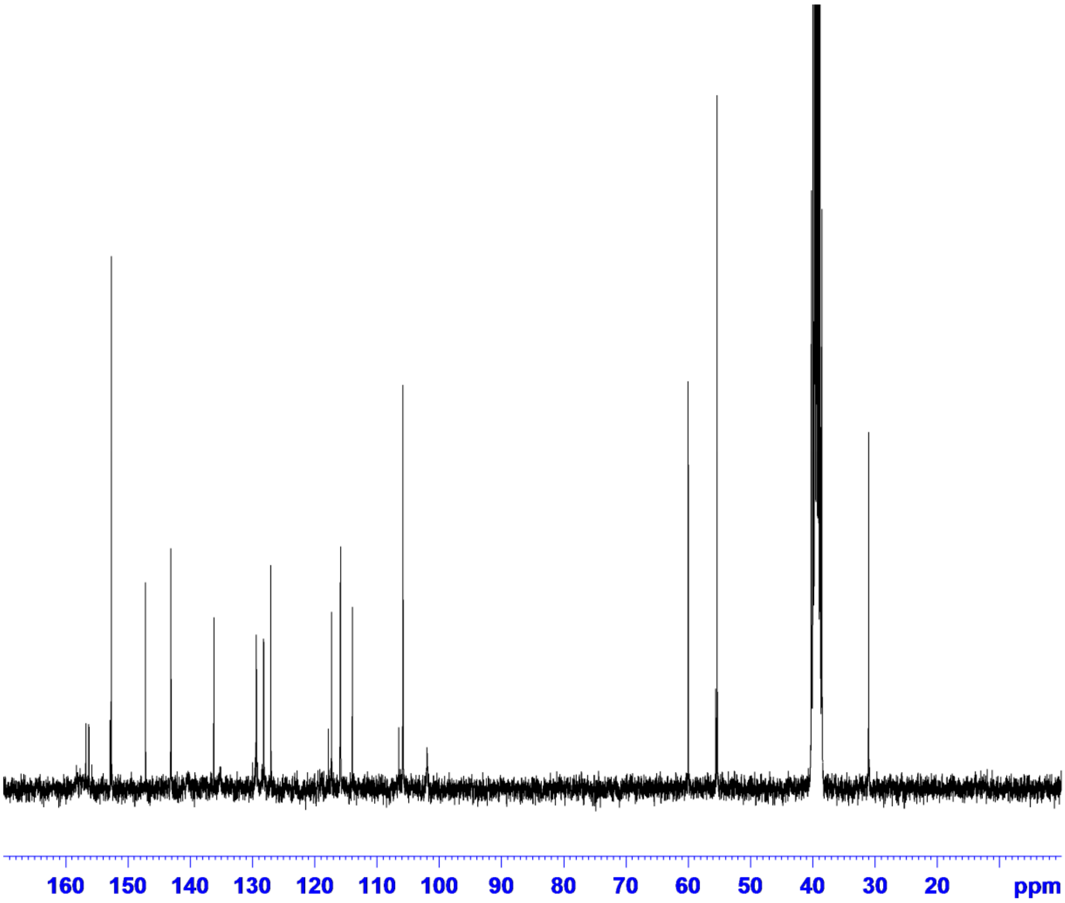

^{13}C NMR of trifluoroacetamide **43**

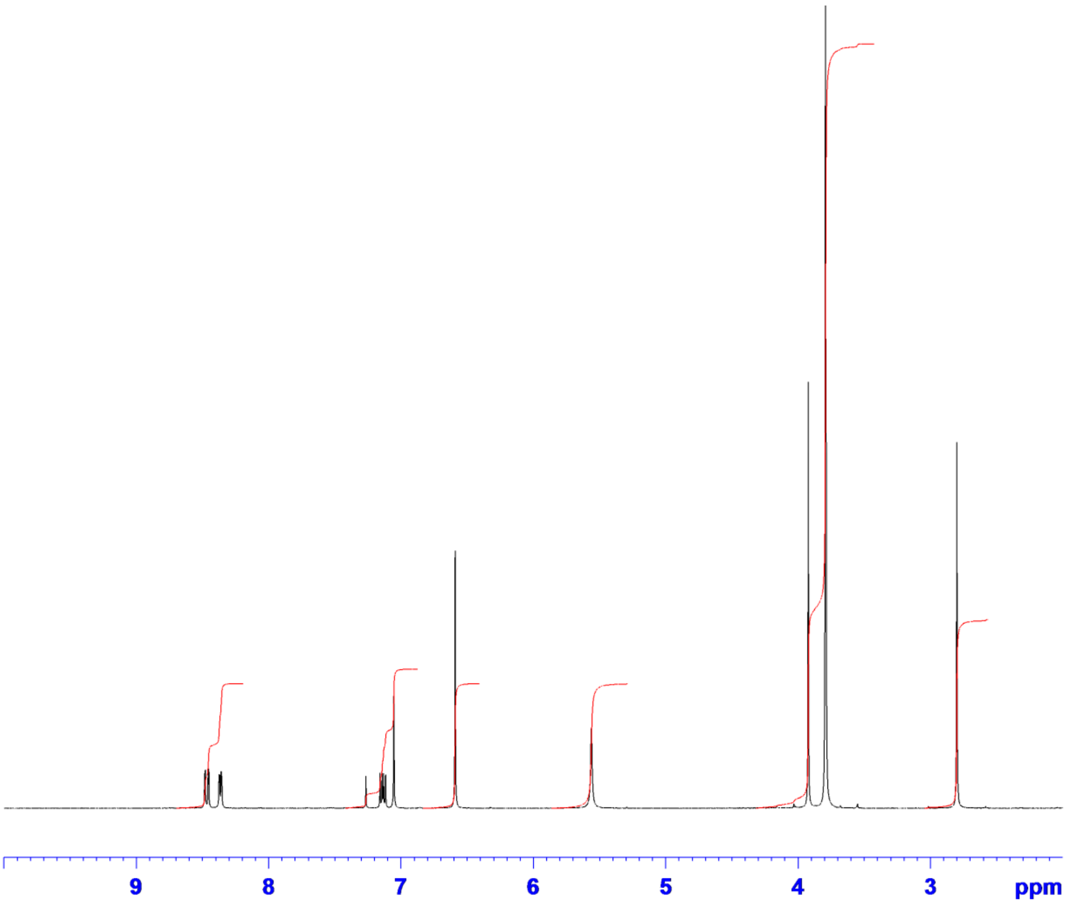

^{1}H NMR of acetylated aminopyrazole **44**

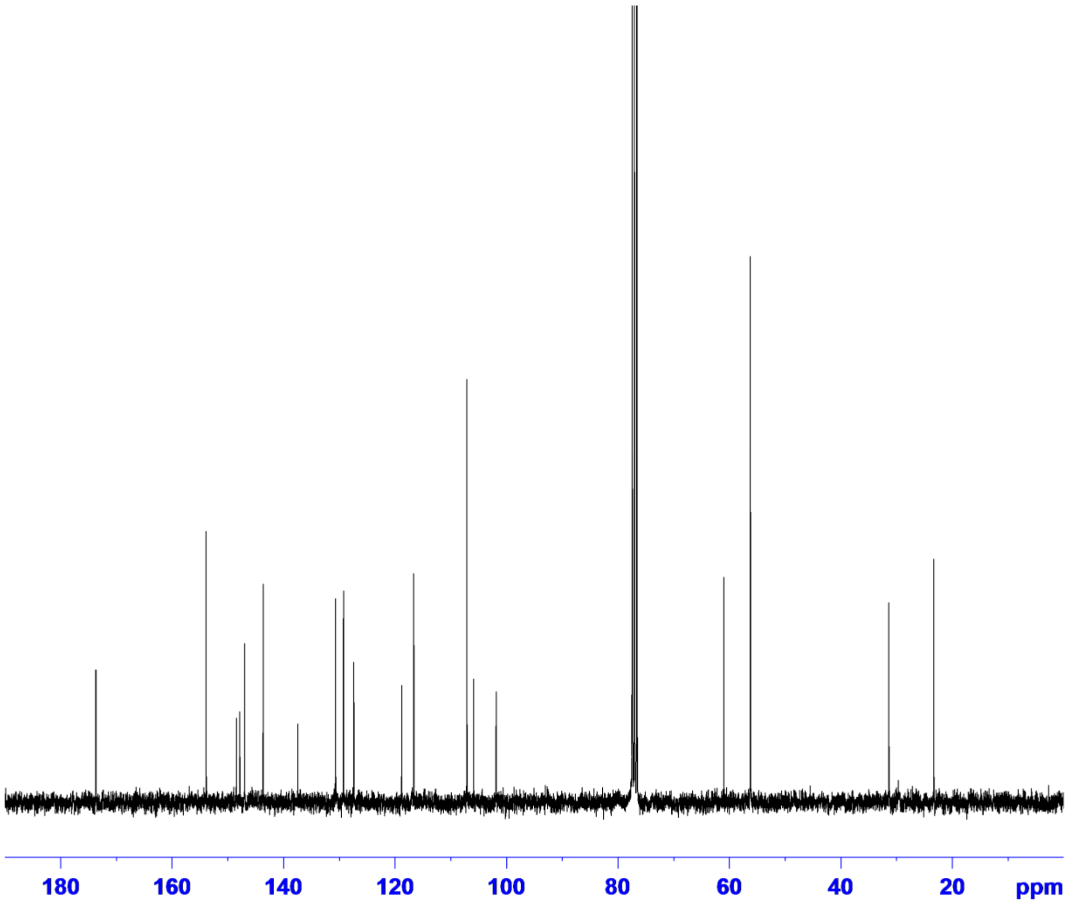

^{13}C NMR of acetylated aminopyrazole **44**

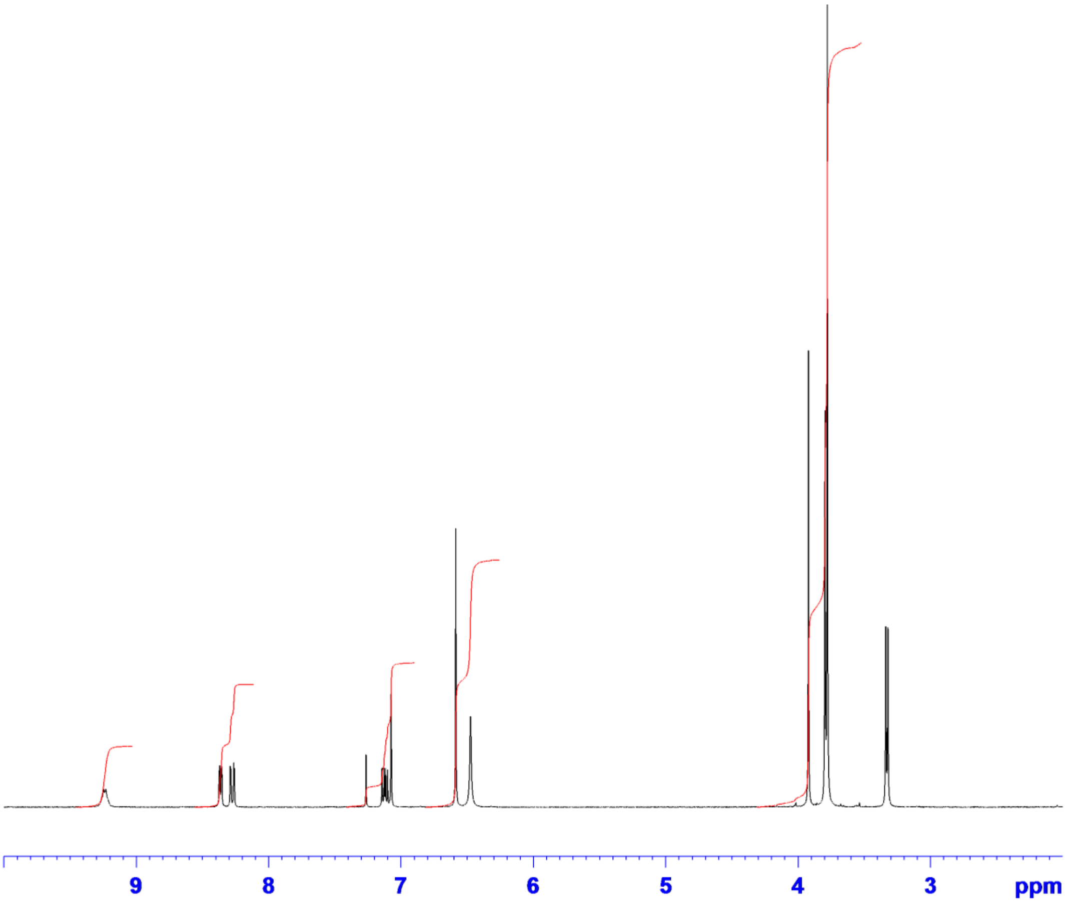

^{1}H NMR of carbothioamide **45**

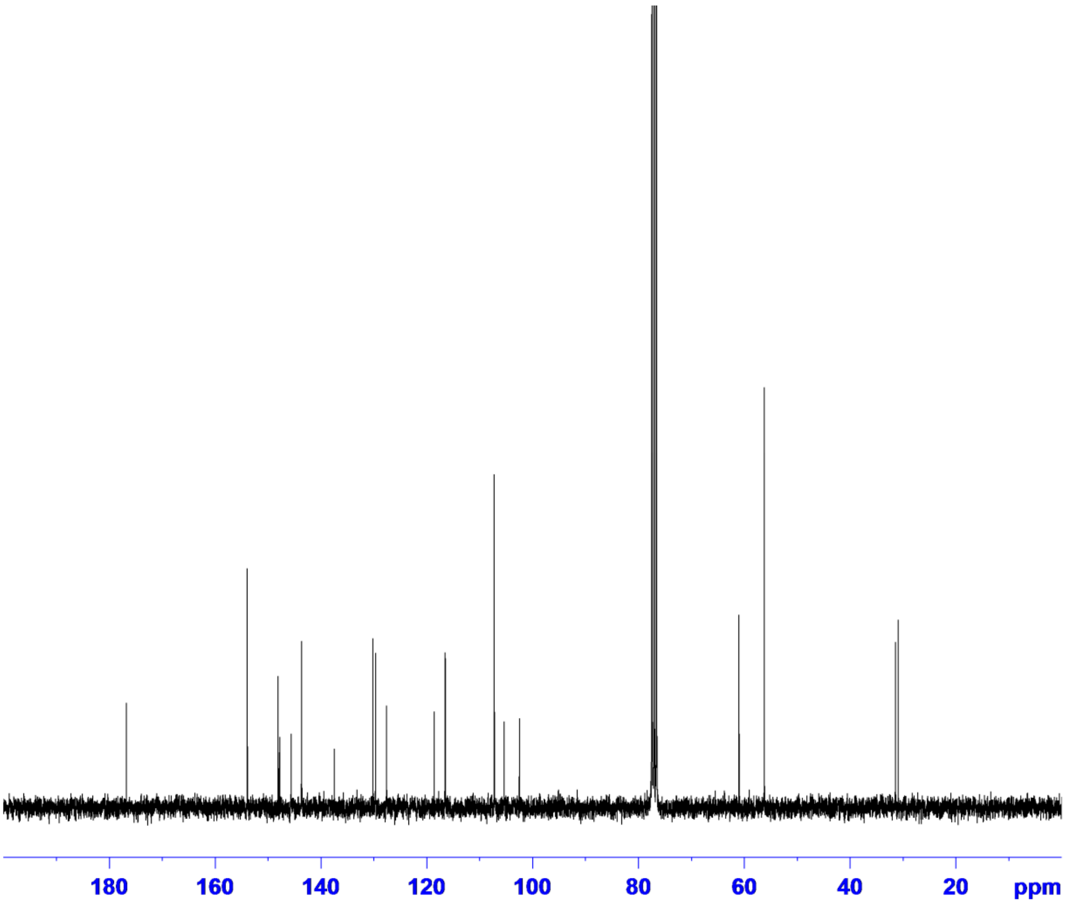

^{13}C NMR of carbothioamide **45**

Crystallography Data

Acetylated aminopyrazole **54**:

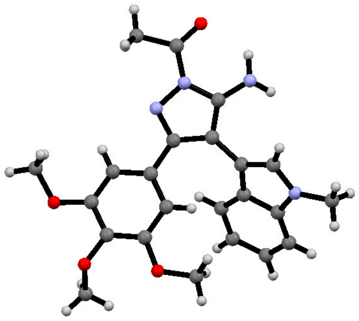

Crystal data: $C_{23}H_{24}N_4O_4$, *M* = 420.46, triclinic, *a* = 10.067(6) Å, *b* = 10.865(7) Å, *c* = 10.939(8) Å, α = 89.55(2)°, β = 70.960(19)°, γ = 82.166(19)°, *V* = 1119.6(12) Å^3, T = 300.0(2) K, space group *P*-1, *Z* = 2, 7316 reflections measured, 3803 unique (R_{int} = 0.049). The final R_1 values were 0.0698 (2181 reflections with $I > 2\sigma(I)$) and 0.1267 (all data). The final $wR(F^2)$ values were 0.1623 ($I > 2\sigma(I)$) and 0.2167 (all data).

Carbothioamide **55**:

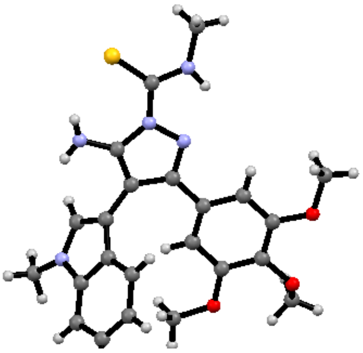

Crystal data: $C_{23}H_{25}N_5O_3S$, *M* = 451.54, monoclinic, *a* = 16.1044(17) Å, *b* = 9.5801(11) Å, *c* = 14.6361(14) Å, β = 92.804(4)°, *V* = 2255.4(4) Å^3, T = 300.0(2) K, space group $P2_1/c$, *Z* = 4, 27235 reflections measured, 5551 unique (R_{int} = 0.049). The final R_1 values were 0.0652 (3533 reflections with $I > 2\sigma(I)$) and 0.1059 (all data). The final $wR(F^2)$ values were 0.1804 ($I > 2\sigma(I)$) and 0.2095 (all data).

Pyrazolo[1,5-*a*]pyrimidine **53**:

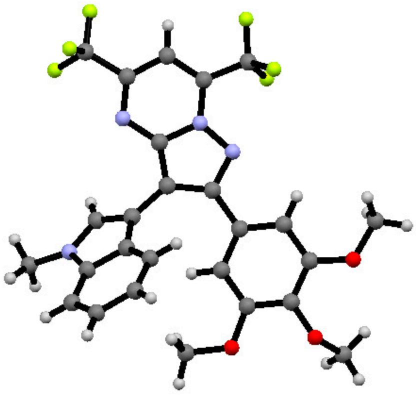

Crystal data: $C_{26}H_{20}F_6N_4O_3$, *M* = 550.46, triclinic, *a* = 13.960(2) Å, *b* = 14.052(2) Å, *c* = 14.580(2) Å, α = 114.173(3)°, β = 108.342(4)°, γ = 93.100(4)°, *V* = 2420.8(7) Å^3, T = 100.0(2) K, space group *P*-1, *Z* = 4, 51437 reflections measured, 9691 unique (R_{int} = 0.0758). The final R_1 values were 0.0484 (5857 reflections with $I > 2\sigma(I)$) and 0.1043 (all data). The final $wR(F^2)$ values were 0.1093 ($I > 2\sigma(I)$) and 0.1411 (all data).

NCI-60 Cell Line Growth Data (One Dose 10 M)

Compound 32

Developmental Therapeutics Program One Dose Mean Graph	NSC: D-776698 / 1	Conc: 1.00E-5 Molar	Test Date: Jul 22, 2013
	Experiment ID: 1307OS38		Report Date: Dec 03, 2013

Panel/Cell Line	Growth Percent
**Leukemia**	
CCRF-CEM	90.02
HL-60(TB)	93.11
K-562	98.75
MOLT-4	90.14
RPMI-8226	91.68
SR	82.07
**Non-Small Cell Lung Cancer**	
A549/ATCC	99.66
HOP-62	87.18
HOP-92	89.62
NCI-H226	89.20
NCI-H23	97.62
NCI-H322M	117.87
NCI-H460	105.84
NCI-H522	82.15
**Colon Cancer**	
COLO 205	100.74
HCC-2998	108.63
HCT-116	100.19
HCT-15	93.87
HT29	100.23
KM12	96.70
SW-620	102.18
**CNS Cancer**	
SF-268	95.35
SF-295	105.83
SF-539	96.49
SNB-19	109.21
SNB-75	90.99
U251	94.52
**Melanoma**	
LOX IMVI	81.04
M14	94.29
MDA-MB-435	100.10
SK-MEL-2	57.95
SK-MEL-28	108.85
SK-MEL-5	95.00
UACC-257	108.95
UACC-62	98.94
**Ovarian Cancer**	
IGROV1	96.09
OVCAR-3	97.38
OVCAR-4	114.07
OVCAR-5	95.33
OVCAR-8	101.54
NCI/ADR-RES	106.37
SK-OV-3	92.91
**Renal Cancer**	
786-0	92.35
A498	92.82
ACHN	102.46
CAKI-1	103.35
RXF 393	105.34
SN12C	102.01
TK-10	89.35
UO-31	71.52
**Prostate Cancer**	
PC-3	85.83
DU-145	106.63
**Breast Cancer**	
MCF7	89.90
MDA-MB-231/ATCC	86.24
HS 578T	105.59
BT-549	88.80
T-47D	82.31
MDA-MB-468	91.79
Mean	95.77
Delta	37.82
Range	59.92

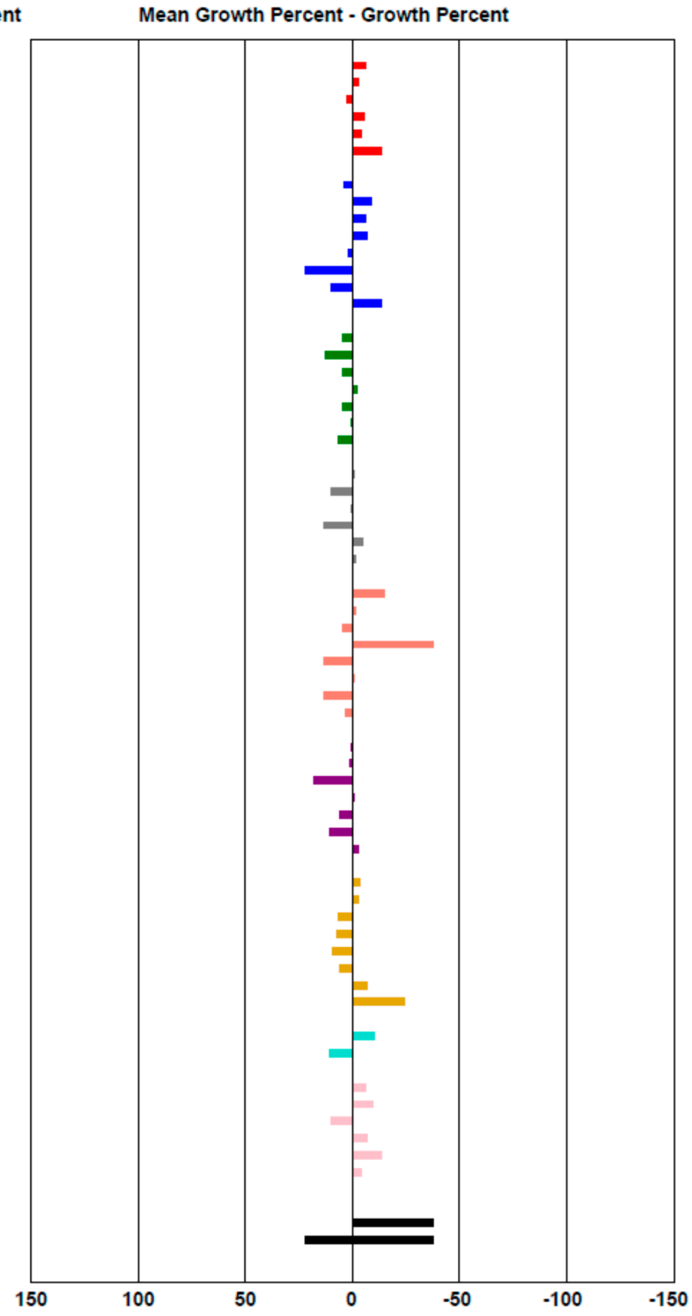

Compound 33

Developmental Therapeutics Program
One Dose Mean Graph

**NSC:** D-776695 / 1	**Conc:** 1.00E-5 Molar	**Test Date:** Jul 22, 2013
**Experiment ID:** 1307OS38		**Report Date:** Dec 03, 2013

Panel/Cell Line	Growth Percent
Leukemia	
CCRF-CEM	89.19
HL-60(TB)	89.75
K-562	87.09
MOLT-4	90.75
RPMI-8226	90.61
SR	87.04
Non-Small Cell Lung Cancer	
A549/ATCC	101.49
HOP-62	83.25
NCI-H226	82.23
NCI-H23	97.16
NCI-H322M	95.69
NCI-H460	104.48
NCI-H522	71.69
Colon Cancer	
COLO 205	97.54
HCC-2998	114.25
HCT-116	94.54
HCT-15	92.90
HT29	99.56
KM12	88.08
SW-620	99.26
CNS Cancer	
SF-268	94.20
SF-295	104.40
SF-539	106.86
SNB-19	95.24
SNB-75	77.91
U251	98.77
Melanoma	
LOX IMVI	92.28
M14	99.70
MDA-MB-435	101.76
SK-MEL-2	97.27
SK-MEL-28	110.90
SK-MEL-5	93.12
UACC-257	98.83
UACC-62	92.94
Ovarian Cancer	
IGROV1	100.10
OVCAR-3	98.94
OVCAR-4	100.67
OVCAR-5	108.24
OVCAR-8	100.72
NCI/ADR-RES	102.85
SK-OV-3	97.27
Renal Cancer	
786-0	97.03
A498	83.10
ACHN	108.28
CAKI-1	100.44
RXF 393	104.13
SN12C	97.55
TK-10	90.31
UO-31	66.36
Prostate Cancer	
PC-3	88.28
DU-145	102.30
Breast Cancer	
MCF7	89.80
MDA-MB-231/ATCC	92.52
HS 578T	107.06
BT-549	99.93
T-47D	82.65
MDA-MB-468	97.10
Mean	95.38
Delta	29.02
Range	47.89

Mean Growth Percent - Growth Percent

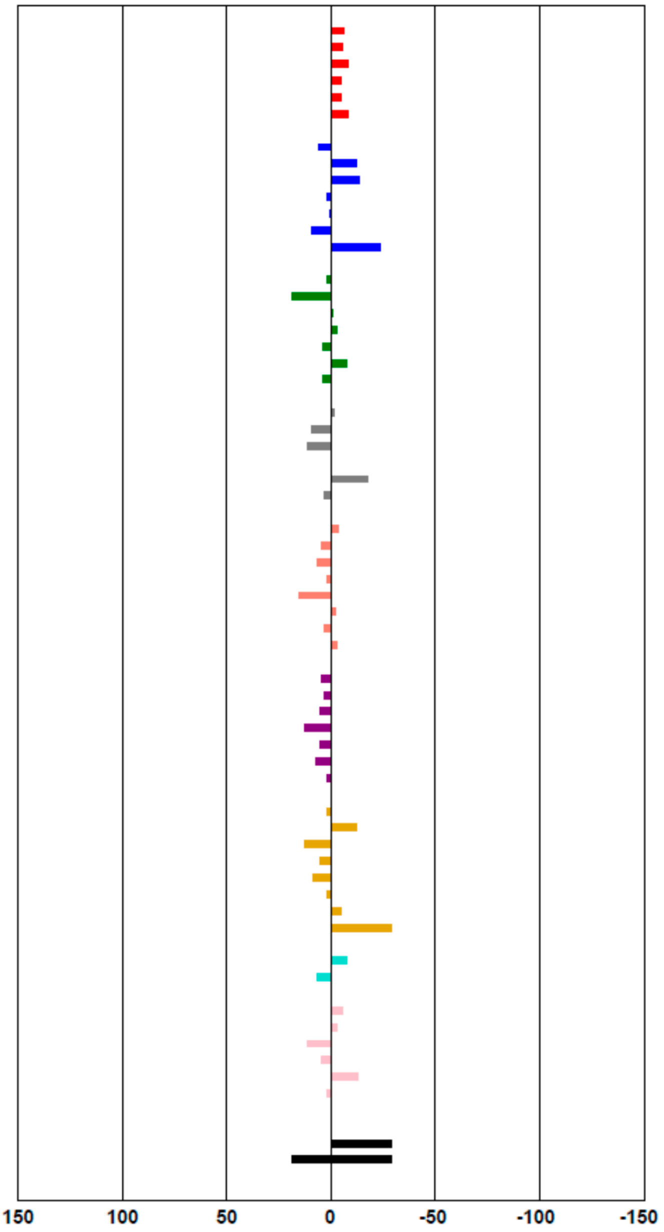

Compound 34

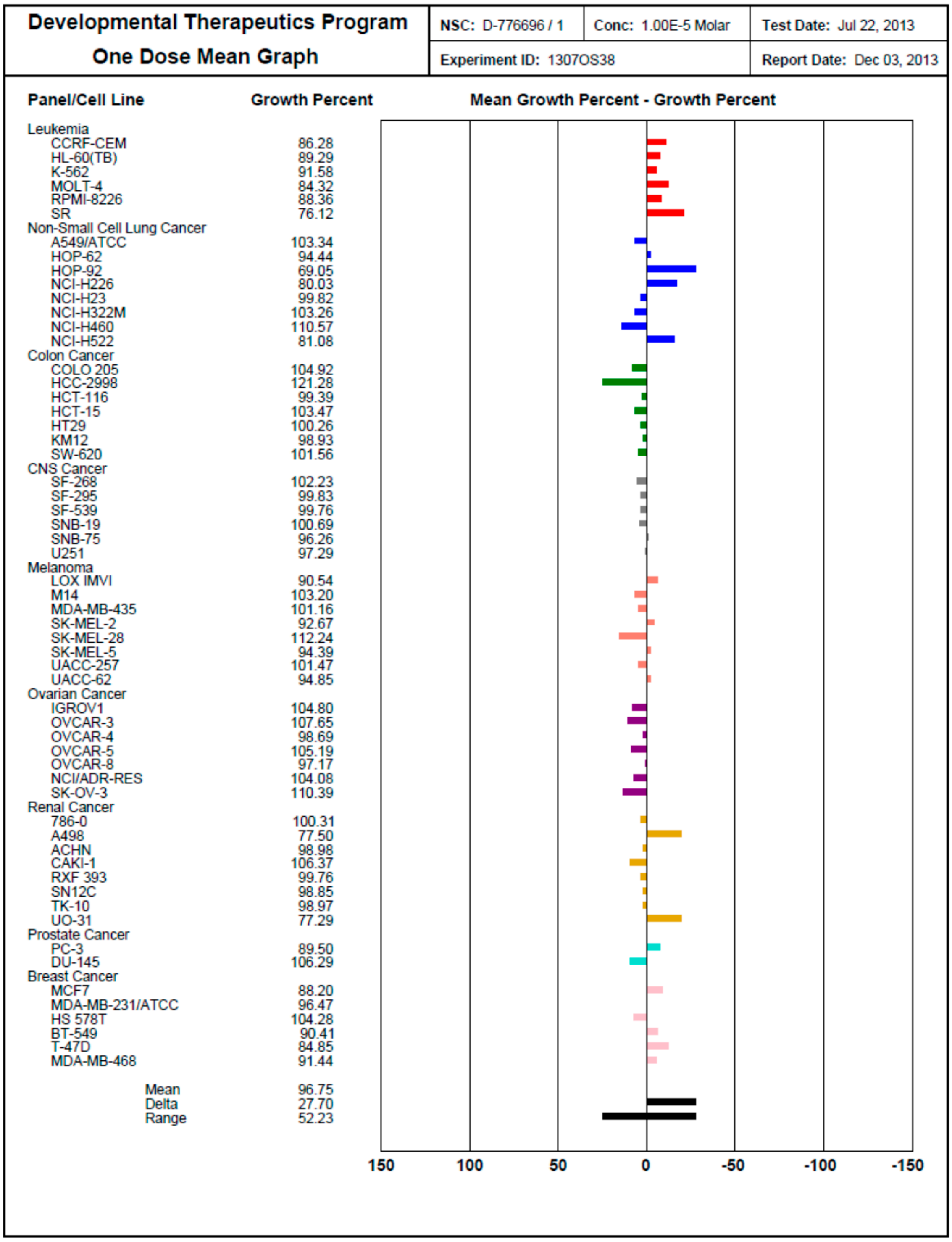
Developmental Therapeutics Program
One Dose Mean Graph
NSC: D-776696 / 1
Conc: 1.00E-5 Molar
Test Date: Jul 22, 2013
Experiment ID: 1307OS38
Report Date: Dec 03, 2013
Panel/Cell Line
Growth Percent
Mean Growth Percent - Growth Percent
Leukemia
CCRF-CEM 86.28
HL-60(TB) 89.29
K-562 91.58
MOLT-4 84.32
RPMI-8226 88.36
SR 76.12
Non-Small Cell Lung Cancer
A549/ATCC 103.34
HOP-62 94.44
HOP-92 69.05
NCI-H226 80.03
NCI-H23 99.82
NCI-H322M 103.26
NCI-H460 110.57
NCI-H522 81.08
Colon Cancer
COLO 205 104.92
HCC-2998 121.28
HCT-116 99.39
HCT-15 103.47
HT29 100.26
KM12 98.93
SW-620 101.56
CNS Cancer
SF-268 102.23
SF-295 99.83
SF-539 99.76
SNB-19 100.69
SNB-75 96.26
U251 97.29
Melanoma
LOX IMVI 90.54
M14 103.20
MDA-MB-435 101.16
SK-MEL-2 92.67
SK-MEL-28 112.24
SK-MEL-5 94.39
UACC-257 101.47
UACC-62 94.85
Ovarian Cancer
IGROV1 104.80
OVCAR-3 107.65
OVCAR-4 98.69
OVCAR-5 105.19
OVCAR-8 97.17
NCI/ADR-RES 104.08
SK-OV-3 110.39
Renal Cancer
786-0 100.31
A498 77.50
ACHN 98.98
CAKI-1 106.37
RXF 393 99.76
SN12C 98.85
TK-10 98.97
UO-31 77.29
Prostate Cancer
PC-3 89.50
DU-145 106.29
Breast Cancer
MCF7 88.20
MDA-MB-231/ATCC 96.47
HS 578T 104.28
BT-549 90.41
T-47D 84.85
MDA-MB-468 91.44
Mean 96.75
Delta 27.70
Range 52.23
150
100
50
0
-50
-100
-150

Compound 35

Developmental Therapeutics Program One Dose Mean Graph	NSC: D-776697 / 1	Conc: 1.00E-5 Molar	Test Date: Jul 22, 2013
	Experiment ID: 1307OS38		Report Date: Dec 03, 2013

Panel/Cell Line	Growth Percent
Leukemia	
CCRF-CEM	91.28
HL-60(TB)	75.37
K-562	99.35
MOLT-4	86.10
RPMI-8226	99.38
SR	75.88
Non-Small Cell Lung Cancer	
A549/ATCC	98.10
HOP-62	109.58
HOP-92	83.38
NCI-H226	94.34
NCI-H23	99.20
NCI-H322M	102.46
NCI-H460	106.36
NCI-H522	77.19
Colon Cancer	
COLO 205	103.61
HCC-2998	126.80
HCT-116	96.60
HCT-15	98.70
HT29	104.15
KM12	100.26
SW-620	104.09
CNS Cancer	
SF-268	98.56
SF-295	103.07
SF-539	98.93
SNB-19	97.06
SNB-75	93.98
U251	97.31
Melanoma	
LOX IMVI	98.67
M14	99.57
MDA-MB-435	102.94
SK-MEL-2	85.86
SK-MEL-28	108.21
SK-MEL-5	95.72
UACC-257	118.78
UACC-62	103.12
Ovarian Cancer	
IGROV1	100.16
OVCAR-3	107.36
OVCAR-4	102.96
OVCAR-5	101.52
OVCAR-8	103.32
NCI/ADR-RES	109.91
SK-OV-3	103.04
Renal Cancer	
786-0	92.52
A498	83.11
ACHN	107.33
CAKI-1	101.85
RXF 393	105.23
SN12C	101.38
TK-10	100.63
UO-31	86.13
Prostate Cancer	
PC-3	88.72
DU-145	103.92
Breast Cancer	
MCF7	92.91
MDA-MB-231/ATCC	102.80
HS 578T	100.49
BT-549	86.02
T-47D	92.81
MDA-MB-468	99.78
Mean	98.41
Delta	23.04
Range	51.43

Mean Growth Percent - Growth Percent

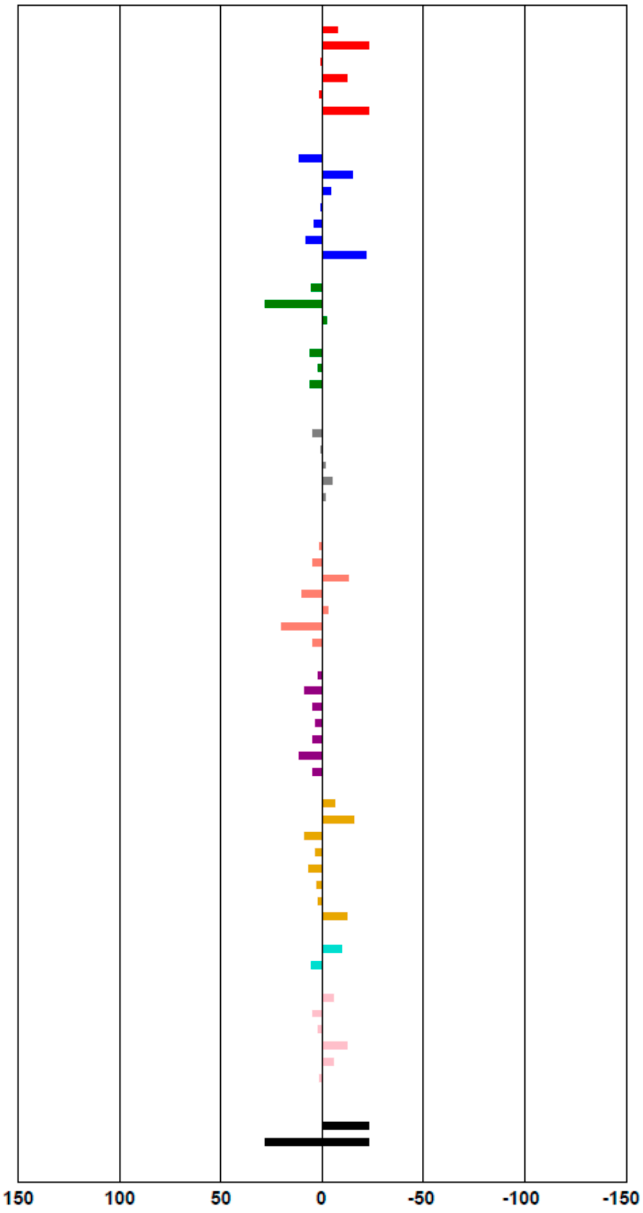

Compound 40

Developmental Therapeutics Program One Dose Mean Graph	NSC: D-763892 / 1	Conc: 1.00E-5 Molar	Test Date: Feb 27, 2012
	Experiment ID: 1202OS23		Report Date: Apr 02, 2012

Panel/Cell Line	Growth Percent
**Leukemia**	
CCRF-CEM	74.89
HL-60(TB)	76.66
K-562	78.54
MOLT-4	90.19
RPMI-8226	69.95
SR	60.73
**Non-Small Cell Lung Cancer**	
A549/ATCC	79.49
EKVX	92.27
HOP-62	66.50
HOP-92	47.90
NCI-H23	85.75
NCI-H322M	92.36
NCI-H460	88.70
NCI-H522	56.85
**Colon Cancer**	
COLO 205	70.18
HCT-116	90.24
HCT-15	94.84
HT29	68.31
KM12	88.29
SW-620	86.56
**CNS Cancer**	
SF-268	104.05
SF-295	78.37
SF-539	87.26
SNB-19	87.26
SNB-75	34.43
U251	80.44
**Melanoma**	
LOX IMVI	84.45
MALME-3M	80.57
M14	89.71
MDA-MB-435	74.30
SK-MEL-2	89.87
SK-MEL-28	82.79
UACC-257	77.88
UACC-62	52.99
**Ovarian Cancer**	
IGROV1	112.92
OVCAR-3	102.22
OVCAR-4	76.38
OVCAR-5	89.37
OVCAR-8	81.80
NCI/ADR-RES	100.68
SK-OV-3	79.07
**Renal Cancer**	
786-0	91.80
A498	64.75
ACHN	95.64
CAKI-1	48.94
RXF 393	104.09
SN12C	80.12
TK-10	76.57
UO-31	49.60
**Prostate Cancer**	
PC-3	81.93
DU-145	110.19
**Breast Cancer**	
MCF7	63.01
MDA-MB-231/ATCC	77.02
HS 578T	61.50
BT-549	97.29
T-47D	70.53
MDA-MB-468	70.69
Mean	79.82
Delta	45.39
Range	78.49

Mean Growth Percent - Growth Percent

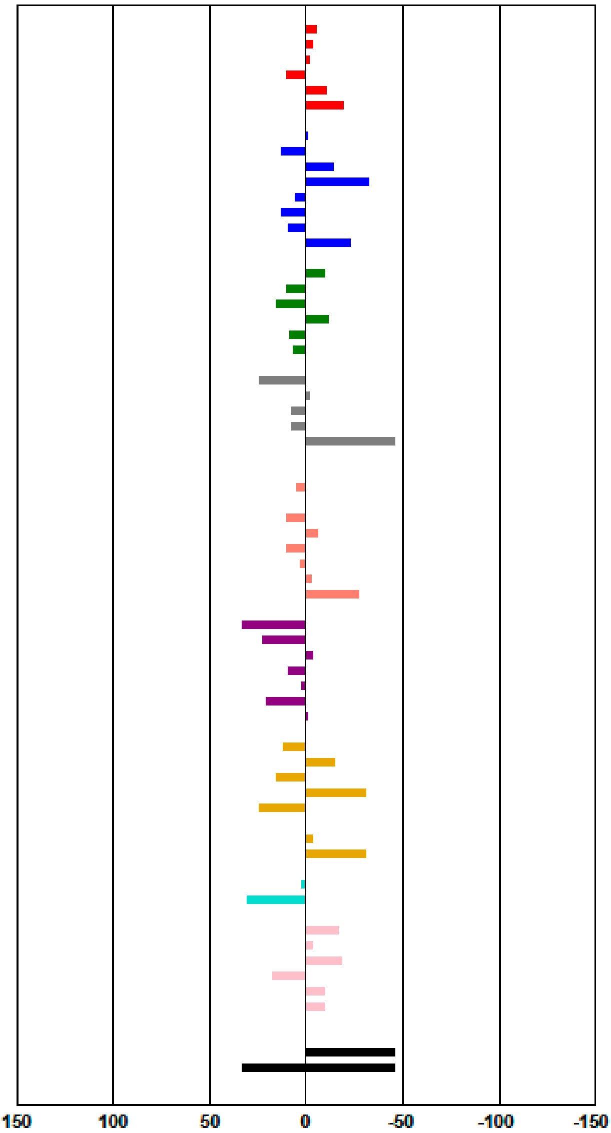

Compound 41

Developmental Therapeutics Program One Dose Mean Graph	NSC: D-763893 / 1	Conc: 1.00E-5 Molar	Test Date: Feb 27, 2012
	Experiment ID: 1202OS23		Report Date: Apr 02, 2012

Panel/Cell Line	Growth Percent
**Leukemia**	
CCRF-CEM	90.21
HL-60(TB)	97.77
K-562	106.96
MOLT-4	94.52
RPMI-8226	88.39
SR	82.16
**Non-Small Cell Lung Cancer**	
A549/ATCC	99.23
EKVX	109.20
HOP-62	108.54
HOP-92	87.70
NCI-H23	95.25
NCI-H322M	115.45
NCI-H460	104.31
NCI-H522	82.43
**Colon Cancer**	
COLO 205	104.73
HCC-2998	99.87
HCT-116	111.99
HCT-15	108.21
HT29	102.08
KM12	108.33
SW-620	106.74
**CNS Cancer**	
SF-268	114.78
SF-295	89.91
SF-539	103.04
SNB-19	96.97
SNB-75	67.20
U251	102.77
**Melanoma**	
LOX IMVI	99.06
MALME-3M	117.81
M14	112.19
MDA-MB-435	100.76
SK-MEL-2	105.39
SK-MEL-28	93.43
UACC-257	101.09
UACC-62	92.46
**Ovarian Cancer**	
IGROV1	134.61
OVCAR-3	119.99
OVCAR-4	94.98
OVCAR-5	111.62
OVCAR-8	101.50
NCI/ADR-RES	106.22
SK-OV-3	105.70
**Renal Cancer**	
786-0	114.85
A498	79.85
ACHN	113.77
CAKI-1	89.78
RXF 393	109.73
SN12C	101.70
TK-10	101.92
UO-31	94.15
**Prostate Cancer**	
PC-3	96.21
DU-145	114.91
**Breast Cancer**	
MCF7	87.21
MDA-MB-231/ATCC	98.24
HS 578T	89.90
BT-549	118.67
T-47D	101.55
MDA-MB-468	98.09
Mean	101.48
Delta	34.28
Range	67.41

Mean Growth Percent - Growth Percent

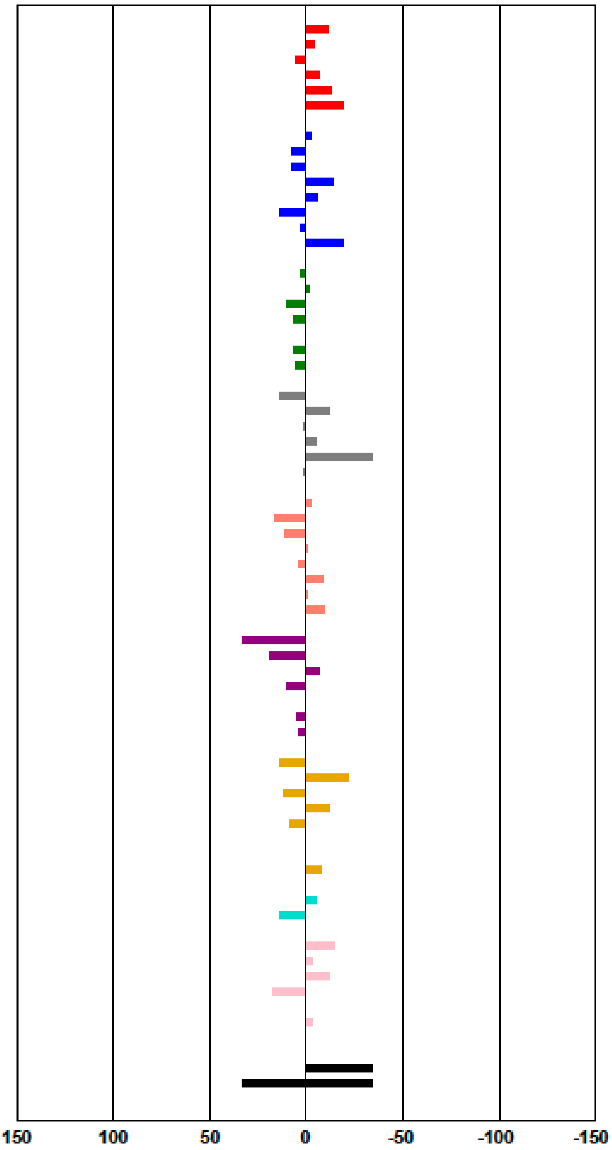

Compound 42

Developmental Therapeutics Program One Dose Mean Graph	NSC: D-763894 / 1	Conc: 1.00E-5 Molar	Test Date: Feb 27, 2012
	Experiment ID: 1202OS23		Report Date: Apr 02, 2012

Panel/Cell Line	Growth Percent
**Leukemia**	
CCRF-CEM	89.32
HL-60(TB)	103.42
K-562	103.13
MOLT-4	93.25
RPMI-8226	70.57
SR	85.31
**Non-Small Cell Lung Cancer**	
A549/ATCC	91.78
EKVX	75.49
HOP-62	98.88
NCI-H23	90.32
NCI-H322M	102.84
NCI-H460	102.45
NCI-H522	83.64
**Colon Cancer**	
COLO 205	99.17
HCC-2998	101.04
HCT-116	95.01
HCT-15	100.95
HT29	104.54
KM12	103.08
SW-620	99.39
**CNS Cancer**	
SF-268	104.30
SF-295	104.02
SF-539	105.20
SNB-19	94.81
SNB-75	73.15
U251	98.11
**Melanoma**	
LOX IMVI	83.00
MALME-3M	117.38
M14	109.96
MDA-MB-435	100.94
SK-MEL-2	88.13
SK-MEL-28	99.49
UACC-257	94.53
UACC-62	90.94
**Ovarian Cancer**	
IGROV1	101.10
OVCAR-3	115.86
OVCAR-4	76.03
OVCAR-5	121.15
OVCAR-8	91.85
NCI/ADR-RES	86.71
SK-OV-3	104.16
**Renal Cancer**	
786-0	115.54
A498	74.49
ACHN	95.55
CAKI-1	82.28
RXF 393	95.74
SN12C	88.16
TK-10	117.49
UO-31	71.23
**Prostate Cancer**	
PC-3	81.22
DU-145	111.62
**Breast Cancer**	
MCF7	91.67
MDA-MB-231/ATCC	73.09
HS 578T	84.95
BT-549	117.21
T-47D	90.63
MDA-MB-468	92.09
Mean	95.39
Delta	24.82
Range	50.58

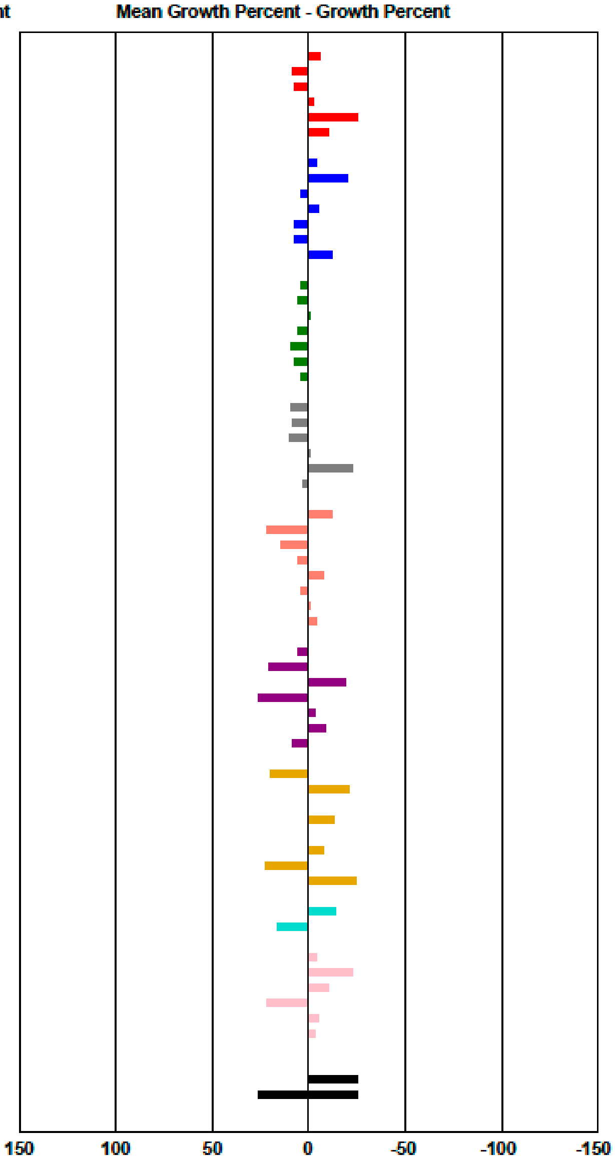

Compound 44

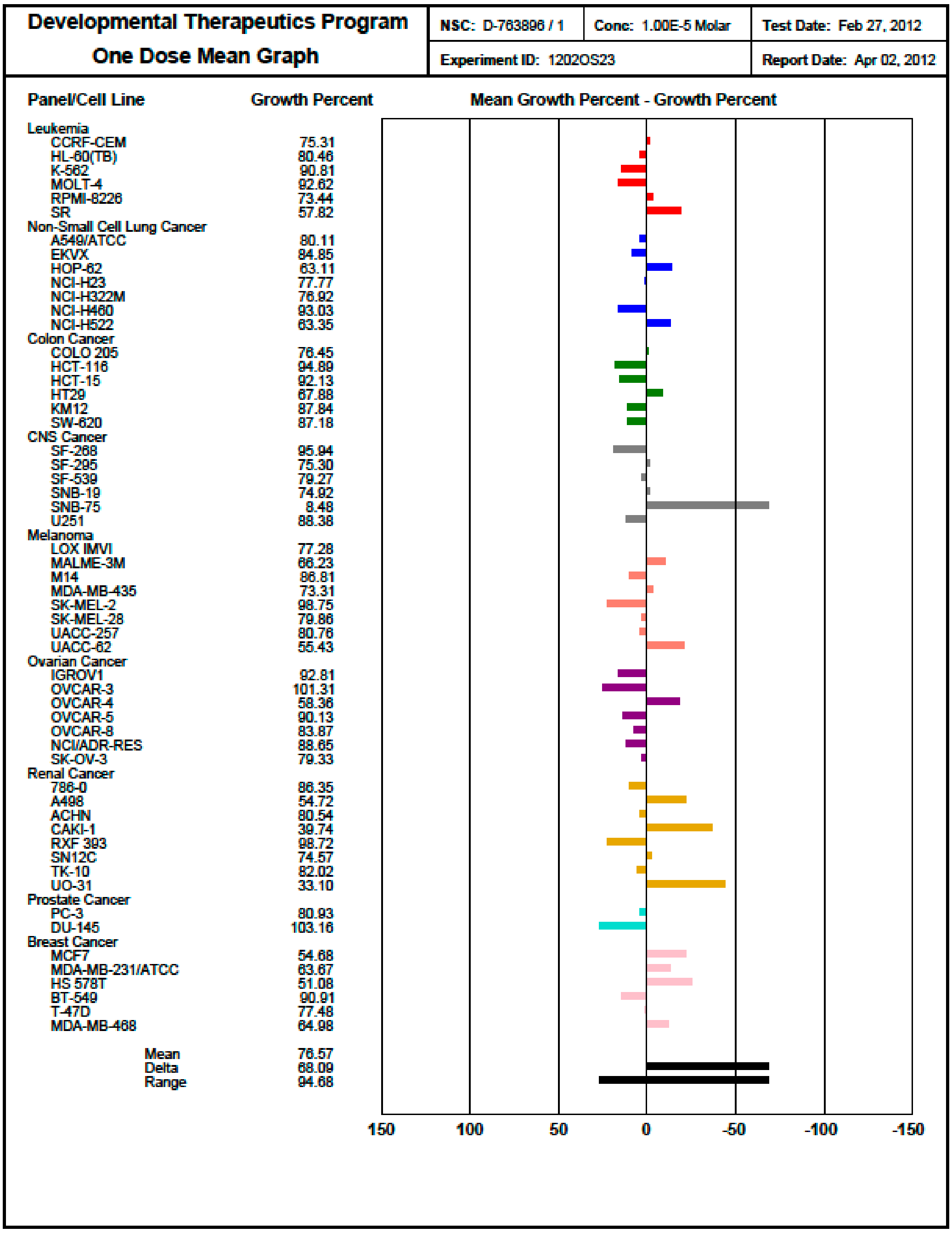

Compound 45

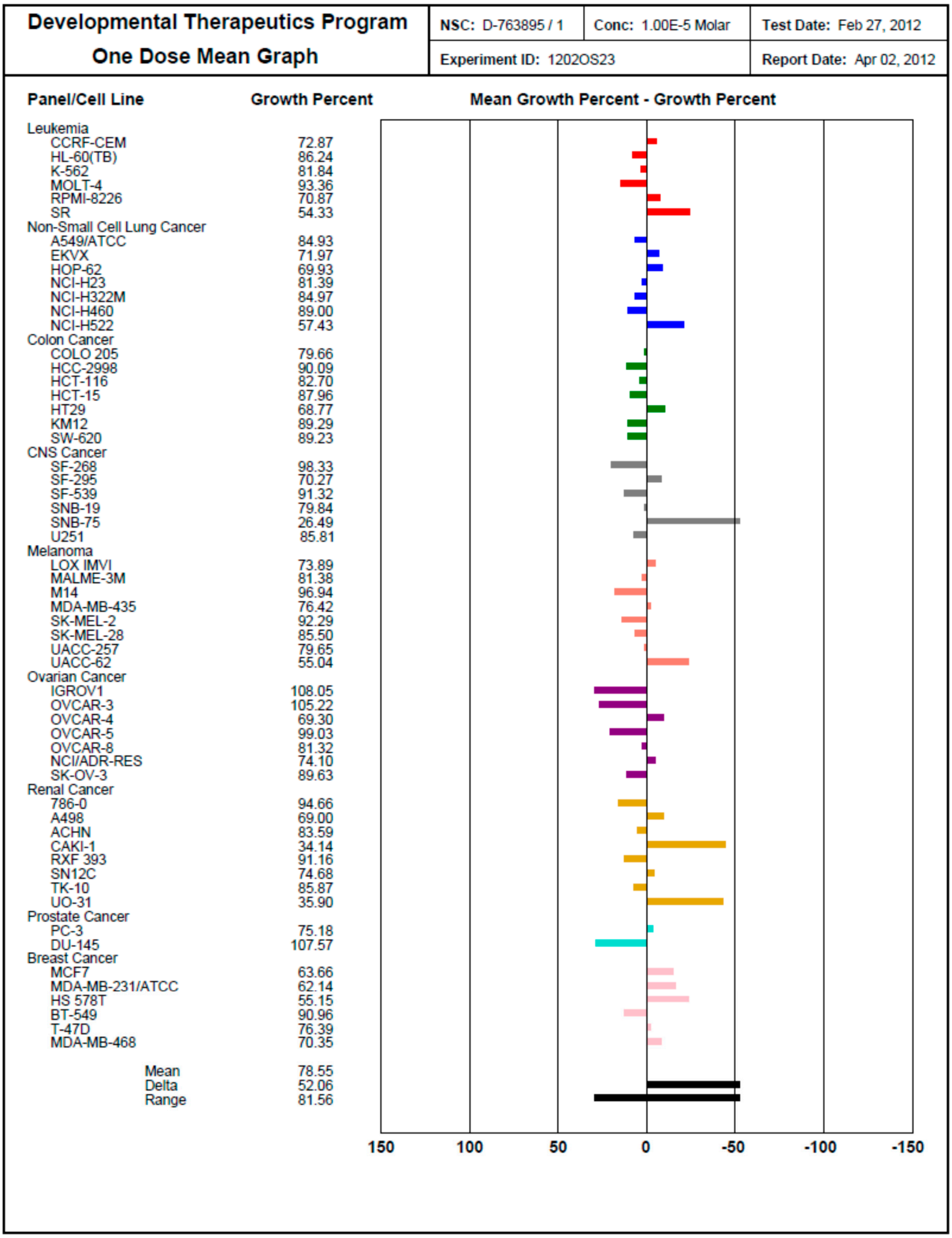
Developmental Therapeutics Program
One Dose Mean Graph
NSC: D-763895 / 1
Conc: 1.00E-5 Molar
Test Date: Feb 27, 2012
Experiment ID: 1202OS23
Report Date: Apr 02, 2012
Panel/Cell Line
Growth Percent
Mean Growth Percent - Growth Percent
Leukemia
CCRF-CEM 72.87
HL-60(TB) 86.24
K-562 81.84
MOLT-4 93.36
RPMI-8226 70.87
SR 54.33
Non-Small Cell Lung Cancer
A549/ATCC 84.93
EKVX 71.97
HOP-62 69.93
NCI-H23 81.39
NCI-H322M 84.97
NCI-H460 89.00
NCI-H522 57.43
Colon Cancer
COLO 205 79.66
HCC-2998 90.09
HCT-116 82.70
HCT-15 87.96
HT29 68.77
KM12 89.29
SW-620 89.23
CNS Cancer
SF-268 98.33
SF-295 70.27
SF-539 91.32
SNB-19 79.84
SNB-75 26.49
U251 85.81
Melanoma
LOX IMVI 73.89
MALME-3M 81.38
M14 96.94
MDA-MB-435 76.42
SK-MEL-2 92.29
SK-MEL-28 85.50
UACC-257 79.65
UACC-62 55.04
Ovarian Cancer
IGROV1 108.05
OVCAR-3 105.22
OVCAR-4 69.30
OVCAR-5 99.03
OVCAR-8 81.32
NCI/ADR-RES 74.10
SK-OV-3 89.63
Renal Cancer
786-0 94.66
A498 69.00
ACHN 83.59
CAKI-1 34.14
RXF 393 91.16
SN12C 74.68
TK-10 85.87
UO-31 35.90
Prostate Cancer
PC-3 75.18
DU-145 107.57
Breast Cancer
MCF7 63.66
MDA-MB-231/ATCC 62.14
HS 578T 55.15
BT-549 90.96
T-47D 76.39
MDA-MB-468 70.35
Mean 78.55
Delta 52.06
Range 81.56
150
100
50
0
-50
-100
-150

Compound 46

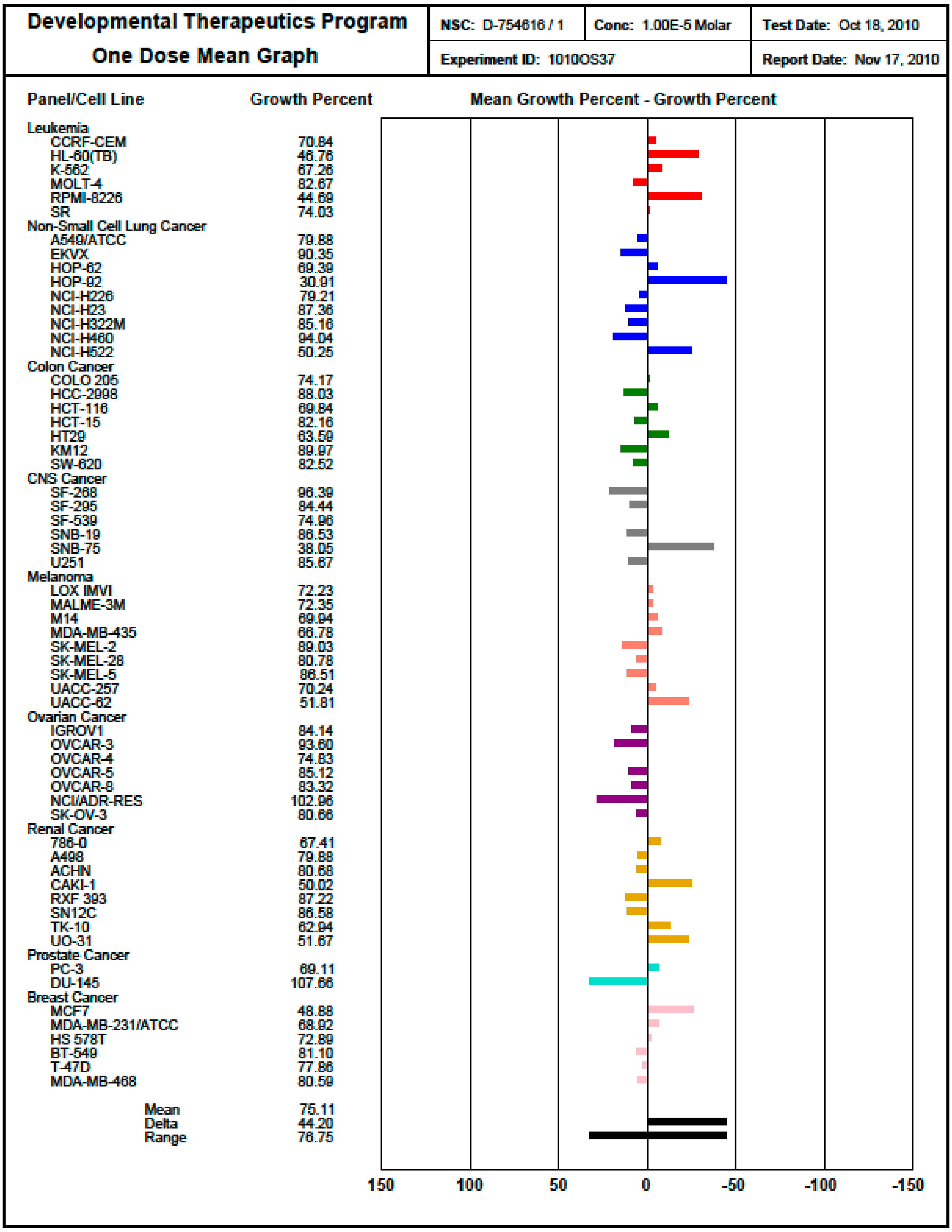

Compound 47

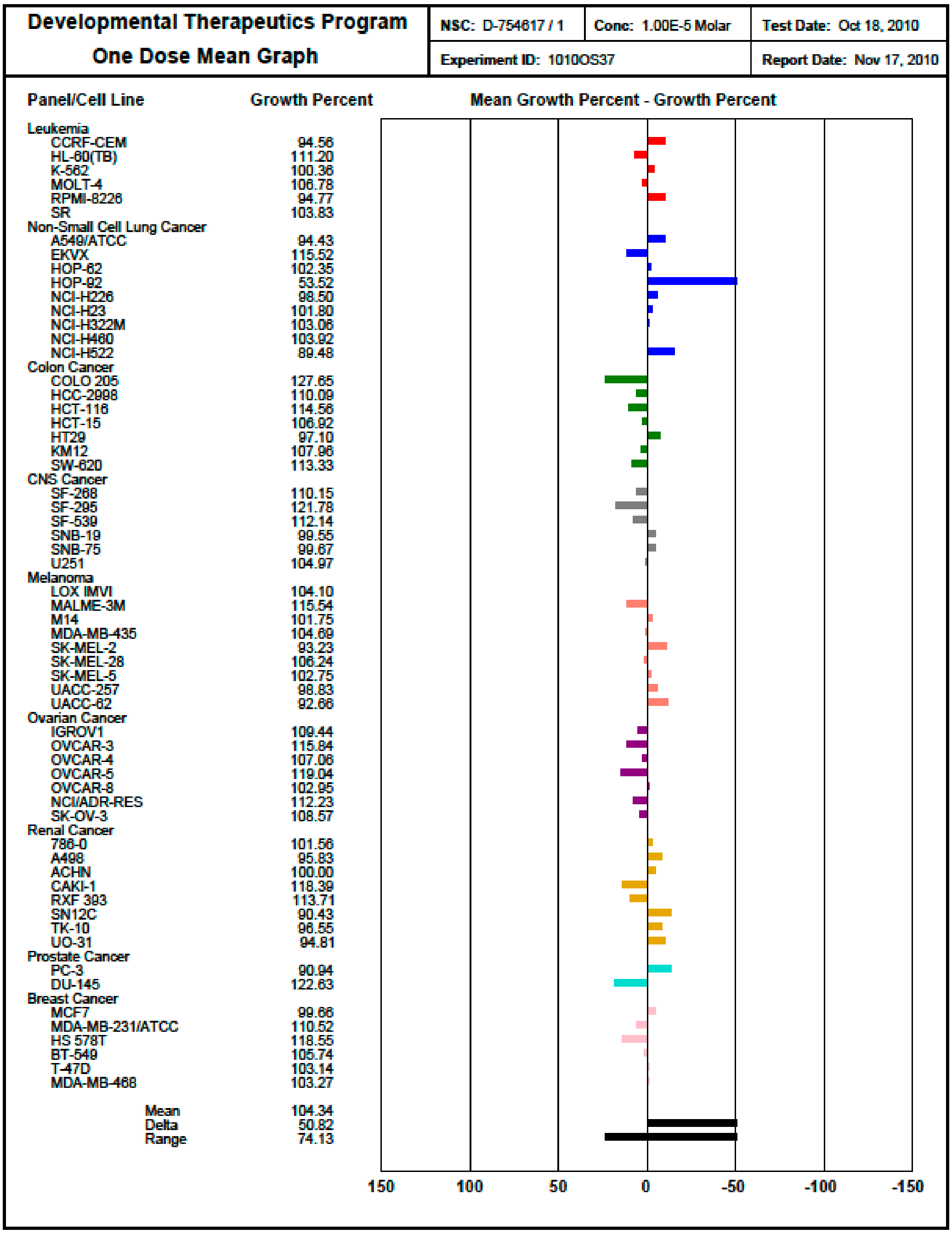
Developmental Therapeutics Program
One Dose Mean Graph
NSC: D-754617 / 1
Conc: 1.00E-5 Molar
Test Date: Oct 18, 2010
Experiment ID: 1010OS37
Report Date: Nov 17, 2010
Panel/Cell Line
Growth Percent
Mean Growth Percent - Growth Percent
Leukemia
CCRF-CEM 94.56
HL-60(TB) 111.20
K-562 100.36
MOLT-4 106.78
RPMI-8226 94.77
SR 103.83
Non-Small Cell Lung Cancer
A549/ATCC 94.43
EKVX 115.52
HOP-62 102.35
HOP-92 53.52
NCI-H226 98.50
NCI-H23 101.80
NCI-H322M 103.06
NCI-H460 103.92
NCI-H522 89.48
Colon Cancer
COLO 205 127.65
HCC-2998 110.09
HCT-116 114.56
HCT-15 106.92
HT29 97.10
KM12 107.96
SW-620 113.33
CNS Cancer
SF-268 110.15
SF-295 121.78
SF-539 112.14
SNB-19 99.55
SNB-75 99.67
U251 104.97
Melanoma
LOX IMVI 104.10
MALME-3M 115.54
M14 101.75
MDA-MB-435 104.69
SK-MEL-2 93.23
SK-MEL-28 106.24
SK-MEL-5 102.75
UACC-257 98.83
UACC-62 92.66
Ovarian Cancer
IGROV1 109.44
OVCAR-3 115.84
OVCAR-4 107.06
OVCAR-5 119.04
OVCAR-8 102.95
NCI/ADR-RES 112.23
SK-OV-3 108.57
Renal Cancer
786-0 101.56
A498 95.83
ACHN 100.00
CAKI-1 118.39
RXF 393 113.71
SN12C 90.43
TK-10 96.55
UO-31 94.81
Prostate Cancer
PC-3 90.94
DU-145 122.63
Breast Cancer
MCF7 99.66
MDA-MB-231/ATCC 110.52
HS 578T 118.55
BT-549 105.74
T-47D 103.14
MDA-MB-468 103.27
Mean 104.34
Delta 50.82
Range 74.13
150
100
50
0
-50
-100
-150

Compound 48

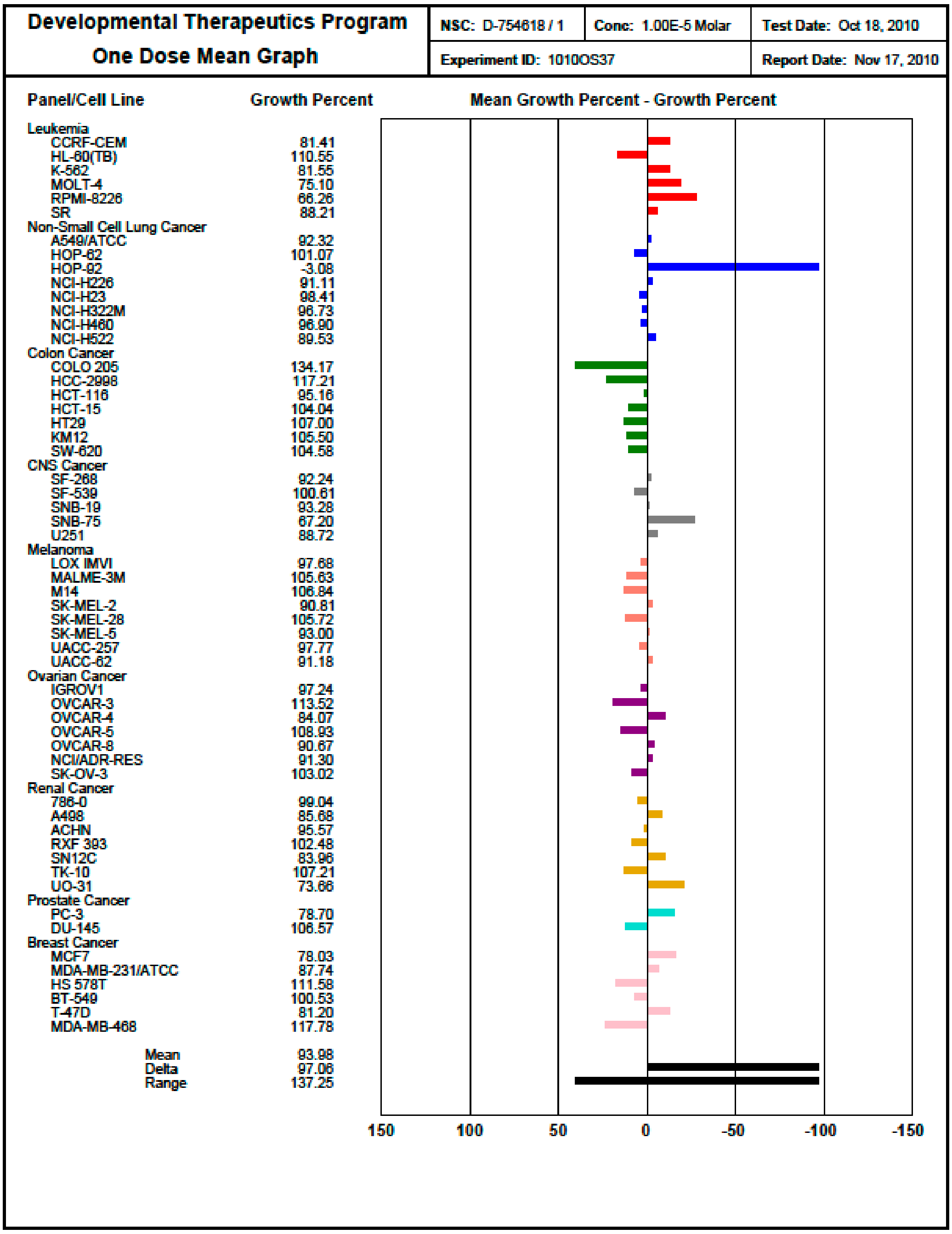
Developmental Therapeutics Program
One Dose Mean Graph
NSC: D-754618 / 1
Conc: 1.00E-5 Molar
Test Date: Oct 18, 2010
Experiment ID: 1010OS37
Report Date: Nov 17, 2010
Panel/Cell Line
Growth Percent
Mean Growth Percent - Growth Percent
Leukemia
CCRF-CEM 81.41
HL-60(TB) 110.55
K-562 81.55
MOLT-4 75.10
RPMI-8226 66.26
SR 88.21
Non-Small Cell Lung Cancer
A549/ATCC 92.32
HOP-62 101.07
HOP-92 -3.08
NCI-H226 91.11
NCI-H23 98.41
NCI-H322M 96.73
NCI-H460 96.90
NCI-H522 89.53
Colon Cancer
COLO 205 134.17
HCC-2998 117.21
HCT-116 95.16
HCT-15 104.04
HT29 107.00
KM12 105.50
SW-620 104.58
CNS Cancer
SF-268 92.24
SF-539 100.61
SNB-19 93.28
SNB-75 67.20
U251 88.72
Melanoma
LOX IMVI 97.68
MALME-3M 105.63
M14 106.84
SK-MEL-2 90.81
SK-MEL-28 105.72
SK-MEL-5 93.00
UACC-257 97.77
UACC-62 91.18
Ovarian Cancer
IGROV1 97.24
OVCAR-3 113.52
OVCAR-4 84.07
OVCAR-5 108.93
OVCAR-8 90.67
NCI/ADR-RES 91.30
SK-OV-3 103.02
Renal Cancer
786-0 99.04
A498 85.68
ACHN 95.57
RXF 393 102.48
SN12C 83.96
TK-10 107.21
UO-31 73.66
Prostate Cancer
PC-3 78.70
DU-145 106.57
Breast Cancer
MCF7 78.03
MDA-MB-231/ATCC 87.74
HS 578T 111.58
BT-549 100.53
T-47D 81.20
MDA-MB-468 117.78
Mean 93.98
Delta 97.06
Range 137.25
150
100
50
0
-50
-100
-150

Compound 49

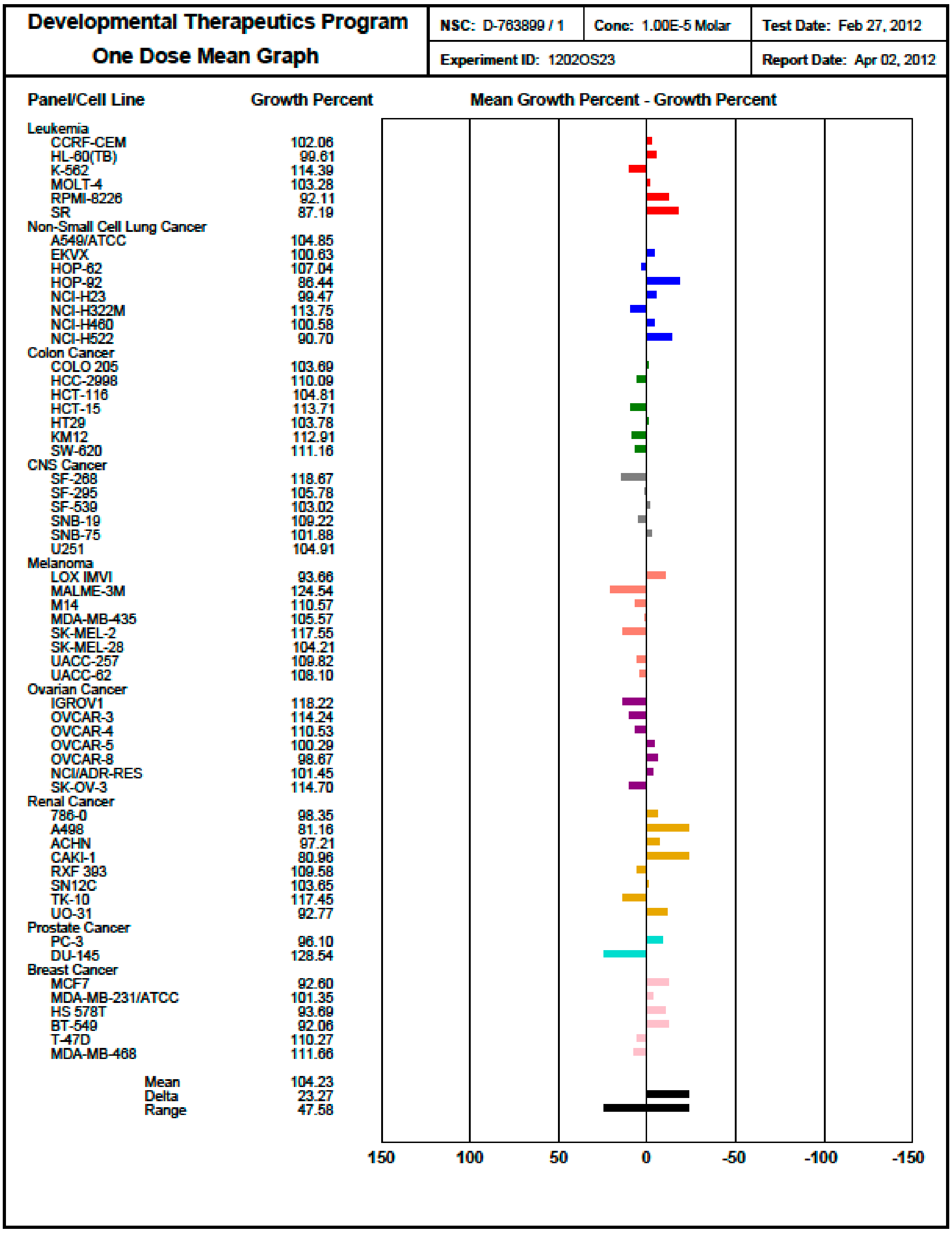
Developmental Therapeutics Program
One Dose Mean Graph
NSC: D-763899 / 1
Conc: 1.00E-5 Molar
Test Date: Feb 27, 2012
Experiment ID: 1202OS23
Report Date: Apr 02, 2012
Panel/Cell Line
Growth Percent
Mean Growth Percent - Growth Percent
Leukemia
CCRF-CEM 102.06
HL-60(TB) 99.61
K-562 114.39
MOLT-4 103.28
RPMI-8226 92.11
SR 87.19
Non-Small Cell Lung Cancer
A549/ATCC 104.85
EKVX 100.63
HOP-62 107.04
HOP-92 86.44
NCI-H23 99.47
NCI-H322M 113.75
NCI-H460 100.58
NCI-H522 90.70
Colon Cancer
COLO 205 103.69
HCC-2998 110.09
HCT-116 104.81
HCT-15 113.71
HT29 103.78
KM12 112.91
SW-620 111.16
CNS Cancer
SF-268 118.67
SF-295 105.78
SF-539 103.02
SNB-19 109.22
SNB-75 101.88
U251 104.91
Melanoma
LOX IMVI 93.66
MALME-3M 124.54
M14 110.57
MDA-MB-435 105.57
SK-MEL-2 117.55
SK-MEL-28 104.21
UACC-257 109.82
UACC-62 108.10
Ovarian Cancer
IGROV1 118.22
OVCAR-3 114.24
OVCAR-4 110.53
OVCAR-5 100.29
OVCAR-8 98.67
NCI/ADR-RES 101.45
SK-OV-3 114.70
Renal Cancer
786-0 98.35
A498 81.16
ACHN 97.21
CAKI-1 80.96
RXF 393 109.58
SN12C 103.65
TK-10 117.45
UO-31 92.77
Prostate Cancer
PC-3 96.10
DU-145 128.54
Breast Cancer
MCF7 92.60
MDA-MB-231/ATCC 101.35
HS 578T 93.69
BT-549 92.06
T-47D 110.27
MDA-MB-468 111.66
Mean 104.23
Delta 23.27
Range 47.58
150
100
50
0
-50
-100
-150

Compound 50

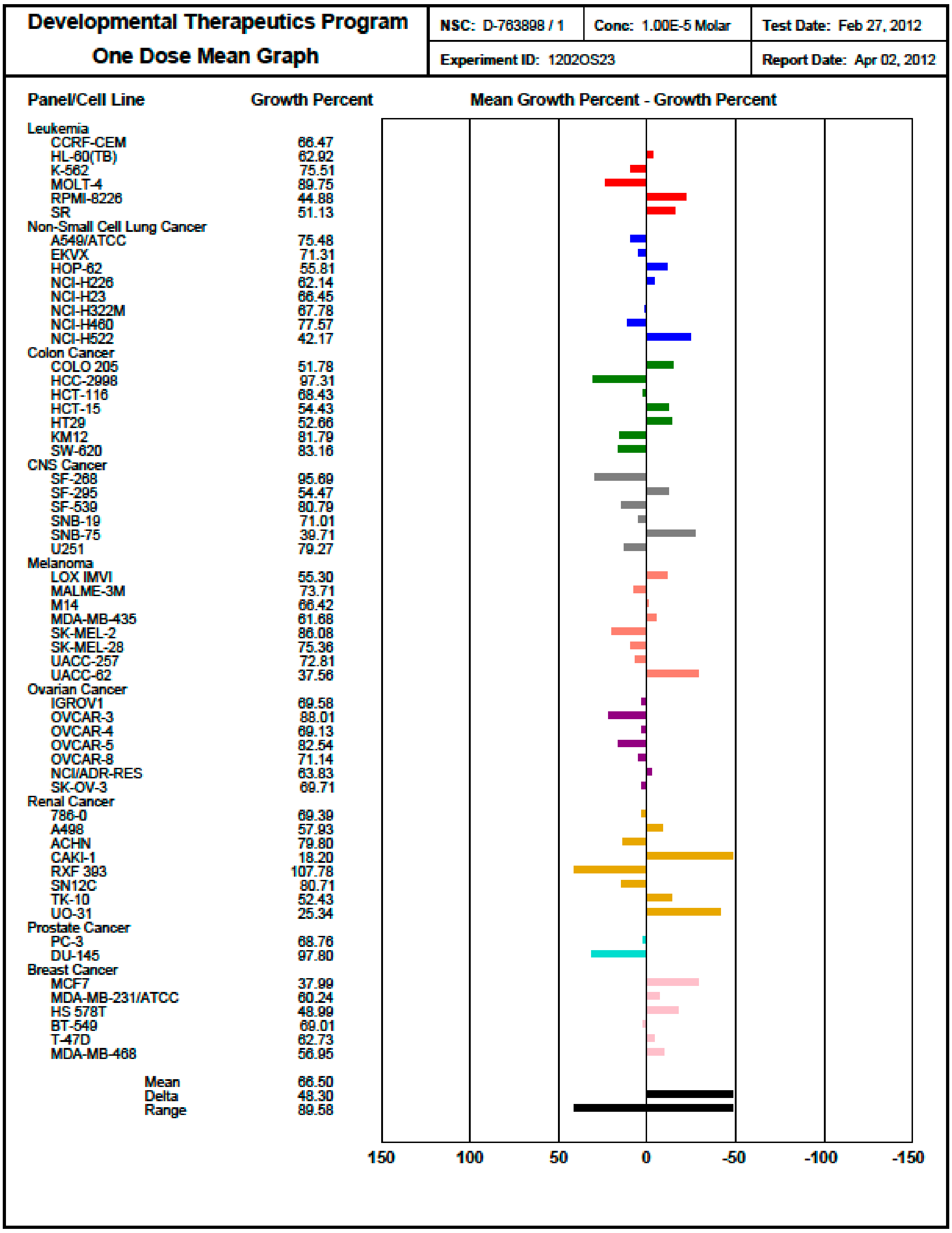
Developmental Therapeutics Program
One Dose Mean Graph
NSC: D-763898 / 1
Conc: 1.00E-5 Molar
Test Date: Feb 27, 2012
Experiment ID: 1202OS23
Report Date: Apr 02, 2012
Panel/Cell Line
Growth Percent
Mean Growth Percent - Growth Percent
Leukemia
CCRF-CEM 66.47
HL-60(TB) 62.92
K-562 75.51
MOLT-4 89.75
RPMI-8226 44.88
SR 51.13
Non-Small Cell Lung Cancer
A549/ATCC 75.48
EKVX 71.31
HOP-62 55.81
NCI-H226 62.14
NCI-H23 66.45
NCI-H322M 67.78
NCI-H460 77.57
NCI-H522 42.17
Colon Cancer
COLO 205 51.78
HCC-2998 97.31
HCT-116 68.43
HCT-15 54.43
HT29 52.66
KM12 81.79
SW-620 83.16
CNS Cancer
SF-268 95.69
SF-295 54.47
SF-539 80.79
SNB-19 71.01
SNB-75 39.71
U251 79.27
Melanoma
LOX IMVI 55.30
MALME-3M 73.71
M14 66.42
MDA-MB-435 61.68
SK-MEL-2 86.08
SK-MEL-28 75.36
UACC-257 72.81
UACC-62 37.56
Ovarian Cancer
IGROV1 69.58
OVCAR-3 88.01
OVCAR-4 69.13
OVCAR-5 82.54
OVCAR-8 71.14
NCI/ADR-RES 63.83
SK-OV-3 69.71
Renal Cancer
786-0 69.39
A498 57.93
ACHN 79.80
CAKI-1 18.20
RXF 393 107.78
SN12C 80.71
TK-10 52.43
UO-31 25.34
Prostate Cancer
PC-3 68.76
DU-145 97.80
Breast Cancer
MCF7 37.99
MDA-MB-231/ATCC 60.24
HS 578T 48.99
BT-549 69.01
T-47D 62.73
MDA-MB-468 56.95
Mean 66.50
Delta 48.30
Range 89.58
150
100
50
0
-50
-100
-150

Compound 51

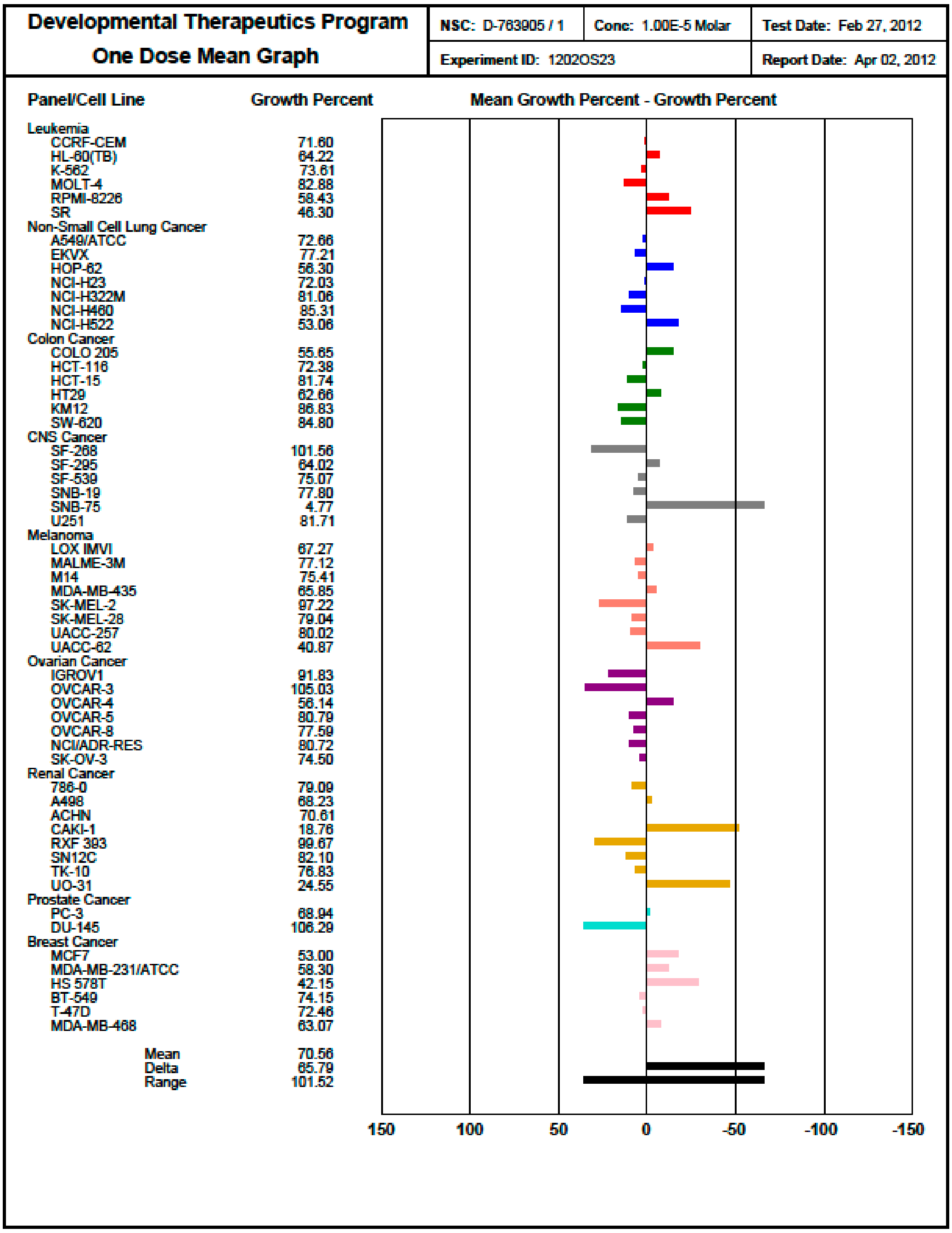
Developmental Therapeutics Program
One Dose Mean Graph
NSC: D-763905 / 1
Conc: 1.00E-5 Molar
Test Date: Feb 27, 2012
Experiment ID: 1202OS23
Report Date: Apr 02, 2012
Panel/Cell Line
Growth Percent
Mean Growth Percent - Growth Percent
Leukemia
CCRF-CEM 71.60
HL-60(TB) 64.22
K-562 73.61
MOLT-4 82.88
RPMI-8226 58.43
SR 46.30
Non-Small Cell Lung Cancer
A549/ATCC 72.66
EKVX 77.21
HOP-62 56.30
NCI-H23 72.03
NCI-H322M 81.06
NCI-H460 85.31
NCI-H522 53.06
Colon Cancer
COLO 205 55.65
HCT-116 72.38
HCT-15 81.74
HT29 62.66
KM12 86.83
SW-620 84.80
CNS Cancer
SF-268 101.56
SF-295 64.02
SF-539 75.07
SNB-19 77.80
SNB-75 4.77
U251 81.71
Melanoma
LOX IMVI 67.27
MALME-3M 77.12
M14 75.41
MDA-MB-435 65.85
SK-MEL-2 97.22
SK-MEL-28 79.04
UACC-257 80.02
UACC-62 40.87
Ovarian Cancer
IGROV1 91.83
OVCAR-3 105.03
OVCAR-4 56.14
OVCAR-5 80.79
OVCAR-8 77.59
NCI/ADR-RES 80.72
SK-OV-3 74.50
Renal Cancer
786-0 79.09
A498 68.23
ACHN 70.61
CAKI-1 18.76
RXF 393 99.67
SN12C 82.10
TK-10 76.83
UO-31 24.55
Prostate Cancer
PC-3 68.94
DU-145 106.29
Breast Cancer
MCF7 53.00
MDA-MB-231/ATCC 58.30
HS 578T 42.15
BT-549 74.15
T-47D 72.46
MDA-MB-468 63.07
Mean 70.56
Delta 65.79
Range 101.52
150
100
50
0
-50
-100
-150

Compound 52

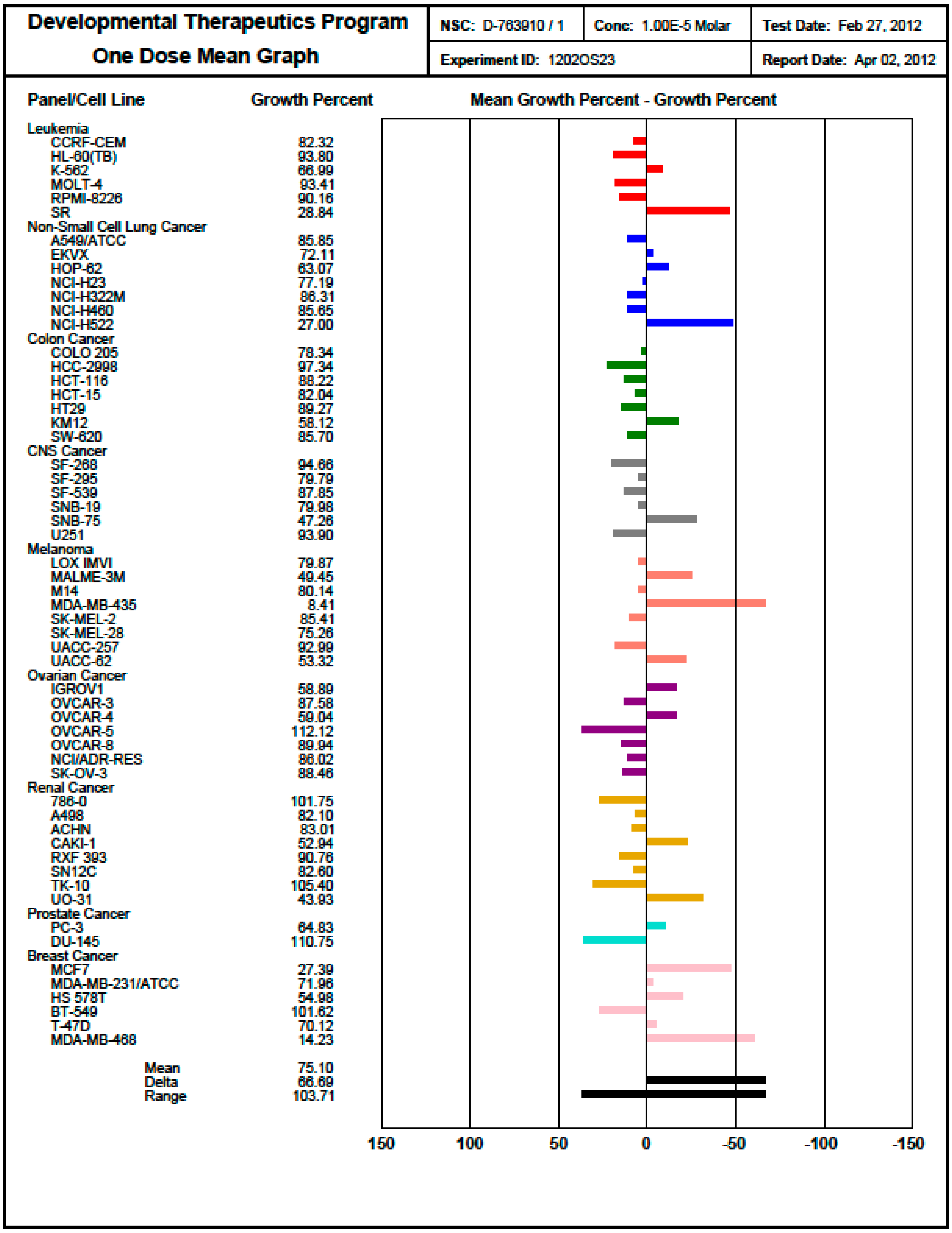
Developmental Therapeutics Program
One Dose Mean Graph
NSC: D-763910 / 1
Conc: 1.00E-5 Molar
Test Date: Feb 27, 2012
Experiment ID: 1202OS23
Report Date: Apr 02, 2012
Panel/Cell Line
Growth Percent
Mean Growth Percent - Growth Percent
Leukemia
CCRF-CEM 82.32
HL-60(TB) 93.80
K-562 66.99
MOLT-4 93.41
RPMI-8226 90.16
SR 28.84
Non-Small Cell Lung Cancer
A549/ATCC 85.85
EKVX 72.11
HOP-62 63.07
NCI-H23 77.19
NCI-H322M 86.31
NCI-H460 85.65
NCI-H522 27.00
Colon Cancer
COLO 205 78.34
HCC-2998 97.34
HCT-116 88.22
HCT-15 82.04
HT29 89.27
KM12 58.12
SW-620 85.70
CNS Cancer
SF-268 94.66
SF-295 79.79
SF-539 87.85
SNB-19 79.98
SNB-75 47.26
U251 93.90
Melanoma
LOX IMVI 79.87
MALME-3M 49.45
M14 80.14
MDA-MB-435 8.41
SK-MEL-2 85.41
SK-MEL-28 75.26
UACC-257 92.99
UACC-62 53.32
Ovarian Cancer
IGROV1 58.89
OVCAR-3 87.58
OVCAR-4 59.04
OVCAR-5 112.12
OVCAR-8 89.94
NCI/ADR-RES 86.02
SK-OV-3 88.46
Renal Cancer
786-0 101.75
A498 82.10
ACHN 83.01
CAKI-1 52.94
RXF 393 90.76
SN12C 82.60
TK-10 105.40
UO-31 43.93
Prostate Cancer
PC-3 64.83
DU-145 110.75
Breast Cancer
MCF7 27.39
MDA-MB-231/ATCC 71.96
HS 578T 54.98
BT-549 101.62
T-47D 70.12
MDA-MB-468 14.23
Mean 75.10
Delta 66.69
Range 103.71
150
100
50
0
-50
-100
-150

Compound 53

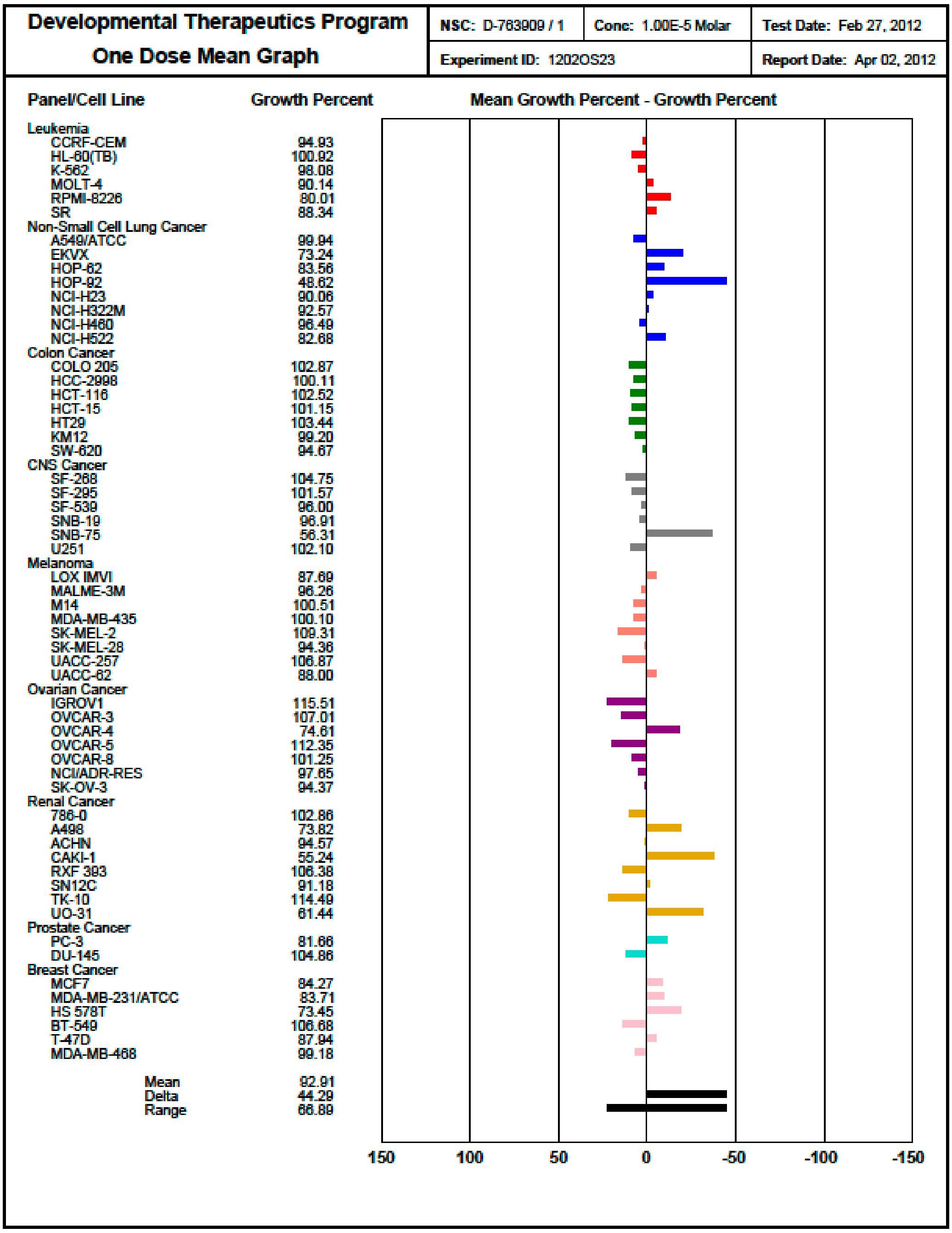
Developmental Therapeutics Program
One Dose Mean Graph
NSC: D-763909 / 1
Conc: 1.00E-5 Molar
Test Date: Feb 27, 2012
Experiment ID: 1202OS23
Report Date: Apr 02, 2012
Panel/Cell Line
Growth Percent
Mean Growth Percent - Growth Percent
Leukemia
CCRF-CEM 94.93
HL-60(TB) 100.92
K-562 98.08
MOLT-4 90.14
RPMI-8226 80.01
SR 88.34
Non-Small Cell Lung Cancer
A549/ATCC 99.94
EKVX 73.24
HOP-62 83.56
HOP-92 48.62
NCI-H23 90.06
NCI-H322M 92.57
NCI-H460 96.49
NCI-H522 82.68
Colon Cancer
COLO 205 102.87
HCC-2998 100.11
HCT-116 102.52
HCT-15 101.15
HT29 103.44
KM12 99.20
SW-620 94.67
CNS Cancer
SF-268 104.75
SF-295 101.57
SF-539 96.00
SNB-19 96.91
SNB-75 56.31
U251 102.10
Melanoma
LOX IMVI 87.69
MALME-3M 96.26
M14 100.51
MDA-MB-435 100.10
SK-MEL-2 109.31
SK-MEL-28 94.36
UACC-257 106.87
UACC-62 88.00
Ovarian Cancer
IGROV1 115.51
OVCAR-3 107.01
OVCAR-4 74.61
OVCAR-5 112.35
OVCAR-8 101.25
NCI/ADR-RES 97.65
SK-OV-3 94.37
Renal Cancer
786-0 102.86
A498 73.82
ACHN 94.57
CAKI-1 55.24
RXF 393 106.38
SN12C 91.18
TK-10 114.49
UO-31 61.44
Prostate Cancer
PC-3 81.66
DU-145 104.86
Breast Cancer
MCF7 84.27
MDA-MB-231/ATCC 83.71
HS 578T 73.45
BT-549 106.68
T-47D 87.94
MDA-MB-468 99.18
Mean 92.91
Delta 44.29
Range 66.89
150
100
50
0
-50
-100
-150

Compound 54

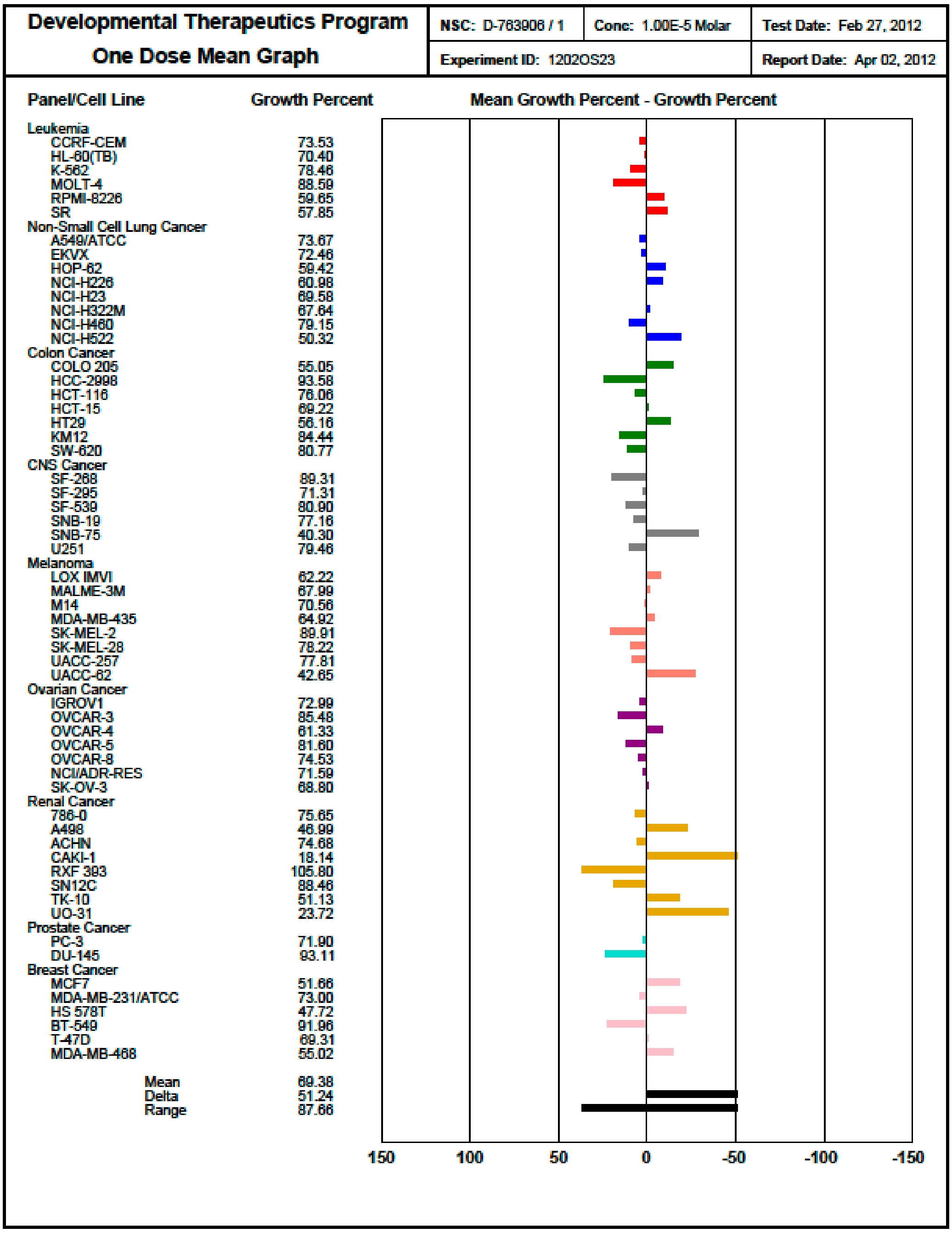
Developmental Therapeutics Program
One Dose Mean Graph
NSC: D-763906 / 1
Conc: 1.00E-5 Molar
Test Date: Feb 27, 2012
Experiment ID: 1202OS23
Report Date: Apr 02, 2012
Panel/Cell Line
Growth Percent
Mean Growth Percent - Growth Percent
Leukemia
CCRF-CEM 73.53
HL-60(TB) 70.40
K-562 78.46
MOLT-4 88.59
RPMI-8226 59.65
SR 57.85
Non-Small Cell Lung Cancer
A549/ATCC 73.67
EKVX 72.46
HOP-62 59.42
NCI-H226 60.98
NCI-H23 69.58
NCI-H322M 67.64
NCI-H460 79.15
NCI-H522 50.32
Colon Cancer
COLO 205 55.05
HCC-2998 93.58
HCT-116 76.06
HCT-15 69.22
HT29 56.16
KM12 84.44
SW-620 80.77
CNS Cancer
SF-268 89.31
SF-295 71.31
SF-539 80.90
SNB-19 77.16
SNB-75 40.30
U251 79.46
Melanoma
LOX IMVI 62.22
MALME-3M 67.99
M14 70.56
MDA-MB-435 64.92
SK-MEL-2 89.91
SK-MEL-28 78.22
UACC-257 77.81
UACC-62 42.65
Ovarian Cancer
IGROV1 72.99
OVCAR-3 85.48
OVCAR-4 61.33
OVCAR-5 81.60
OVCAR-8 74.53
NCI/ADR-RES 71.59
SK-OV-3 68.80
Renal Cancer
786-0 75.65
A498 46.99
ACHN 74.68
CAKI-1 18.14
RXF 393 105.80
SN12C 88.46
TK-10 51.13
UO-31 23.72
Prostate Cancer
PC-3 71.90
DU-145 93.11
Breast Cancer
MCF7 51.66
MDA-MB-231/ATCC 73.00
HS 578T 47.72
BT-549 91.96
T-47D 69.31
MDA-MB-468 55.02
Mean 69.38
Delta 51.24
Range 87.66
150
100
50
0
-50
-100
-150

Compound 55

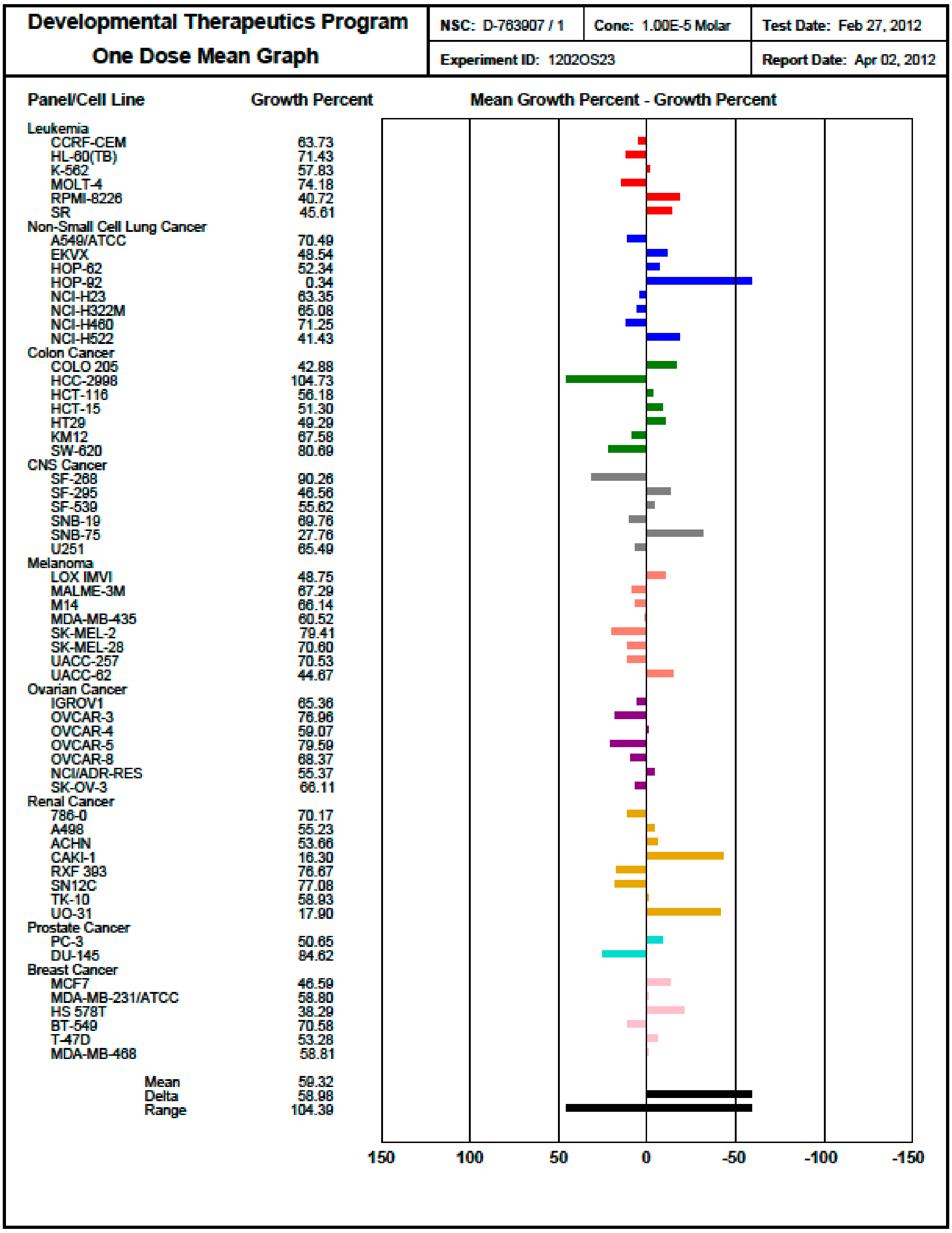
Developmental Therapeutics Program
One Dose Mean Graph
NSC: D-763907 / 1
Conc: 1.00E-5 Molar
Test Date: Feb 27, 2012
Experiment ID: 1202OS23
Report Date: Apr 02, 2012
Panel/Cell Line
Growth Percent
Mean Growth Percent - Growth Percent
Leukemia
CCRF-CEM 63.73
HL-60(TB) 71.43
K-562 57.83
MOLT-4 74.18
RPMI-8226 40.72
SR 45.61
Non-Small Cell Lung Cancer
A549/ATCC 70.49
EKVX 48.54
HOP-62 52.34
HOP-92 0.34
NCI-H23 63.35
NCI-H322M 65.08
NCI-H460 71.25
NCI-H522 41.43
Colon Cancer
COLO 205 42.88
HCC-2998 104.73
HCT-116 56.18
HCT-15 51.30
HT29 49.29
KM12 67.58
SW-620 80.69
CNS Cancer
SF-268 90.26
SF-295 46.56
SF-539 55.62
SNB-19 69.76
SNB-75 27.76
U251 65.49
Melanoma
LOX IMVI 48.75
MALME-3M 67.29
M14 66.14
MDA-MB-435 60.52
SK-MEL-2 79.41
SK-MEL-28 70.60
UACC-257 70.53
UACC-62 44.67
Ovarian Cancer
IGROV1 65.36
OVCAR-3 76.96
OVCAR-4 59.07
OVCAR-5 79.59
OVCAR-8 68.37
NCI/ADR-RES 55.37
SK-OV-3 66.11
Renal Cancer
786-0 70.17
A498 55.23
ACHN 53.66
CAKI-1 16.30
RXF 393 76.67
SN12C 77.08
TK-10 58.93
UO-31 17.90
Prostate Cancer
PC-3 50.65
DU-145 84.62
Breast Cancer
MCF7 46.59
MDA-MB-231/ATCC 58.80
HS 578T 38.29
BT-549 70.58
T-47D 53.28
MDA-MB-468 58.81
Mean 59.32
Delta 58.98
Range 104.39
150
100
50
0
-50
-100
-150

Table of activity for Topo II decatenation assay

Table 1. Topo II decatenation assay: (+) inhibition at 100 μM; (−) no inhibition at 100, 10 and 1 μM.

Structure	Compound	Activity
	**Ellipticine**	+
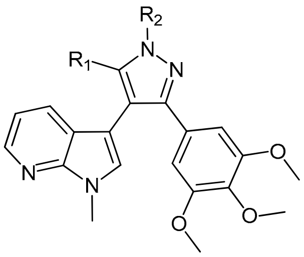	**46**; R_1=NH_2, R_2=H	−
	**47**; -NHCONHCO-	−
	**48**; -NC(CF_3)CHC(CF_3)-	−
	**49**; R_1=NH_2, R_2=$COCH_3$	−
	**50**; R_1=NH_2, R_2=$CSNHCH_3$	−

References

1. Eccles, K.S.; Stokes, S.P.; Daly, C.A.; Barry, N.M.; McSweeney, S.P.; O' Neill, D.J.; Kelly, D.M.; Jennings, W.B.; Ní Dhubhghaill, O.M.; Moynihan, H.A.; et. al. Evaluation of the bruker smart X2S: Crystallography for the nonspecialist?. *J. Appl. Cryst.* **2011**, *44*, 213–215.
2. Sheldrick, G.M. A short history of SHELX *Acta Crystallogr.* **2008**, *A64,* 112–122.
3. APEX2, v2009.3–0; Bruker AXS: Madison, WI, USA, 2009.
4. Narumiya, S.; Takai, K.; Tokuyama, T.; Noda, Y.; Ushiro, H.; Hayaishi, O. A new metabolic pathway of tryptophan initiated by tryptophan side chain oxidase. *J. Biol. Chem.* **1979**, *254*, 7007–7015.
5. Blunt, J.W.; Erasmuson, A.F.; Ferrier, R.J.; Munro, M.H.G. Syntheses of haptens related to the benzenoid and indole portions of sporidesmin a; 13c nmr spectra of indole derivatives. *Aust. J. Chem.* **1979**, *32*, 1045–1054.
